# Mindfulness-Based and Mindfulness-Informed Interventions at the Workplace: A Systematic Review and Meta-Regression Analysis of RCTs

**DOI:** 10.1007/s12671-023-02130-7

**Published:** 2023-05-11

**Authors:** Maren M. Michaelsen, Johannes Graser, Miriam Onescheit, Matthias P. Tuma, Lena Werdecker, Dawid Pieper, Tobias Esch

**Affiliations:** 1 Institute for Integrative Health Care and Health Promotion, Faculty of Health, Witten/Herdecke University, Witten, Germany; 2 Department of Psychology and Psychotherapy, Faculty of Health, Witten/Herdecke University, Witten, Germany; 3 Faculty of Health Sciences Brandenburg, Brandenburg Medical School (Theodor Fontane), Institute for Health Services and Health System Research, Rüdersdorf, Germany; 4 Center for Health Services Research, Brandenburg Medical School (Theodor Fontane), Rüdersdorf, Germany

**Keywords:** Mindfulness-based interventions, Mindfulness-informed interventions, Occupational health, Systematic review, Meta-analysis

## Abstract

**Objectives:**

Positive effects of mindfulness-based interventions (MBIs) on occupational health have been demonstrated by several systematic review studies during the last two decades. So far, existing reviews excluded mindfulness-informed interventions (MIIs) that build on informal approaches or mixed techniques aiming at improving mindfulness indirectly. To address this research gap, the present comprehensive meta-analysis synthesizes the results of RCTs of MBIs and MIIs conducted in various workplace settings.

**Method:**

A systematic literature search was conducted in five electronic databases complemented by manual search. Random-effects models were used to synthesize standardized mean differences (*SMDs*) for 25 outcomes and seven overarching categories of outcomes, and to detect various temporal effects. Meta-regressions were run to elucidate average *SMDs* between mindfulness intervention types and intervention and population characteristics, with the goal of detecting sources of heterogeneity and help guide the selection of the most appropriate mindfulness intervention type.

**Results:**

Based on 91 eligible studies (from 92 publications), including 4927 participants and 4448 controls, the synthesis shows that MBIs and MIIs significantly improve mindfulness (*SMD* = 0.43; 95%-*CI* [0.33;0.52]), well-being (*SMD* = 0.63; 95%-*CI* [0.34;0.93]), mental health (*SMD* = 0.67; 95%-*CI* [0.48;0.86]), stress (*SMD* = 0.72; 95%-*CI* [0.54;0.90]), resilience (*SMD* = 1.06; 95%-*CI* [−0.22;2.34]), physical health (*SMD* = 0.45; 95%-*CI* [0.32;0.59]), and work-related factors (*SMD* = 0.62; 95%-*CI* [0.14;1.10]). Sensitivity analyses demonstrate a tendency towards smaller effect sizes due to extreme outliers. Effect sizes are stable in short-term follow-up assessments (1-12 weeks) for most outcomes, but not for long-term follow-up assessments (13-52 weeks). Meta-regressions suggest that observable intervention characteristics (e.g., online delivery) and population characteristics (e.g., age of participants), as well as study quality, do not explain the prevalence of heterogeneity in effect sizes.

**Conclusions:**

Generally effective, mindfulness interventions are a useful tool to enhance aspects of employee health. However, because of heterogeneity and risk of bias, studies aiming at high-quality data collection and thorough reporting are necessary to draw firm conclusions.

**Preregistration:**

A protocol of this systematic review was registered with PROSPERO (Registration-No. CRD42020159927).

**Supplementary Information:**

The online version contains supplementary material available at 10.1007/s12671-023-02130-7.

Mind-body interventions are based on the concept that the mind and body are interconnected and mutually influence one another (Esch & Brinkhaus, 2020). These interventions have been found to reduce stress and promote overall health and productivity, often by increasing mindfulness among those who practice them (Esch & Brinkhaus, 2021). Mindfulness is understood as the intentional focus on bodily sensations, emotions, and thoughts, with the aim of developing awareness and nonjudgmental acceptance of these experiences (Grossman, 2015). Mindfulness interventions are gaining popularity in public and private organizations, where employees and leaders are increasingly encouraged to improve their mindfulness levels directly, or indirectly, through a variety of interventions (Vonderlin et al., 2020).

The scientific literature has identified a range of positive outcomes associated with mindfulness practice. For example, improving mindfulness promotes attention regulation, body awareness, emotion regulation, and self-awareness through neurological or neurophysiological changes (Esch, 2014; Hölzel et al., 2011) beyond the well-known reduction of stress. These skills can contribute to, e.g., enhanced well-being and other beneficial resources (Gu et al., 2015). Training in mindfulness can be facilitated through a variety of formal and informal techniques, as well as through programs that combine several exercises. These techniques can be defined as a family of “complex emotion- and attention-regulatory trainings” that are applied to achieve a variety of goals such as the development of well-being and emotional balance (Lutz et al., 2008, p. 163). These techniques, or combinations of techniques, can be categorized as either mindfulness-based interventions (MBIs) or mindfulness-informed interventions (MIIs) (Michaelsen et al., 2021). MBIs typically focus on learning or improving mindfulness as the main mechanism of action and involve formal mental exercises, such as breathing meditation or body scan. The particularly well-known “Mindfulness-Based Stress Reduction” (MBSR) program developed by Kabat-Zinn (1982), for example, is one of these methods. These formal techniques also influence so-called “informal mindfulness” through training effects established during the course of the program. These training effects include, e.g., increased attention in the present moment, an aspect that is also becoming more and more prevalent in everyday activities (Birtwell et al., 2019). Thus, effects derived from formal mindfulness concepts also increasingly influence informal components (Crane et al., 2014).

MIIs are influenced by the practice and philosophy of mindfulness, as well as other methodologies, that shape programs that are geared towards achieving specific outcomes, such as improving communication skills or physical flexibility (Crane et al., 2017; Griffith et al., 2019). These approaches promote mindfulness indirectly in a variety of ways, for example by means of breathing exercises or mindful movement sequences found in practices like *yoga*, *tai chi*, or *qigong* (Gaiswinkler & Unterrainer, 2016; Shelov et al., 2009). MIIs further include techniques that focus on promoting relaxation, acceptance or communication and do not exclusively utilize mental or formal mindfulness exercises for this purpose (Crane et al., 2017; Esch, 2020). The key difference is that MBIs place greater emphasis on cultivating mindfulness and present-moment awareness, while MIIs use mindfulness practices as one tool among others to achieve specific goals.

The distinction between MBIs and MIIs can be explained by the example of breathing-related exercises. The aforementioned breathing meditation is a mindfulness-based practice that involves focusing one’s attention on the breath, observing the natural flow of the breath and the sensations associated with it, and returning one’s attention to the breath whenever the mind wanders. The aim of breathing meditation is to cultivate present-moment awareness and develop greater concentration and mental clarity. Breathing exercises, on the other hand, are mindfulness-informed practices that may involve focusing on the breath, but are typically more structured and goal-oriented. Breathing exercises often involve specific patterns of inhaling and exhaling, such as deep breathing or alternate nostril breathing, and may be used to achieve specific physiological or psychological effects, such as stress reduction or improving lung functioning. The difference between these two practices is that breathing meditations are primarily focused on developing mindfulness and present-moment awareness, while breathing exercises are primarily focused on achieving specific physical or psychological outcomes. Yet, by engaging in breathing exercises, and its steady focus on the repetitive breathing manipulation, mindfulness can be improved indirectly.

The field of meditation and mindfulness interventions has experienced a rapid expansion in both general and medical contexts, as evidenced by the growing number of intervention studies and reviews (Creswell, 2017). There are a number of systematic reviews on the overall effectiveness of mindfulness programs in general (Eberth & Sedlmeier, 2012; Goyal et al., 2014; Ospina et al., 2007; Querstret et al., 2020), for mental disorders (Sedlmeier et al., 2012; Vancampfort et al., 2021; Virgili, 2015) and for specific medical indications, e.g., chronic pain or breast cancer (Crain et al., 2017; Cramer et al., 2012a, b; Khoo et al., 2019). Systematic literature reviews on specific additional aspects exist, for example demonstrating the effectiveness of mindfulness training or meditation in changing neuronal structures (Fox et al., 2014; Gotink et al., 2016; Wang et al., 2021).

Systematic reviews and meta-analyses of mindfulness studies in the work context have demonstrated that both deficit-oriented parameters, such as burnout and depression, as well as resource-oriented parameters, such as job satisfaction and work performance, can be improved through mindfulness-based programs (Bartlett et al., 2019; Lomas et al., 2019; Richardson & Rothstein, 2008; Vonderlin et al., 2020). The evidence varies, however, from review to review and several questions regarding the impact of mindfulness training in the work context remain unanswered. Specifically, it is unclear what aspects, e.g., health, well-being, or work-specific factors, can be improved by which type of mindfulness training. Furthermore, the overall effectiveness has not yet been conclusively clarified, nor have distinct features of interventions, e.g., homework, or the duration of the interventions, been fully evaluated. In addition, existing reviews of mindfulness interventions in workplace settings have focused primarily on mindfulness-based interventions, with relatively little attention given to mindfulness-informed approaches.

Mindfulness interventions can be delivered in various formats, including group-based sessions with face-to-face interaction between trainers and participants, or individual sessions, where a trainer works on a one-on-one basis with an employee. Additionally, both types of teachings can be delivered through digital platforms, e.g., online seminars, webinars, or mindfulness apps. The use of digital formats to promote health and well-being in the workplace has been increasing even prior to the COVID-19 pandemic, as evidenced by the growing amount of studies investigating digital formats (e.g., Bostock et al., 2019; Coelhoso et al., 2019; Lilly et al., 2019).

Our present work aims to provide a comprehensive statistical evaluation of a wide body of studies on MBIs and MIIs at the workplace which have been published since the analyses by Ospina et al. (2007, 2008). Due to a strong increase in the number of publications on MBIs in the work context, a renewed analysis is deemed appropriate and timely. In addition, to the best of our awareness, this is the first review to incorporate mindfulness-informed, rather than exclusively mindfulness-based, interventions. Expanding the scope of interventions studied is important, as there are a substantial number of intervention programs conducted in the real world for which there is currently no collective evidence of their effectiveness.

In this review, we pursued four main objectives. Firstly, we aimed to review both mindfulness-based and mindfulness-informed workplace interventions, and to identify their average effect sizes on well-being-, health-, and work-related outcomes. To achieve this objective, we analyzed studies in context of intervention types and outcome categories, and performed meta-regression analyses to provide additional analysis at a medium level of granularity. Secondly, we aimed to investigate the strength of the effects over time by looking at short- and long-term follow-up data. Thirdly, we aimed to identify the potential influence of different delivery modes of workplace mindfulness interventions, including online vs. analogue, and in-group vs. independent practice. Finally, we examined whether specific training characteristics, such as homework and intervention duration, correlate with the strength of the interventions’ effects.

These aspects are particularly relevant when translating research into practice. As outlined above, the last two decades of research on mindfulness interventions demonstrated beneficial effects in various settings. Yet, employers increasingly face the challenge of identifying offers that are both effective and resource-oriented, while also being well-received by employees. Self-guided and homework-based interventions are attractive formats of delivery from an employer’s perspective, but there are concerns that they may overly dilute well-established and proven mindfulness programs. As part of our comprehensive analyses, the present study provides insight into both lines of arguments with concrete practical relevance to employers interested in providing MBIs or MIIs at the workplace.

## Method

The reporting of this systematic review and meta-analysis followed the expanded PRISMA checklist 2020 (Page et al., 2021) (see Supplementary Online Material, Table S1.1). As recommended by Cochrane (Lefebvre et al., 2021), all steps of study selection, data extraction and risk of bias assessment were conducted independently by two authors. Conflicts were discussed between the two researchers and unresolved conflicts were solved by a third researcher.

### Search Strategy

In order to identify all relevant studies, a systematic literature search was conducted in PubMed, PubPsych and PsycInfo, Scopus, and Cochrane CENTRAL in November 2019. The full search strategy can be found in Table S2.1. The following eligibility criteria were defined for the selection of relevant studies: Due to the large number of published studies on mindfulness in the workplace, only studies based on a randomized controlled trial (RCT) design were included in this review. RCTs with all types of control groups were included. Only individually randomized controlled trials were included; i.e., cluster-randomized studies were excluded, in order to increase comparability between study results. Specifically, interventions were required to be based on the central guiding principle of promoting mindfulness, and this had to be clearly recognizable in the description of the study. This means that all intervention descriptions mentioned either the explicit aim of increasing mindfulness or self-awareness (e.g., of bodily sensations, affect, or thoughts), or used mindfulness techniques to achieve other outcomes. Hence, in addition to classical MBSR or meditation interventions, we included interventions which are based on mindfulness-informed practices, such as *yoga*, acceptance and commitment therapy (ACT), or breathing exercises. The number or share of hours in the intervention related to mindfulness was irrelevant (as compared to Vonderlin et al., 2020), as long as mindfulness or its functions (e.g., increasing bodily awareness) was mentioned in the description of the intervention. Excluded are studies that examined health promotion on, for example, the basis of positive psychology (e.g., Feicht et al., 2013), but which did not pursue mindfulness as a central guiding principle. These inclusion criteria allow both mindfulness-based and mindfulness-informed interventions to be considered. We included only studies that analyzed mindfulness interventions that were offered in the workplace or initiated by the employing organization, with working adults as the target population. Another requirement for the inclusion of a study was its (online) publication between January 2005 and November 2019 in German or English language peer-reviewed scientific journals. The choice of the start date is based on the search period of the review by Ospina et al. (2007, 2008), which is the first comprehensive review on mindfulness interventions and serves as an important knowledge base for the current review. In addition, the two previous reviews by Bartlett et al. (2019) and Lomas et al. (2019) have not found any workplace mindfulness interventions published before 2005. Supplementary material of retrieved studies was screened for information, and study protocols were downloaded if available. Authors were contacted by e-mail for additional information, such as means (M), standard deviations (SD), and number of participants (N) of intervention and control groups after the intervention, if not available in the authors’ publications. These data were mandatory in order to determine whether studies could be included in the present meta-analysis. Conference abstracts were not included. The Rayyan software (rayyan.ai) was used to collect all search results and to screen titles and abstracts.

### Data Extraction

Data extracted from the articles to a central Excel file included name of the intervention (original title), type of control group (active, passive or waiting list), mode of delivery of the mindfulness training, including in-class vs. individual training, online vs. offline, (additional) one-on-one support, the use of additional material, total duration of the intervention in hours and weeks, training location (at the workplace, centralized at a location outside the workplace, location-independent), whether the training took place during or outside working hours, and whether homework was compulsory. In addition, the number of participants per group at different measurement times, the mean age of participants, and the share of female participants were extracted. Furthermore, we extracted the country in which the intervention took place and the occupation of the study population. We noted means and standard deviations of all outcomes and the specific instruments used at all time points, i.e., pre-intervention (before = T0), post-intervention (immediately after the end of the intervention = T1), and all follow-ups. Follow-ups were aggregated into two time periods for analysis, namely 1 to 12 weeks (short-term) and 13 to 52 weeks (long-term). If studies had collected data more than once within these time periods, we chose to include the data referring to the shorter time period in our analysis. Some articles contain intermediate results, i.e., those collected during (e.g., in the middle of) the intervention. These were not extracted for the present study due to limited means of comparability. We also used timing of data collection as a continuous variable to detect the influence of time since the end of the intervention on intervention effectiveness. We analyzed only those outcomes that had been investigated by at least four of the identified studies. This decision on the minimum number of four studies per outcomes is in line with other reviews: Lomas et al. (2019) evaluated results from at least five studies, Bartlett et al. (2019) defined a minimum number of three studies, and Vonderlin et al. (2020) evaluated parameters represented by at least four studies. In this way, a balance is sought between the representation of the versatility of the effects of mindfulness interventions and the individual significance of the outcomes. Aspects examined in only few cases, such as aggression, fatigue, cognition, and various physiological markers, have therefore been omitted. Outcomes are aggregated into broad categories as explained below.

To assess the risk of bias, we calculated a dropout rate based on the reported numbers of observations in the texts. When dropout rates were not provided at all measurement points in the publication, we assumed no drop-outs occurred during the intervention. It is important to acknowledge that some articles contain contradictory information. To address this, we extracted either the most plausible or the most frequently mentioned information. If there was no information available, the gap was marked as “na” (not available).

### Outcome Measures

The unexpected high number of studies including more than 400 different instruments (physiological outcomes, questionnaires, VAS, etc.), which were detected in the screening process, required categorization of outcomes. Therefore, all outcome measures were grouped into seven overarching categories with a total of 25 detailed subcategories. These categories are similar to those in Bartlett et al. (2019), and Vonderlin et al. (2020), and are outlined as followed and defined in Table S3.1. (1) *Mindfulness* is a parameter that is self-assessed through different questionnaires, such as the Five Facets Mindfulness Questionnaire, (2) *Well-being* parameters are comprised of self-assessed life satisfaction, relaxation ability/state of relaxation, self-compassion, subjective well-being, and self-efficacy, (3) *Mental health* parameters were also self-assessed by the participants, and are comprised of subjective information on various aspects of mental risk factors or illnesses, specifically affect, anxiety, burnout, depression, psychological inflexibility, sleep quality/impairment and subjective mental health, (4) *Stress* is represented by the parameter perceived stress, which is self-assessed though various questionnaires, (5) the parameter *Resilience* is also measured by different questionnaires, (6) *Physical health* parameters include both objective physiological factors that were taken by the study team and measure participants’ blood pressure, heart rate and heart rate variability (HRV), as well as subjective parameters including pain, and subjective physical health, (7) *Work-related factors* are outcomes that are directly related to the work context and include work engagement, absenteeism and productivity; these factors were also self-assessed in the studies included in the present analysis.

A detailed description of the subcategories (Table S3.1), including a list of instruments used and their assignment to outcome categories (Table S3.2), can be found in Supplementary file S3. The process of assigning instruments to (sub-)categories involved subjective assessments by the study team. We aimed to assign outcomes to similar categories as it was previously done in systematic reviews on mindfulness interventions in the workplace. However, previous reviews have not been fully consistent in assigning outcomes to categories. For example, Vonderlin et al. (2020) have assigned the parameters affect and relaxation to the category “well-being and life satisfaction”, and Lomas et al. (2019) have assigned life satisfaction, positive affect and resilience to the category “positive well-being”. As there is no uniform approach in the literature as to how psychological and subjective parameters causally relate to each other, an attempt was made to classify the parameters examined in as much detail as possible and in a way that does justice to the working context, while at the same time allowing for comparison to previous studies.

### Risk of Bias Assessment

The Cochrane Collaboration’s risk-of-bias (RoB) tool 2.0 for RCTs was used to assess the potential for selection bias, performing bias, detection bias, attrition bias, reporting bias, and overall bias in the results of the studies analyzed. We included information from study protocols where this information was available. Risk of bias for each study was assessed for the subjective outcomes (such as stress, burnout, and mindfulness). Separate assessments for the objective physiological parameters (blood pressure, heart rate, HRV) were not carried out due to the predominant focus on subjective parameters within the included studies. This has been done similarly in previous reviews, for example in Vonderlin et al. (2020).

### Synthesis Methods

The statistical software R was used to conduct the meta-analyses. We calculated Hedges’ *g* using post-intervention measures (Borenstein et al., 2009). If several instruments or subcategories were reported for one outcome category (e.g., all three subscales of the Maslach Burnout Inventory), we calculated average mean effects (e.g., total burnout) with appropriate standard errors according to Borenstein et al. (2009) using the agg-Command from package Mad (Version 0.8–2.1). We calculated absolute values of all *SMDs* after checking for negative results, which we did not detect. The values determined for Hedges’ *g* were interpreted according to the guidance by Cohen (1988), where |*g*|= 0.20–0.49 is a small effect, |*g*|= 0.50–0.79 is a medium size effect, and |*g*|≥ 0.80 is a large effect. We calculated average *SMDs* per outcome, averaged over outcome category using random-effects models with the restricted maximum likelihood method using the R package meta (version 4.18–0) and provided Forest plots including prediction intervals. Random-effects models were chosen to account for heterogeneity in the data, which is indicated in the results by *I*^2^ and *τ*^2^. The Hartung-Knapp-Sidik-Jonkman method was used to estimate the confidence interval of the average *SMD*. The aforementioned analyses were conducted for the data referring to post-intervention, short-term follow-up, and long-term follow-up. Linear mixed-effects models were estimated for six of the seven outcome categories based on post-intervention effects using the metafor package (version 2.4–0). There was insufficient data for the outcome “resilience”. These regressions include independent variables for all intervention and population characteristics with less than ten percent of missing data. These are the tri-/dichotomous variables *type of control group* (active vs. passive vs. wait-list), *homework* (yes vs. encouraged/no), *in-class* (yes vs. no), *online* (yes vs. no), *one-on-one* (yes vs. no), *additional material* (yes vs. no), the continuous variables *weeks* (= duration of intervention), *share of female participants*, and *mean age of participants*, as well as a factor variable *intervention_s*, which encompasses eight values according to the eight types of interventions as described in the “Results” section.

### Sensitivity Analysis

Due to notable outliers among effect sizes in almost all outcome categories, we conducted sensitivity analyses in order to check for more plausible overall *SMD* estimates. For each model, influence analysis was conducted using the dmetar package (version 0.0.9000), which automatically provides leave-one-out analysis as well as influence and Baujat diagnostics as described in Viechtbauer and Cheung (2010). Studies that indicated to distort the average effect size due to strong heterogeneity (based on DIFFITS, Cook’s distance, and the covariance ratio) were excluded in a second set of average effect size calculations of each model using the find.outliers command.

### Reporting Bias Assessment

Reporting bias is assessed by funnel plots including Egger’s test (Egger et al., 1997) and *p*-curves (Simonsohn et al., 2014) also using the dmetar package.

## Results

### Study Selection

**Fig. 1:**
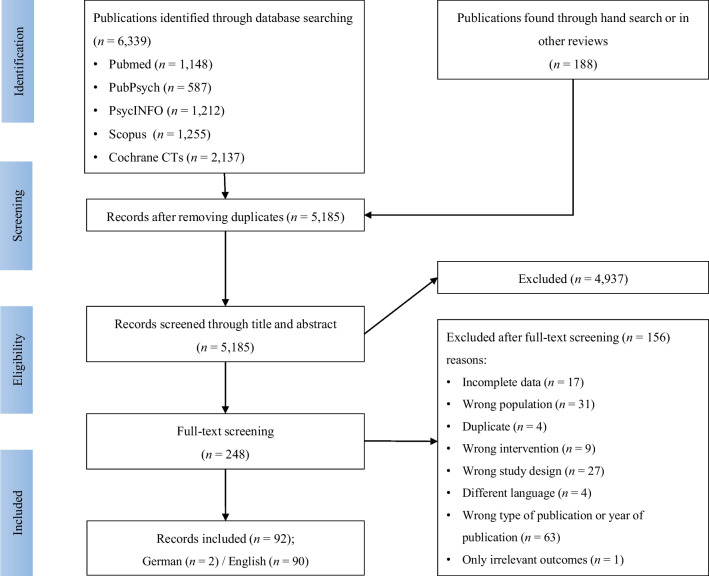
PRISMA flow chart

In total, 248 full-texts were read and reviewed, with 92 publications meeting qualification criteria to be included in this present systematic review. We had to exclude a large number of studies due to not meeting the predefined inclusion criteria: for example, year of publication was outside of defined publication range (mainly incorrectly indicated in databases), studies’ research design did not align with preferred study design (mostly study design was not indicated in abstracts) and missing data that could not be retrieved after contacting authors (see PRISMA chart in Fig. 1). One study was excluded, as it contained only outcomes that had been analyzed by less than three other studies. A high interrater reliability (Cohen’s kappa) of *κ* = 0.98 was achieved based on full-text screening. Some publications contain multiple studies examining different mindfulness interventions. Other studies compare several relevant intervention groups (for example, MBSR vs. *yoga* vs. passive control group). In these cases, the different study arms were listed and analyzed as separate studies. Thereby, control group sizes were divided by the number of active interventions in order to not inflate standard errors (Rücker et al., 2017). In a few cases, the mindfulness intervention acted as a control group. In these cases, the terms control and intervention group were reversed. If studies reported the results of the same mindfulness intervention for different target groups (for example, employees with high and low stress levels, or men and women), we combined the results and calculated standard errors as suggested in Higgins et al. (2021). In total, the evaluation comprises 91 intervention arms (= studies) from 92 published articles. A list of studies excluded after full-text screening can be found in Supplementary file S4.

### Study Characteristics

The mindfulness interventions evaluated in the identified studies were divided into eight different intervention types, each of which can be assigned to either mindfulness-informed or mindfulness-based programs. The latter include MBSR courses, modified MBSR courses, meditation-only courses, and other mindfulness-based programs. Mindfulness-informed interventions include breathing training, acceptance and commitment therapy (ACT)-based courses, movement-oriented programs (*yoga* and *qigong*), and multimodal programs. The latter include, for example, communication, nutrition, and other exercises in addition to mindfulness training.

A total of 9375 working adults (4927 in intervention arms) participated in the included studies. The average intervention group size at the beginning of the studies (T0) was 54 participants, while the control groups consisted of 49 participants, on average. A total of 28 (31%) of the control groups were active control groups. The members of the active control group received another, typically “lighter”, intervention, for example, a flyer about health promotion options at the workplace. A total of 51 control groups were wait-list control groups, and in the other 12 studies, the control groups were passive; i.e., they did not receive any intervention. Average duration of interventions was 9.5 weeks. All included studies and their characteristics are listed in Table 1.

**Table 1:** Characteristics of studies included in quantitative synthesis

Authors	Intervention name	Intervention type*	Control group	Timing (T1, T2, T3, T4)**	Nb. of participants at T0 (IG/CG)^i^	Type of delivery	In-class	Online	One-on-one	Additional material	Homework	Country	Mean age	Share female participants (%)	Outcome domains
Aikens et al. (2014)	Dow Mindful Resilience Program	other mindfulness-based program (mb)	wait-list	7	44/45	digital	Yes	Yes	No	Yes	Yes	USA	na	na	Subj. physical health, Stress, Subj. mental health, Mindfulness, Resilience
Alexander et al. (2015)	Yoga intervention	movement-oriented program (mi)	passive	8	20/20	analogue	Yes	No	No	Yes	Yes	USA	46.38	97.5	Mindfulness, Burnout
Allexandre et al. (2016)—1	Mindfulness stress management program “Stress Free Now”	other mindfulness-based program (mb)	wait-list	8, 8	54/12.3	digital	No	Yes	No	Yes	Encouraged	USA	40.00	83.2	Subj. physical health, Stress, Subj. mental health, Mindfulness, Burnout, Productivity
Allexandre et al. (2016) – 2	Mindfulness stress management program "Stress Free Now" + group support	other mindfulness-based program (mb)	wait-list	8, 8	37/12.3	digital	no	yes	no	yes	encouraged	USA	40.00	83.2	Subj. physical health, Stress, Subj. mental health, Mindfulness, Burnout, Productivity
Allexandre et al. (2016)—3	Mindfulness stress management program “Stress Free Now” + group support + clinical expert	other mindfulness-based program (mb)	wait-list	8, 8	33/12.3	digital	No	Yes	No	Yes	Encouraged	USA	40.00	83.2	Subj. physical health, Stress, Subj. mental health, Mindfulness, Burnout, Productivity
Amutio et al. (2015a, b)	MBSR	MBSR (mb)	wait-list	8	21/21	analogue	Yes	No	No	Yes	Yes	Spain	47.31	57.1	Mindfulness, Relaxation
Arredondo et al. (2017)	M-PBI: a mindfulness program with brief integrated practices	other mindfulness-based program (mb)	wait-list	8, 12	21/19	analogue	Yes	No	No	No	Yes	Spain	36.60	77.5	Stress, Mindfulness, Burnout, Self-compassion
Baby et al. (2019)	Mindfulness program	other mindfulness-based program (mb)	active	4, 12, 24	63/64	analogue	Yes	No	No	Yes	No	New Zealand	na	78.0	Stress
Baccarani et al. (2013)	Zen meditation course	meditation (mb)	wait-list	4	10/10	analogue	Yes	No	No	No	Yes	Italy	na	75.0	Subj. physical health, Subj. mental health, Anxiety, Depression
Bartlett et al. (2017)	Mindfulness at Work Program (MaWP)	other mindfulness-based program (mb)	active	6	20/100	analogue	Yes	No	No	No	Yes	Tasmania/Australia	na	82.5	Stress, Mindfulness, Productivity, Sleep, Job satisfaction, Life satisfaction, Absenteeism
Bhandari et al. (2010)	Yoga Intervention	movement-oriented program (mi)	passive	4	50/50	analogue	Yes	No	No	No	No	India	51.00	50.0	Stress
Bhandari (2017)	Integrated Yogic Intervention	movement-oriented program (mi)	passive	6.4	81/79	analogue	Yes	No	No	No	Encouraged	India	30.78	20.0	Stress
Bostock et al. (2019)	Headspace app	meditation (mb)	wait-list	10	128/110	digital	No	Yes	No	No	No	UK	35.50	59.2	Anxiety, Depression, BP, Affect, Well-being
Brinkborg et al. (2011)	Brief stress management intervention based on ACT	ACT-based program (mi)	wait-list	12	70/36	analogue	Yes	No	No	No	Yes	Sweden	44.00	89.0	Subj. physical health, Stress, Burnout, Psychological inflexibility, Self-efficacy
Calder Calisi (2017)	Relaxation response (RR) technique	breathing program (mi)	wait-list	8	24/22	analogue	Yes	No	No	No	Yes	USA	na	100.0	Stress, Anxiety, Depression, Well-being
Cheema et al. (2013)	Hatha yoga program	movement-oriented program (mi)	passive	10	18/19	analogue	Yes	No	No	No	No	Australia	38.00	81.1	Subj. physical health, Subj. mental health, Anxiety, Job satisfaction, HR, HRV
Chin et al. (2019); Slutsky et al. (2019)	Mindfulness training	other mindfulness-based program (mb)	active	8	31/29	analogue	Yes	No	Yes	Yes	No	USA	30.52	66.7	Stress, Job satisfaction, Affect
Christopher et al. (2018)	Mindfulness-Based Resilience Training (MBRT)	MBSR modified (mb)	wait-list	8, 12	31/30	analogue	Yes	No	No	Yes	Encouraged	USA	43.99	11.5	Stress, Subj. mental health, Mindfulness, Resilience, Burnout, Self-compassion, Anxiety, Depression, Psychological inflexibility
Coelhoso et al. (2019)	Mobile app based on relaxation training, breathing techniques, guided meditation, and positive psychology principles for women’s mental health	multimodal program (mi)	active	8	250/240	digital	No	Yes	No	Yes	na	Brazil	34.60	100.0	Stress, Job satisfaction, Well-being
Cook et al. (2017)	ACHIEVER Resilience Curriculum (ARC)	multimodal program (mi)	active	6	22/22	digital	Yes	Yes	No	Yes	Encouraged	USA	na	na	Stress, Job satisfaction, Self-efficacy
Crain et al. (2017)	Workplace mindfulness training (WMT)	other mindfulness-based program (mb)	wait-list	8, 12	54/59	analogue	Yes	No	No	No	Yes	Canada/USA	46.90	89.0	Mindfulness, Sleep, Job satisfaction, Life satisfaction, Affect
Dahl (2019); Dahl and Dlugosch (2020)	Better Life!-Seminar	multimodal program (mi)	wait-list	6, 6	41/43	analogue	Yes	No	No	No	No	Germany	na	90.5	Stress, Mindfulness, Burnout
Duchemin et al. (2015); Steinberg et al. (2016)	Mindfulness-based intervention with yoga, music, meditation, mindfulness	multimodal program (mi)	wait-list	8	16/16	analogue	Yes	No	No	No	Yes	USA	44.20	87.5	Life satisfaction, Absenteeism
Dwivedi et al. (2015); Dwivedi et al. (2016)	Yoga module	movement-oriented program (mi)	active	10	80/80	analogue	Yes	No	No	No	No	India	27.75	45.0	Affect
Elder et al. (2014)	Transcendental Meditation program	meditation (mb)	wait-list	16	20/20	analogue	Yes	No	Yes	No	Yes	USA	36.14	52.0	Stress, Burnout, Depression
Fang and Li (2015)	Yoga	movement-oriented program (mi)	passive	26	61/59	analogue	Yes	No	No	No	No	China	35.59	100.0	Sleep
Flaxman and Bond (2010)	Worksite stress management training based on ACT	ACT-based program (mi)	wait-list	26	177/134	analogue	Yes	No	No	No	No	UK	41.00	na	Subj. physical health
Flook et al. (2013)	Modified MBSR—adapted specifically for teachers	MBSR modified (mb)	wait-list	11	10/8	analogue	Yes	No	No	No	Yes	USA	43.06	88.9	Subj. physical health, Mindfulness, Burnout, Self-compassion, Affect
Franco et al. (2010)	Flow Meditation Program	meditation (mb)	active	10, 16	34/34	analogue	Yes	No	No	No	Yes	Spain	40.20	57.4	Stress, Subj. mental health, Anxiety, Depression, Pain
Grégoire and Lachance (2015)	Brief Mindfulness-Based Intervention (MBI)	other mindfulness-based program (mb)	wait-list	5	26/15	analogue	Yes	No	No	Yes	Encouraged	Canada	36.10	58.5	Stress, Mindfulness, Burnout, Affect, Well-being
Grégoire et al. (2015)	Brief Mindfulness-Based Intervention (MBI)	other mindfulness-based program (mb)	wait-list	5	24/25	analogue	Yes	No	No	Yes	Encouraged	Canada	35.80	91.0	Stress, Mindfulness, Affect
Hartfiel et al. (2011)	DruYoga intervention	movement-oriented program (mi)	wait-list	6	24/24	analogue	Yes	Yes	No	No	Encouraged	UK	39.30	90.0	Self-efficacy
Hartfiel et al. (2012)	DruYoga intervention	movement-oriented program (mi)	wait-list	8	37/37	analogue	Yes	Yes	No	No	Yes	UK	44.80	90.0	Stress, Affect, Pain
Hartfiel et al. (2017)	DruYoga intervention	movement-oriented program (mi)	active	8, 16	76/75	analogue	Yes	Yes	No	No	Yes	UK	43.86	93.0	Subj. physical health, Pain
Huang et al. (2015)	Shorter Mindfulness-Based Stress Reduction (MBSR)	MBSR modified (mb)	wait-list	8, 4, 8	72/72	analogue	Yes	Yes	No	No	Yes	Taiwan/China	42.55	41.0	Subj. physical health, Stress
Hülsheger et al. (2013)	Self-training intervention that builds upon MBCT and MBSR	MBSR modified (mb)	active	2	102/101	analogue	No	No	No	Yes	No	Germany	38.60	71.9	Mindfulness, Burnout, Job satisfaction
Hülsheger et al. (2015)	Self-training intervention that builds upon MBCT and MBSR	MBSR modified (mb)	wait-list	2	75/73	analogue	No	No	No	Yes	No	Germany	37.00	69.3	Mindfulness, Relaxation, Sleep
Ireland et al. (2017)	Mindfulness training program	other mindfulness-based program (mb)	active	10	23/21	analogue	Yes	No	No	No	Encouraged	Australia	26.88	64.0	Stress, Burnout
Jennings et al. (2017)	Cultivating Awareness and Resilience in Education (CARE for Teachers)	multimodal program (mi)	wait-list	12	118/106	analogue	Yes	No	No	Yes	Yes	USA	41.50	93.0	Subj. physical health, Stress, Mindfulness,
Klatt et al. (2009)	Low-dose work-site MBSR intervention (MBSR-ld)	MBSR modified (mb)	wait-list	6	24/24	analogue	Yes	No	No	Yes	Yes	USA	44.96	75.0	Stress, Mindfulness, Sleep
Klatt et al. (2017)	Mindfulness in Motion (MIM)	multimodal program (mi)	wait-list	8	41/40	analogue	Yes	No	No	No	Yes	Denmark	42.91	68.5	Stress, Sleep, Work-engagement
Krick and Felfe (2020)	Mindfulness and resource-based worksite training	multimodal program (mi)	passive	6	126/141	analogue	Yes	No	No	No	Yes	Germany	25.96	21.3	Subj. physical health, Mindfulness, Affect, HR, HRV
Lacerda et al. (2018)	PROGRESS mindfulness program	other mindfulness-based program (mb)	wait-list	8	39/38	analogue	Yes	No	No	No	No	Brazil	36.60	54.5	Stress, Subj. mental health, Mindfulness, Anxiety, Depression
Lebares et al. (2019)	Modified Mindfulness-based Stress Reduction (modMBSR)	MBSR modified (mb)	active	14, 38	12/9	analogue	Yes	No	No	No	Yes	USA	28.31	38.1	Subj. physical health, Stress, Mindfulness, Resilience, Burnout
Lemaire et al. (2011)	Biofeedback-based stress management tool	multimodal program (mi)	wait-list	4	21/19	analogue	No	No	Yes	Yes	No	Canada	46.23	42.5	Stress, BP, HR
Lilly et al. (2019)	Online mindfulness-based intervention	other mindfulness-based program (mb)	wait-list	7, 12	163/160	digital	No	Yes	No	Yes	Yes	Canada/USA	na	81.9	Stress, Mindfulness
S.-L. Lin et al. (2015)	Yoga program	movement-oriented program (mi)	active	12	30/30	analogue	Yes	No	No	No	No	Taiwan/China	30.92	80.0	Stress
L. Lin et al. (2019)	Modified MBSR program	MBSR modified (mb)	wait-list	8, 12	55/55	analogue	Yes	No	No	Yes	Yes	China	31.53	93.3	Stress, Resilience, Job satisfaction, Affect
Ludwigs et al. (2019)	Trivago flowlab	multimodal program (mi)	passive	7	130/123	analogue	Yes	No	No	Yes	Encouraged	Germany	na	na	Mindfulness, Productivity, Sleep, Job satisfaction, Life satisfaction, Work engagement
Mackenzie et al. (2006)	Shortened MBSR program	MBSR modified (mb)	wait-list	4	16/14	analogue	Yes	No	No	No	Yes	Canada	46.57	96.7	Burnout, Relaxation, Job satisfaction, Life satisfaction
Maddux et al. (2018)	Power yoga intervention	movement-oriented program (mi)	wait-list	8	45/45	analogue	Yes	No	No	No	No	Sweden	46.00	82.5	Subj. physical health, Stress, Mindfulness, Anxiety, Depression, Sleep, Life satisfaction, Psychological inflexibility
Manotas et al. (2014)	Mindfulness intervention	other mindfulness-based program (mb)	wait-list	4	66/65	analogue	Yes	No	No	No	Yes	Colombia	39.05	90.3	Stress, Subj. mental health, Mindfulness, Anxiety, Depression, Pain
Masih et al. (2020)	RELAX: Progressive muscle relaxation and mindfulness meditation	multimodal Program (mi)	wait-list	8	19/17	analogue	Yes	No	No	No	Yes	Australia	36.17	66.7	Stress, Mindfulness, Anxiety
McConachie et al. (2014)	Acceptance and Mindfulness Workshop	ACT-based program (mi)	wait-list	6, 6	66/54	analogue	Yes	No	No	No	Yes	UK/Scotland	43.00	74.2	Subj. physical health, Stress, Well-being, Psychological inflexibility
Michel et al. (2014); Rexroth et al. (2017)	Mindfulness as a cognitive–emotional segmentation strategy	multimodal program (mi)	wait-list	3, 2	208/204	digital	No	Yes	No	Yes	Yes	Germany	41.41	71.1	Mindfulness, Burnout, Relaxation, Job satisfaction, Life satisfaction, Affect, Self-efficacy
Mino et al. (2006)	Stress-management program based on the cognitive behavioral approach	other mindfulness-based program (mb)	passive	12	28/30	analogue	Yes	No	No	Yes	Encouraged	Japan	38.00	0.0	Subj. physical health, Stress, Depression, Job satisfaction
Mistretta et al. (2018)	Mindfulness-based resilience training (MBRT) program	MBSR modified (mb)	active	6, 12	22/15	analogue	Yes	No	No	No	Yes	USA	47.35	91.9	Stress, Burnout, Self-compassion, Anxiety, Depression, Well-being
Molek-Winiarska and Żołnierczyk-Zreda (2018)	MBSR	MBSR (mb)	passive	36	32/34	analogue	Yes	No	No	No	Yes	Poland	40.41	0.0	Subj. physical health, Anxiety, Depression
Möltner et al. (2018)	App-based mindfulness-training “7mind”	meditation (mb)	wait-list	2	146/160	digital	No	Yes	No	No	Yes	Germany	42.86	69.0	Mindfulness, Burnout, Job satisfaction, Self-efficacy, Work engagement
Nübold et al. (2019)	App-based mindfulness training “Headspace”	meditation (mb)	wait-list	4	93/80	digital	No	Yes	No	No	No	International	41.24	40.1	Mindfulness
O’Brien et al. (2019)	Acceptance and commitment therapy (ACT)	ACT-based program (mi)	wait-list	5	37/34	analogue	Yes	No	No	No	No	USA	37.91	86.0	Subj. mental health, Absenteeism, Pain
Pandya (2019)	Smartphone meditation app (M-App)	meditation (mb)	active	52	48/48	digital	No	Yes	No	No	No	India, Thailand, South Africa, Kenya	42.69	38.5	Resilience, Burnout
Pang and Ruch (2019)—1	MBSR	MBSR (mb)	wait-list	9, 3, 11, 23	21/10.5	analogue	Yes	No	No	No	Encouraged	Switzerland	44.20	68.3	Stress, Job satisfaction, Well-being
Pang and Ruch (2019)—2	Mindfulness-Based Strengths Practice (MBSP)	multimodal program (mi)	wait-list	9, 3, 11, 23	21/10.5	analogue	Yes	No	No	No	Encouraged	Switzerland	44.20	68.3	Stress, Job satisfaction, Well-being
Pipe et al. (2009)	Brief mindfulness meditation course (MMC)	MBSR modified (mb)	active	4	17/17	analogue	Yes	No	No	No	Yes	USA	49.80	96.9	Subj. physical health, Stress, Subj. mental health, Anxiety, Depression, Self-efficacy, Pain
Querstret et al. (2017)	Internet-based instructor-led mindfulness intervention	MBSR modified (mb)	wait-list	7, 12, 24	60/58	digital	No	Yes	No	Yes	Yes	UK	40.68	80.5	Mindfulness, Sleep
Rao et al. (2017)	Mind Sound Resonance Technique (Yogic relaxation)	multimodal program (mi)	passive	4	30/30	analogue	Yes	No	No	No	No	India	41.50	100.0	Subj. physical health, Stress, Anxiety, Sleep, Self-efficacy
Riley et al. (2017)	Yoga-based stress management	movement-oriented program (mi)	active	8, 8, 24	19/19	analogue	Yes	No	No	No	No	USA	44.60	84.2	Subj. physical health, Stress, Subj. mental health, Burnout, Relaxation, Anxiety, Depression, Sleep, BP, HR
Roeser et al. (2013)	Mindfulness training	multimodal program (mi)	wait-list	8, 12	60/59	analogue	Yes	No	No	No	Yes	Canada/USA	46.90	89.0	Stress, Mindfulness, Burnout, Self-compassion, BP, HR
Sakuma et al. (2012)	Brief, simple, home-based yoga program	movement-oriented program (mi)	passive	2, 2	67/31	analogue	No	No	No	Yes	Encouraged	Japan	33.61	100.0	Pain
Schroeder et al. (2018)	Mindful Medicine Curriculum (MMC)	MBSR modified (mb)	wait-list	1, 12	16/17	analogue	Yes	No	No	No	Encouraged	USA	42.76	73.0	Stress, Mindfulness, Resilience, Burnout
Shonin et al. (2014)	Meditation Awareness Training (MAT)	meditation (mb)	active	8, 12	76/76	analogue	Yes	No	Yes	No	Encouraged	UK	40.03	56.9	Stress, Depression, Job satisfaction
Singh et al. (2016)	Integrated mindfulness-based training with positive behavoir support	multimodal program (mi)	active	40	39/38	analogue	Yes	No	No	No	Yes	USA	44.05	68.0	Stress
Singh et al. (2020)	Integrated mindfulness-based training with positive behavoir support	multimodal program (mi)	active	40	60/63	analogue	Yes	No	No	No	Yes	USA	43.43	70.7	Stress, Burnout
Smith et al. (2020)	Wearable-based stress management intervention	other mindfulness-based program (mb)	wait-list	4	107/108	digital	No	Yes	No	No	Encouraged	USA	33.20	55.0	Stress
Sood et al. (2011)	Stress Management and Resiliency Training (SMART)	multimodal program (mi)	wait-list	8	20/20	analogue	No	No	Yes	No	Yes	USA	48.50	47.5	Stress, Resilience, Anxiety, Life satisfaction
Sood et al. (2014)	Stress Management and Resiliency Training (SMART)	multimodal program (mi)	wait-list	12	13/13	analogue	Yes	No	Yes	No	Yes	USA	47.75	42.3	Stress, Mindfulness, Resilience, Anxiety, Life satisfaction
Sutarto et al. (2012)	Resonant Breathing Biofeedback Training	breathing program (mi)	active	5	20/20	analogue	Yes	No	No	No	Yes	Malaysia	36.30	100.0	Stress, Anxiety, Depression, HRV
Tahamsebi et al. (2018)	Mindfulness Training	multimodal program (mi)	passive	7	15/15	analogue	Yes	No	No	No	No	Iran	38.00	100.0	Depression, Job satisfaction, Life satisfaction, Affect
Taylor et al. (2016)	Stress Management and Relaxation Training (SMART)	multimodal program (mi)	wait-list	9, 16	26/30	analogue	Yes	No	No	No	Encouraged	Canada	47.00	89.8	Stress
Telles et al. (2012)—1	Brief yoga intervention	movement-oriented program (mi)	active	0.1	70/10	analogue	Yes	No	No	No	No	India	30.30	0.0	Anxiety
Telles et al. (2012)—2	Breath awareness	meditation (mb)	active	0.1	70/10	analogue	Yes	No	No	No	No	India	30.30	0.0	Anxiety
Valley & Stallones (2017)	MBSR	MBSR (mb)	wait-list	8	11/12	analogue	Yes	No	No	No	Yes	USA	na	91.0	Mindfulness
Van Berkel et al. (2014); Van Dongen et al. (2016)	Mindful Vitality In Practice (VIP) intervention	multimodal program (mi)	active	26, 26	129/128	analogue	Yes	No	Yes	Yes	Yes	Netherlands	45.55	67.4	Subj. mental health, Mindfulness, Relaxation, Work engagement
Versluis et al. (2018)	Smartphone application MovisensXS with emotion registration and mindfulness program	multimodal program (mi)	wait-list	4	41/38	digital	No	Yes	No	No	Yes	Netherlands	43.23	71.0	Subj. physical health, Stress, Mindfulness, Anxiety, Affect, HR, HRV
Watanabe et al. (2019)	Brief mindfulness-based stress management program	MBSR modified (mb)	active	13, 13, 39	40/40	analogue	No	No	Yes	No	Encouraged	Japan	30.10	100.0	Subj. physical health, Burnout, Productivity, Anxiety, Depression, Sleep
Wolever et al. (2012)—1	Viniyoga Stress Reduction Program	movement-oriented program (mi)	active	14	90/17.7	analogue	Yes	No	No	No	Encouraged	USA	42.01	76.3	Stress, Mindfulness, Depression, Sleep, Job satisfaction, BP, HRV, Pain
Wolever et al. (2012)—2	Mindfulness at Work: online	other mindfulness-based program (mb)	active	14	52/17.7	digital	Yes	Yes	No	No	Encouraged	USA	43.49	79.0	Stress, Mindfulness, Depression, Sleep, Job satisfaction, BP, HRV, Pain
Wolever et al. (2012)—3	Mindfulness at Work: in-class	other mindfulness-based program (mb)	active	14	44/17.7	analogue	Yes	No	No	No	Encouraged	USA	43.43	79.5	Stress, Mindfulness, Depression, Sleep, Job satisfaction, BP, HRV, Pain
Yang et al. (2018)	Modified MBSR program	MBSR modified (mb)	active	8	50/50	analogue	Yes	No	No	No	Encouraged	China	29.50	68.0	Subj. physical health, Stress, Subj. mental health, Anxiety, Depression
Żołnierczyk-Zreda et al. (2016)	MBSR	MBSR (mb)	wait-list	12	78/78	analogue	Yes	No	Yes	No	Yes	Poland	39.40	49.0	Subj. physical health, Stress, Absenteeism, Affect, Self-efficacy

*mb: mindfulness-based intervention; *mi*: mindfulness-informed intervention; **T1: timing of post-intervention data collection in weeks (approximate length of intervention); *T2*–*T4*: post-intervention data collection in weeks since T1

### Risk of Bias

Risk of bias for each study was assessed for the subjective outcomes (such as stress, burnout, and mindfulness). An interrater reliability (Cohen’s kappa) of *κ* = 0.84 was achieved. Supplementary file S5 shows the results of the assessment of the risk of bias for all five bias domains. Risk of bias was generally high, except in two studies, as further explained in the “Discussion” section.

### Average SMD

Table 2 shows the results of the average *SMD* calculations for post-intervention measures. Forest plots can be found in Supplementary file S6. Average *SMD*s were statistically significant for all seven pooled outcome categories. For mindfulness (*k* = 39, *SMD* = 0.43, 95%-*CI* [0.33,0.52], *I*^2^ = 98.8%), well-being (*k* = 35, *SMD* = 0.63, 95%-*CI* [0.34,0.93], *I*^2^ = 98.9%), mental health (*k* = 68, *SMD* = 0.67, 95%-*CI* [0.48,0.86], *I*^2^ = 99.3%), stress (*k* = 59, *SMD* = 0.72, 95%-*CI* [0.54,0.90], *I*^2^ = 98.9%), physical health (*k* = 37, *SMD* = 0.45, 95%-*CI* [0.32,0.59], *I*^2^ = 99.6%), and work-related factors (*k* = 29, *SMD* = 0.62, 95%-*CI* [0.14,1.10], *I*^2^ = 99.1%), we estimated small to medium average effects, which are statistically significant at *p* < 0.001. For resilience, with a small number of studies (*k* = 8, *SMD* = 1.06, 95%-*CI* [− 0.22,2.34], *I*^2^ = 98.8%), we obtained a large average *SMD*, which was, however, hardly statistically significant. The *SMD* results in the subcategories are mixed and can be found in Table 2. Small to medium effect sizes were found for life satisfaction (*k* = 10, *SMD* = 0.40, 95%-*CI* [0.15,0.66], *I*^2^ = 95.5%), self-compassion (*k* = 5, *SMD* = 0.66, 95%-*CI* [0.18,1.13], *I*^2^ = 93.2%), subjective well-being (*k* = 8, *SMD* = 0.51, 95%-*CI* [0.14, 0.89], *I*^2^ = 94.0%), affect (*k* = 14, *SMD* = 0.93, 95%-*CI* [0.31, 1.54], *I*^2^ = 99.6%), anxiety (*k* = 23, *SMD* = 0.53, 95%-*CI* [0.31,0.74, *I*^2^ = 97.8%), burnout (*k* = 24, *SMD* = 0.70, 95%-*CI* [0.33,1.07], *I*^2^ = 99.0%), depression (*k* = 22, *SMD* = 0.51, 95%-*CI* [0.29,0.73], *I*^2^ = 98.7%), sleep (*k* = 15, *SMD* = 0.46, 95%-*CI* [0.22,0.70], *I*^2^ = 98.7%), subjective mental health (*k* = 15, *SMD* = 0.60, 95%-*CI* [0.34,0.85], *I*^2^ = 98.7%), heart rate variability (*k* = 7, *SMD* = 0.46, 95%-*CI* [0.15,0.78], *I*^2^ = 99.0%), pain (*k* = 10, *SMD* = 0.31, 95%-*CI* [0.11,0.52], *I*^2^ = 94.9%), subjective physical health (*k* = 25, *SMD* = 0.40, 95%-*CI* [0.30,0.50], *I*^2^ = 96.2%), job satisfaction (*k* = 20, *SMD* = 0.47, 95%-*CI* [0.25,0.68], *I*^2^ = 98.6%), and productivity (*k* = 6, *SMD* = 0.27, 95%-*CI* [0.18,0.36], *I*^2^ = 40.1%). Smaller or nonsignificant average effect sizes were found for relaxation, self-efficacy, psychological inflexibility, blood pressure, heart rate, absenteeism, and work engagement. The number of these studies, however, has been relatively small (*k* < 9) compared to the numbers of studies that analyzed the outcomes with small to medium significant effect sizes. Notable is a high level of heterogeneity and large prediction intervals for all outcomes except productivity. In addition, Forest plots showed that some studies have extremely large *SMDs*, while others have very small effect sizes. Therefore, the average results should be interpreted with caution. Results of sensitivity analyses and meta-regressions are discussed below.

**Table 2:** Random-effects results: Average *SMD* post-intervention by outcome category

Before influence analysis							After removing influential cases					
Outcome category	*k*	*aSMD*	*CI*	*PI*	*Q*	*I*^2^*/τ*^2^	*k*	*aSMD*	*CI*	*PI*	*Q*	*I*^2^*/τ*^2^
Mindfulness												
Mindfulness	39	0.43***	[0.33; 0.52]	[− 0.11; 0.98]	3103	98.8/0.07	22	0.40***	[0.32; 0.48]	[0.14; 0.66]	60	64.9/0.01
Well-being												
Pooled well-being factors	35	0.63***	[0.34; 0.93]	[− 1.12; 2.39]	3027	98.9/0.72	22	0.54***	[0.42; 0.66]	[0.04; 1.04]	248	91.5/0.05
Life satisfaction	10	0.40**	[0.15; 0.66]	[− 0.42; 1.23]	199	95.5/0.11	9	0.32**	[ 0.16; 0.47]	[− 0.13; 0.77]	117	93.1/0.03
Relaxation	6	0.23	[− 0.02; 0.48]	[− 0.45; 0.91]	159	96.9/0.05	5	0.13	[− 0.01; 0.28]	[− 0.26; 0.53]	103	96.1/0.01
Self-compassion	5	0.66*	[0.18; 1.13]	[− 0.62; 1.93]	59	93.2/0.13	4	0.80**	[0.50; 1.11]	[− 0.04; 1.65]	15	79.5/0.03
Subjective well-being	8	0.51*	[0.14; 0.89]	[− 0.60; 1.62]	116	94.0/0.18	7	0.38*	[0.09; 0.67]	[− 0.35; 1.11]	65	90.8/0.07
Self-efficacy	8	1.18	[− 0.16; 2.53]	[− 2.98; 5.34]	1926	99.6/2.57	7	0.64**	[0.15; 1.13]	[− 0.80; 2.08]	358	98.3/0.27
Mental health												
Pooled mental health factors	68	0.67***	[0.48; 0.86]	[− 0.89; 2.23]	10,305	99.3/0.60	36	0.57***	[0.52; 0.63]	[0.27; 0.88]	559	93.7/0.02
Affect	14	0.93**	[0.31; 1.54]	[− 1.47; 3.33]	3555	99.6/1.13	12	0.68***	[0.48; 0.89]	[0.01; 1.35]	264	95.8/0.08
Anxiety	23	0.53***	[0.31; 0.74]	[− 0.50; 1.55]	1013	97.8/0.23	17	0.42***	[0.31; 0.54]	[0.01; 0.84]	108	85.2/0.03
Burnout	24	0.70***	[0.33; 1.07]	[− 1.15; 2.55]	2409	99.0/0.76	20	0.45***	[0.37; 0.53]	[0.14; 0.76]	197	90.4/0.02
Depression	22	0.51***	[0.29; 0.73]	[− 0.53; 1.55]	1638	98.7/0.24	15	0.49***	[0.34; 0.64]	[− 0.04; 1.03]	108	87.0/0.06
Psychological inflexibility	4	0.16	[− 0.07; 0.39]	[− 0.48; 0.80]	16	81.4/0.02						
Sleep	15	0.46***	[0.22; 0.70]	[− 0.47; 1.40]	1086	98.7/0.18	13	0.33***	[0.26; 0.40]	[0.13; 0.53]	67	82.2/0.01
Subj. mental health	15	0.60***	[0.34; 0.85]	[− 0.41; 1.60]	1078	98.7/0.20	9	0.53***	[0.34; 0.72]	[0.00; 1.05]	39	79.3/0.04
Stress												
Stress	59	0.72***	[0.54; 0.90]	[− 0.66; 2.10]	5406	98.9/0.47	33	0.64***	[0.57; 0.70]	[0.30; 0.97]	338	90.5/0.03
Resilience												
Resilience	8	1.06*	[− 0.22; 2.34]	[− 2.90; 5.02]	573	98.8/2.33	7	0.53**	[ 0.26; 0.80]	[− 0.19; 1.25]	57	89.4/0.07
Physical health												
Pooled physical health factors	37	0.45***	[0.32; 0.59]	[− 0.36; 1.27]	8323	99.6/0.16	25	0.43***	[0.37; 0.50]	[0.15; 0.71]	164	85.4/0.02
Blood pressure	7	0.54	[− 0.20; 1.27]	[− 1.66; 2.73]	5087	99.9/0.64	6	0.22*	[0.04; 0.40]	[− 0.25; 0.70]	34	85.4/0.02
Heart rate	6	0.21**	[0.08; 0.33]	[− 0.11; 0.52]	18	71.8/0.01						
Heart rate variability	7	0.46**	[0.15; 0.78]	[− 0.48; 1.41]	601	99.0/0.12	6	0.36**	[0.12; 0.60]	[− 0.31; 1.02]	64	92.2/0.05
Pain	10	0.31**	[0.11; 0.52]	[− 0.35; 0.98]	177	94.9/0.08	8	0.21**	[0.04; 0.38]	[− 0.29; 0.71]	97	92.8/0.04
Subj. physical health	25	0.40***	[0.30; 0.50]	[− 0.05; 0.85]	624	96.2/0.04	16	0.38***	[0.32; 0.44]	[0.19; 0.57]	62	75.9/0.01
Work-related factors												
Pooled work-related factors	29	0.62*	[0.14; 1.10]	[− 2.00; 3.24]	2969	99.1/1.57	25	0.32***	[0.23; 0.41]	[− 0.09; 0.74]	398	94.0/0.04
Absenteeism	4	1.95	[− 3.32; 7.22]	[− 13.96; 17.86]	1089	99.7/10.94						
Job satisfaction	20	0.47***	[0.25; 0.68]	[− 0.48; 1.42]	1362	98.6/0.19	15	0.34***	[0.26; 0.43]	[0.03; 0.65]	110	87.2/0.02
Productivity	6	0.27***	[0.18; 0.36]	[0.11; 0.43]	8	40.1/0.00						
Work engagement	4	0.18	[− 0.11; 0.47]	[− 0.69; 1.05]	418	99.3/0.03						

*k*: number of studies; *aSMD*: average standardized mean difference (Hedges’ *g*); *CI*: 95% confidence interval; *PI*: prediction interval; ***p < 0.001; **p < 0.01;*p < 0.05. See Supplementary file S5 for information on which studies were excluded as outliers

Less than half of the studies analyzed outcomes between 1 and 12 weeks after the end of the intervention (Table 3). For the pooled categories mindfulness (*k* = 11, *SMD* = 0.53, 95%-CI [0.27,0.80], *I*^2^ = 98.3%), well-being (*k* = 11, *SMD* = 0.44, 95%-CI [0.30,0.58], *I*^2^ = 93.3%), mental health (*k* = 16, *SMD* = 0.56, 95%-CI [0.24,0,87], *I*^2^ = 99.2%), stress (*k* = 17, *SMD* = 0.61, 95%-CI [0.34,0.89], *I*^2^ = 99.1%), and physical health (*k* = 8, *SMD* = 0.43, 95%-CI [0.14,0.72], *I*^2^ = 91.6%), the average *SMD*s are statistically significant and of small to medium size. Small to medium average *SMD*s for the subcategories with at least four studies were found for burnout (*k* = 10, *SMD* = 0.43, 95%-CI [0.22,0.64], *I*^2^ = 93.5%), subjective mental health (*k* = 5, *SMD* = 0.55, 95%-CI [− 0.14,1.24], *I*^2^ = 90.0%), and subjective physical health (*k* = 6, *SMD* = 0.50, 95%-CI [0.07,0.92], *I*^2^ = 92.3%). Nonsignificant effect sizes were obtained for depression, job satisfaction, and pooled work-related factors. Average *SMDs* for long-term follow-ups could only be estimated for a small set of outcomes as the number of studies collecting data after 13 or more weeks was too small (Table 4). Average *SMDs* for pooled well-being factors and subjective physical health turned nonsignificant, while pooled mental health factors (*k* = 4, *SMD* = 0.21, 95%-CI [0.01,0.40], *I*^2^ = 89.4%), stress (*k* = 5, *SMD* = 0.50, 95%-CI [0.09,0.92], *I*^2^ = 92.6%), and pooled physical health factors (*k* = 4, *SMD* = 0.33, 95%-CI [0.13,0.54], *I*^2^ = 67.3%) have been found to have small and significant effect sizes. A high level of heterogeneity is also present here.

**Table 3:** Random-effects results: Average *SMD* short-term follow-up by outcome category

Before influence analysis							After removing influential cases					
Outcome category	*k*	*aSMD*	*CI*	*PI*	*Q*	*I*^2^*/τ*^2^	*k*	*aSMD*	*CI*	*PI*	*Q*	*I*^2^*/τ*^2^
Mindfulness												
Mindfulness	11	0.53**	[0.27; 0.80]	[− 0.37; 1.44]	591	98.3/0.15	7	0.64**	[0.30; 0.98]	[− 0.28; 1.56]	97	93.8/0.11
Well-being												
Pooled well-being factors	11	0.44***	[0.30; 0.58	[− 0.03; 0.91]	150	93.3/0.04	8	0.45	[0.34; 0.55]	[0.15; 0.74]	31	77.4/0.01
Mental health												
Pooled mental health factors	16	0.56**	[0.24; 0.87]	[− 0.73; 1.84]	1865	99.2/0.34	13	0.47***	[0.29; 0.66]	[− 0.16; 1.11]	165	92.7/0.08
Burnout	10	0.43**	[0.22; 0.64]	[− 0.24; 1.09]	138	93.5/0.07	9	0.36**	[0.20; 0.52]	[− 0.11; 0.83]	104	92.3/0.04
Depression	4	0.88	[− 0.75; 2.52]	[− 4.06; 5.82]	665	99.5/1.05						
Subj. mental health	5	0.55*	[− 0.14; 1.24]	[− 1.30; 2.40]	40	90.0/0.28						
Stress												
Stress	17	0.61***	[0.34; 0.89]	[− 0.55; 1.78]	1771	99.1/0.61	10	0.64***	[0.43; 0.84]	[0.04; 1.23]	55	83.7/0.06
Physical health												
Pooled physical health factors	8	0.43**	[0.14; 0.72]	[− 0.37; 1.23]	83	91.6/0.09						
Subj. physical health	6	0.50*	[0.07; 0.92]	[− 0.60; 1.59]	65	92.3/0.13						
Work-related factors												
Pooled work-related factors	9	0.50	[− 0.02; 1.03]	[− 1.18; 2.19]	1478	99.5/0.46	8	0.26***	[0.17; 0.34]	[0.08; 0.43]	14	50.7/0.00
Job satisfaction	6	0.58	[− 0.29; 1.44]	[− 1.88; 3.04]	1474	99.7/0.67	5	0.25**	[0.16; 0.34]	[0.03; 0.47]	10	60.8/0.00

*k*: number of studies; *aSMD*: average standardized mean difference (Hedges’ *g*); *CI*: 95% confidence interval; *PI*: prediction interval; ***p < 0.001; **p < 0.01; *p < 0.05. See Supplementary file S5 for information on which studies were excluded as outliers

**Table 4:** Random-effects results: Average *SMD* long-term follow-up by outcome category

Before influence analysis						
Outcome category	*k*	*aSMD*	*CI*	*PI*	*Q*	*I*^2^*/τ*^2^
Well-being						
Pooled well-being factors	4	0.39	[− 0.08; 0.87]	[− 0.98; 1.77]	21	85.9/0.08
Mental health						
Pooled mental health factors	4	0.21*	[0.01; 0.40]	[− 0.33; 0.73]	28	89.4/0.01
Stress						
Stress	5	0.50*	[0.09; 0.92]	[− 0.59; 1.56]	54	92.6/0.10
Physical health						
Pooled physical health factors	4	0.33*	[0.13; 0.54]	[− 0.19; 0.85]	9	67.3/0.01
Subj. physical health	4	0.42	[− 0.00; 0.84]	[− 0.70; 1.54]	22	86.6/0.05
Work-related factors						
Pooled work-related factors	4	0.18*	[0.03; 0.32]	[− 0.18; 0.54]	9	65.4/0.00

*k*: number of studies; *aSMD*: average standardized mean difference (Hedges’ *g*); *CI*: 95% confidence interval; *PI*: prediction interval; ***p < 0.001; **p < 0.01; *p < 0.05. See Supplementary file S5 for information on which studies were excluded as outliers

### Sensitivity Analysis

Several studies included in our analysis show extreme outliers, with *SMD* values exceeding 4 (individual effect sizes are displayed in Supplementary file S6). In all cases, these large *SMD* values are due to significant differences between treatment and control groups at baseline. Interestingly, these outliers are not limited to small-case studies, but also occur in larger-scale studies, such as Pandya (2019) and Żołnierczyk-Zreda et al. (2016), who each included more than 90 participants in their studies. Because we identified only small variations in risk of bias assessment results, controlling for risk of bias in our estimations of average *SMDs* was not feasible. However, we performed sensitivity analyses without outliers detected through influence analyses and Baujat plots (Supplementary file S6) for all outcomes. This resulted in smaller *SMDs* for all pooled outcomes at post-intervention as seen in Tables 2 and 3. For the pooled outcomes mindfulness, well-being, mental health, stress and physical health, average *SMDs* dropped by 10–20% in size and remained significant; resilience (*k* = 7, *SMD* = 0.53, 95%-CI [0.26,0.80], *I*^2^ = 89.4%) turned significant. The average *SMD* of pooled work-related factors dropped by 50%, yet the effect remained small and significant. The average effect sizes of almost all subcategories remained similar in magnitude after removing outliers, and increased for self-compassion (*k* = 4, *SMD* = 0.80, 95%-CI [0.50,1.11], *I*^2^ = 79.5%), and turned significant for self-efficacy (*k* = 7, *SMD* = 0.64, 95%-CI [0.15,1.13], *I*^2^ = 98.3%) as well as for blood pressure (*k* = 6, *SMD* = 0.22, 95%-CI [0.04,0.40], *I*^2^ = 85.4%). Heterogeneity indicators are reduced at least slightly in all cases. Adjusted short-term follow-up effects were relatively similar to post-intervention effects. Only the average *SMD* of pooled well-being was rendered nonsignificant, while average *SMDs* for job satisfaction (*k* = 5, *SMD* = 0.25, 95%-CI [0.16,0.34], *I*^2^ = 60.8%) and work-related factors in general (*k* = 8, *SMD* = 0.26, 95%-CI [0.17,0.34], *I*^2^ = 50.7%) turned significant albeit small. There were no outliers in long-term follow-up studies.

### Reporting Bias

Based on Egger’s tests and *p*-curves, we did not detect reporting bias, except for the category of well-being outcomes at post-intervention and long-term follow-up. These specific results can also be found in Supplementary file S6.

### Meta-Regression Results

In total, six meta-regressions were run including the moderators for which there were less than 5% missing values (Table 5). For mindfulness, the moderators explained no amount of heterogeneity in effects sizes (*R*^2^ = 0.00), while for well-being, mental health, stress, physical health, and work-related factors, 47.77%, 43.14%, 14.75%, 44.45%, and 4.49% could be explained by the included moderators. The regressions show that MBSR interventions are more effective than ACT-related interventions, modified MBSR-courses, other mindfulness-based interventions, and multimodal interventions for well-being (*b* = 1.9, *p* < 0.001), and more effective than all other types of interventions for mental health (*b* = 3.23, *p* < 0.001). For physical health outcomes, MBSR-interventions are slightly more effective than ACT-related interventions (*b* = 0.37, *p* < 0.05) but not more effective than other types. Instead, meditation courses are more effective than all other interventions in improving physical health (*b* = 1.21, *p* < 0.001), except breathing interventions. Meditation interventions are relatively more effective than ACT-based interventions for mental health outcomes (*b* = 0.91, *p* < 0.01), and multimodal interventions are relatively more effective than ACT-based interventions for mental health outcomes (*b* = 0.88, *p* < 0.01). While these results point to an overall greater effectiveness of mindfulness-based interventions over mindfulness-informed interventions, a separate analysis where these two groups of interventions were compared against each other, did not reveal such evidence. Instead, based on these findings, it can be concluded that certain types of interventions seem more effective for achieving some outcomes than others, but there is no systematic superiority by either mindfulness-based or mindfulness-informed interventions. Only MBSR courses seem to be more effective when compared to other types of interventions.

**Table 5:** Meta-regression results post-intervention

Mixed-effects model												
Outcome categories	Mindfulness (*k* = 33)		Well-being (*k* = 30)		Mental health (*k* = 61)		Stress (*k* = 51)		Physical health (*k* = 34)		Work-related factors (*k* = 25)	
*Dependent var*	*b*	*se*	*b*	*se*	*b*	*se*	*b*	*se*	*b*	*se*	*b*	*se*
Intercept	0.55	0.58	4.50	2.75	0.0073	0.63	0.76	1.17	0.45	0.63	1.16	5.10
Intervention type												
ACT modified	/	*/*	*Base*	*Base*	*Base*	*Base*	*Base*	*Base*	*Base*	*Base*	*Base*	*Base*
Breathing	*/*	*/*	/	/	− 0.10	0.64	− 0.87	0.89	0.71	0.41	/	/
MBSR	0.12	0.39	1.90***	0.68	3.23***	0.56	0.77	0.72	0.37*	0.42	2.06	1.88
MBSR modified	0.08	0.21	0.46	0.62	0.29	0.35	0.19	0.58	0.30	0.27	− 0.21	1.88
Meditation	0.06	0.29	0.72	1.15	0.91**	0.41	1.10	0.70	1.21***	0.33	− 1.21	2.043
Mindfulness	0.31	0.21	0.58	0.64	0.31	0.35	0.12	0.59	0.17	0.28	− 0.17	1.69
Movement	0.17	0.30	0.63	0.85	0.36	0.37	− 0.31	0.60	0.20	0.26	− 0.21	1.98
Multimodal	*Base*	*Base*	0.56	0.65	0.88**	0.36	0.85	0.53	0.09	0.25	− 0.25	1.75
Intervention characteristics												
Type of control group^1^												
Passive	0.07	0.36	0.39	0.93	0.12	0.26	0.85*	0.50	0.20	0.24	0.90	1.22
Wait-list	0.01	0.20	− 0.05	0.51	0.06	0.18	− 0.40	0.27	0.39**	0.18	− 0.20	1.09
Weeks	− 0.02	0.03	− 0.09	0.06	− 0.04***	0.02	− 0.02	0.04	− 0.00	0.02	− 0.12	0.09
Homework	0.24	0.17	0.23	0.43	− 0.31*	0.18	− 0.29	0.25	− 0.17	0.16	0.66	0.92
In-class	0.08	0.28	0.98	0.66	0.56**	0.27	0.15	0.46	0.08	0.24	0.18	1.15
Online	− 0.02	0.24	0.02	0.71	0.10	0.29	0.28	0.40	0.30	0.19	0.71	1.32
One-on-one	0.24	0.40	1.48*	0.79	0.43*	0.25	− 0.16	0.38	− 0.11	0.29	2.10	1.34
Additional material	− 0.10	0.15	− 0.24	0.56	0.09	0.19	− 0.08	0.29	− 0.21	0.21	− 0.56	0.93
Population characteristics												
Share female	− 0.00	0.00	− 0.00	0.01	0.01***	0.00	0.01**	0.01	− 0.00	0.00	− − 0.01	0.02
Mean age	− 0.00	0.02	− 0.11**	0.05	− 0.02	0.01	− 0.02	0.02	− 0.01	0.01	0.01	0.11
*τ*^2^	0.08		0.43		0.26		0.45		0.09		1.75	
*I*^2^	94.93		98.50		99.09		98.91		96.23		99.53	
*R*^2^	0.00		47.77		43.14		14.75		44.45		4.49	

Mixed-effects model not conducted for resilience due to few observations. *b*: estimate; *se*: standard error. ^1^Base is active control group. ***p < 0.001; **p < 0.01; *p < 0.05

Among all included intervention characteristics, (additional) one-on-one sessions seem to increase effect sizes for well-being (*b* = 1.48, *p* < 0.05) and mental health outcomes (*b* = 0.43, *p* < 0.05). For mental health outcomes, in-class interventions seem to generate larger effect sizes (*b* = 0.56, *p* < 0.01). For physical health outcomes, some degree of heterogeneity can be explained by variations in effect sizes by type of control group, suggesting that studies with wait-list control groups provide larger effect sizes than studies with active control groups (*b* = 0.39, *p* < 0.01). For the outcome stress, studies with passive control groups provide on average larger effect sizes (*b* = 0.85, *p* < 0.05) than studies with active control groups. Interventions with homework seem to generate slightly smaller effect sizes for mental health outcomes (*b* =  − 0.31, *p* < 0.05) than interventions in which there is no obligatory homework. Effect sizes of mental health outcomes decrease with increasing intervention length (*b* =  − 0.04, *p* < 0.001). Finally, effect sizes of mental health outcomes (*b* = 0.01, *p* < 0.001) and stress (*b* = 0.01, *p* < 0.01) increase with a larger share of female participants.

The results further suggest that online interventions are just as effective as analogue interventions and that additional material provided for self-practice has no impact on effect sizes. Regressions were also conducted for all outcome groups with risk of bias domains and separately with measurement instruments (e.g., for mindfulness, Five Facet Mindfulness Questionnaire, Mindful Attention and Awareness Scale). Neither differences in risk of bias assessments nor variations by measurement instrument explained heterogeneity in effect sizes.

Due to the limited number of studies performing short-term and long-term follow-ups, meta-regressions could not be conducted beyond post-intervention time points. However, a continuous time variable was included in regressions for all outcomes to detect the potential rate of depreciation of effect sizes over time. The continuous time variable provided no significant results for any of these outcomes.

## Discussion

Based on 91 eligible studies (from 92 publications), including 4927 participants and 4448 controls, the present synthesis shows that MBIs and MIIs significantly improve all seven overarching outcome categories. For mindfulness, stress, well-being outcomes, mental and physical health, and work-related outcomes, average effect sizes were small to medium, and large for resilience. Analyses of sub-categories revealed that MBIs and MIIs positively influence life satisfaction, self-compassion, subjective well-being, affect, anxiety, burnout, depression, sleep, subjective mental health, heart rate variability, pain, subjective physical health, job satisfaction, and productivity on average to a small to medium extent. Smaller or nonsignificant average effect sizes were found for relaxation, self-efficacy, psychological inflexibility, blood pressure, heart rate, absenteeism, and work engagement. Average *SMDs* at short-term follow-ups for the broad categories mindfulness, well-being, mental health, stress, and physical health remain statistically significant and of small to medium size. Small to medium average *SMDs* for the subcategories with at least four studies were found for burnout, subjective mental health, and subjective physical health. Nonsignificant effect sizes were obtained for depression, job satisfaction, and the overarching category work-related factors. Average *SMDs* in long-term follow-ups turned nonsignificant for well-being, subjective mental health, and work-related factors, while for mental health, stress and physical health, average effect sizes are small and marginally significant. Sensitivity analyses point mainly towards smaller effect sizes due to extremely high outliers. For the pooled categories mindfulness, well-being, mental health, stress and physical health, average *SMDs* dropped by 10-20% in size and remained significant. Dropping outliers from the model turned average effect sizes of self-efficacy and resilience to medium size and significant. The average *SMD* of work-related factors dropped by 50%, yet remained small and significant. The average effect sizes of almost all subcategories remained similar in magnitude after removing outliers, and increased to a large effect for self-compassion. The results of the original analyses indicate substantial heterogeneity, which is somewhat mitigated after the removal of outliers.

Several aspects of the studies may have contributed to heterogeneity. Firstly, variations in study design, including the type of intervention (e.g., MBSR vs. breathing intervention), intervention setting (e.g., group vs. individual), the duration of the intervention, and the type of control group (e.g., active control group vs. wait-list control group), may impact the results. Secondly, demographic differences in the populations studied, including factors such as age, gender, and health status, may also impact the results and increase heterogeneity. Thirdly, differences in the outcome measures (e.g., scores containing five questions vs. scores containing 20 questions) utilized may influence effect sizes in mindfulness studies. Finally, variations in the mindfulness intervention itself, such as the frequency and duration of practice and the level of practitioner experience, may also affect the results and contribute to heterogeneity. Many of these aspects have been extracted from the studies, but data are not available for all of these aspects. For example, only 71% of the included studies provided information on trainer qualifications, and this information is difficult to operationalize. Meta-regressions were run including intervention characteristics that were available for at least 90% of studies. Unfortunately, analyses of the available data provide little insight into the sources of heterogeneity. Some differences can be found by intervention type and type of control group. Here, MBSR and meditation courses tend to be more effective on average than most other formats. Studies with passive or wait-list control groups tend to show slightly larger impacts on all outcomes, though only significantly for pooled mental health and stress. For these two outcomes, a higher share of female participants is associated with larger effect sizes. In general, studies with in-class interventions and one-on-one sessions seem more effective for most outcomes, although only significantly for mental health and well-being.

In recent years, a few meta-analyses of RCTs investigating mindfulness interventions at the workplace have been published. This meta-analysis has been informed by Bartlett et al. (2019), Lomas et al. (2019), and Vonderlin et al. (2020), and adds three important features. See Table 6 for an overview of the present review characteristics. Firstly, the present meta-analysis extends the publication period of RCTs to November 2019, and therefore adds one more year to the period investigated by Vonderlin et al. (2020), and three to four years to Bartlett et al. (2019) and Lomas et al. (2019). Only Vonderlin et al. (2020) included two studies published before 2005 (our start date), which analyzed meditation interventions. As our results are generally in-line with Vonderlin et al. (2020), we believe that omitting these two studies in our meta-analysis does not generate a problem in the validation of our findings. Secondly, while target population and setting share similar features (working adults, workplace setting), the definition of interventions with respect to their mindfulness content varies. Lomas et al. (2019, p. 2) include “all forms of MBIs”, which is not further explained in the review. Bartlett et al. (2019) use a less vague inclusion restriction, namely interventions “explicitly described as mindfulness programs”, and Vonderlin et al. (2020, p. 3) include “any type of mindfulness/meditation-based intervention with at least 2 h of training and with mindfulness elements constituting at least 50% of the program”, which is a more precise description of studies. However, despite precise and easily comprehensible, only few studies provide such detailed information on the number of hours dedicated to mindfulness practice or the amount of mindfulness in relation to other topics involved in the training. In fact, we could not find this information in any of the studies selected. Therefore, we elaborated on an inclusion restriction that provided clarity and at the same time feasibility. It allowed to include interventions described as promoting mindfulness and/or self-awareness (bodily sensations, affect, or thoughts). This description considers the definition of mindfulness by Grossman (2015), as it also includes interventions that aim at promoting one of the principles of mindfulness, i.e., increased awareness about bodily sensations, affect or thoughts. The studies included in this review did not necessarily have to teach mindfulness as the core technique but use other forms that promote mindfulness indirectly. We thereby followed the description of the authors of the original studies, rather than following the literature on, for example, the mechanisms of yoga or other interventions (such as Riley & Park, 2015). For example, the yoga-based study by Alexander et al. (2015) is included, as their description contains the goal of enhancing self-awareness and one of their primary outcomes is mindfulness. Our definition allows for a wider interpretation than the one by Vonderlin et al. (2020), yet greater feasibility, and therefore allows to include mindfulness-informed interventions in addition to mindfulness-based interventions. All of the resulting interventions aim to promote mindfulness, while not necessarily being the main technique or mechanism. This inclusion restriction delivered 91 studies, which is more than all other meta-analyses in this area of research and allows for more detailed analyses on both outcome categories and moderators, as well as follow-up data. In general, results are in-line with the results of the aforementioned three meta-analyses. In comparison to Vonderlin et al. (2020), which employed a comprehensive analysis that is most similar to our research, we add novel elements to the analysis in relation to long-term assessments, and differences in moderator analyses, especially by type of intervention. Furthermore, our meta-analysis on workplace mindfulness is the first to include physiological outcomes. Despite the greater number of studies included in our analysis, we have observed similar evidence of heterogeneity and have yet to establish a consistent explanation for the variability among effect sizes. Specifically, our measures of heterogeneity are almost identical to those reported by Vonderlin et al. (2020), whom we used as a reference for our outcome clusters. Bartlett et al. (2019), who assessed very disaggregated *SMDs* based on the applied mindfulness questionnaires, reported somewhat lower indicators of heterogeneity. Similarly, Lomas et al. (2019) reported slightly lower heterogeneity indicators, albeit still within the medium to high range, as their study employed a more focused aggregation of outcome categories, but analyzed a smaller number of outcomes overall. Hence, the amount of heterogeneity does not appear to depend on the definition of interventions (mindfulness-based only, or also mindfulness-informed), but rather on the level of disaggregation of average *SMDs*.

**Table 6:** Comparison of meta-analyses

Meta-analysis/characteristics	Lomas et al. (2019)	Bartlett et al. (2019)	Vonderlin et al. (2020)	Present meta-analysis
Inclusion restrictions	• RCTs • “all forms of MBI” • professional populations • published (or in press) in English in a peer-reviewed academic journal • before January 2016	• RCTs • interventions “explicitly described as mindfulness programs” • organized by employers and delivered for staff within the work context • published in English before May 2016	• RCTs • “any type of mindfulness/meditation-based intervention with at least 2 h of training and with mindfulness elements constituting at least 50% of the program” • sample of healthy adults (age 18-65 years) with close to full-time employment (> 30 h/week) • programs offered at the workplace or initiated by the employer • published in English before Nov 2018	• RCTs • Interventions described as promoting mindfulness and/or awareness of bodily sensations, affect, or thoughts • offered in the workplace or initiated by the employing organization • working adults are the target population • published in peer-reviewed journals in English or German between 2005 and Nov 2019
Number of studies included in meta-analysis	35	23	53	91
Outcomes analyzed	Anxiety, Burnout, Depression, Distress and anger, Stress and strain, Compassion and empathy, Emotion regulation, Job performance (Efficacy), Mindfulness and awareness, Physical health (physical activity and sleep quality), positive well-being (life satisfaction, positive affect and resilience)	Mindfulness, Stress, Mental health (Psychological Distress, Depression, Anxiety, Burnout), Well-being (general Well-being, Sleep problems, and Work performance	Mindfulness, Mental well-being (Well-being and life Satisfaction, Compassion), Stress and Health Impairment (Stress, Subsyndromal Symptoms, Burnout, Somatization and physical illness), Work-related outcomes (Work Engagement, Productivity, Job Satisfaction), Resilience	Mindfulness, Well-being (Life satisfaction, Relaxation, Self-compassion, Subjective well-being, Self-efficacy), Mental health (Affect, Anxiety, Burnout, Depression, Psychological Inflexibility, Sleep, Subj. psychological health), Stress, Resilience, Physical health (Blood pressure, Heart rate, Heart rate variability, Pain, Subj. physical health), Work-related factors (Absenteeism, Job satisfaction, Productivity, Work engagement)
Moderators analyzed	MBSR (versus other interventions), the use of retreatments, region (North America versus the rest of the world), type of sample (students versus professionals), year of study, length of intervention, mean age of participants, percentage of women, QATQS score	Delivery mode (weekly f2f vs other format), Does contact time (class hours up to 7 vs. 8 or more; weeks up to 7 vs. 8 or more), Does homework (up to 10 min vs. 10 or more minutes), Content (stress physiology included vs. not included, mindfulness theory included vs. not included, movement/yoga included vs. not included, Micropractice included vs. not included), Industry (human services vs. other)	Hours of attendance, Gender, Occupational profession, Participants’ level of education, work experience, age of participants, type of program, time span in weeks, method and location of delivery, recommended hours of practice at home, type of control group, ITT samples, or publication date	Intervention type (ACT modified, Breathing, MBSR, MBSR modified, Meditation, Mindfulness, Movement, Multimodal), Intervention characteristics (Type of control group, Weeks of practice, Homework, In-class, Online, One-on-one, Additional material), Population characteristics (Share female, Mean age), Study quality, measurement instrument

### Limitations and Future Directions

One of the main limitations of this review is the large heterogeneity in the results for almost all outcomes. With this heterogeneity at hand, the validity of the overall results is limited, as the calculated *SMDs* do not accurately reflect the combined results of the studies. With substantial heterogeneity, it is challenging to draw reliable conclusions about the overall effectiveness of mindfulness-based and mindfulness-informed interventions. By applying a random effects model, we aimed to mitigate the effect of heterogeneity on *SMDs*. Nonetheless, the identification of heterogeneity in the included studies suggests that comparability might not be attainable. Our approach to the amalgamation of mindfulness-based and mindfulness-informed interventions is based on the premise that both formal and informal mindfulness practices have the capacity to modify brain function, including amygdala function, which can have a consequential impact on individuals’ daily life beyond the practice of mindfulness per se, as demonstrated by previous research (Gotink et al., 2016; Hölzel et al., 2010; Kral et al., 2018). Hence, it appears reasonable to differentiate between mindfulness-based and mindfulness-informed interventions when the aim is to define their techniques and interventions. However, during the evaluation of the effectiveness of such interventions, this distinction might be extraneous, given that the lived experience of practitioners is not contingent on the type of intervention, but rather on the extent of changes in the brain that arise as a consequence of the practice and are perceived in everyday life (Hölzel & Ott, 2006). Notably, we identified that interventions within the groups of mindfulness-based and mindfulness-informed interventions exhibit heterogeneity, as well as within our eight subgroups. Thus, forthcoming systematic reviews ought to place greater emphasis on the frequency and nature of formal and informal mindfulness practices, and primarily analyze whether mindfulness has been improved as a result of the intervention. Additionally, future reviews should focus on specific techniques (e.g., body scan, alternate nostril breathing, mindful eating, yoga asana) instead of multi-component interventions. A cut-off point could be established to delineate which interventions effectively enhance mindfulness, and only these could be deemed as mindfulness interventions. The secondary outcomes of these more narrowly defined mindfulness interventions, such as reduced burnout risk and improved physiological parameters, could then be evaluated. It is also critical to endeavor to obtain further characteristics of interventions, for instance, by conducting interviews with the corresponding authors of published studies of mindfulness RCTs. These intervention characteristics can then be used to conduct sub- and meta-analyses. In future RCTs on the topic, researchers should use clear and consistent definitions and report as much information as possible when publishing their studies. It would be helpful if a group of researchers developed a comprehensive reporting list for social-psychological RCTs, comparable to the PRISMA checklist for systematic reviews. This checklist should then be obligatory to apply in future RCTs. Furthermore, future research employing RCTs benefits from including a homogeneous population in their study to reduce heterogeneity in meta-analyses. For example, they can limit their study to a specific age group, gender, or health condition (with or without psychological symptoms). This can help ensure that the studies are comparable. Standardized protocols may be one way to achieve more homogenous mindfulness interventions, or single techniques, and therefore more homogenous results across studies. Standardized protocols might have an impact upon effectiveness, as we have found that standardized MBSR interventions were more effective for well-being, mental and physical health outcomes. However, mindfulness is a multifaceted phenomenon that can be trained by various techniques. Also, trainers of mindfulness are diverse in their beliefs about what mindfulness is and how it can be taught.

Another main limitation of the data underlying this systematic review and meta-analysis is the presence of large outliers. Sensitivity analyses show that for some outcomes, results differ considerably when accounting for extreme outliers. These extreme outliers can be traced back to differences between outcome measures before intervention even in large group sizes, which hints at a poor randomization process.

For almost all studies, we detected a high risk of bias in all domains. In other reviews with psychosocial interventions, the risk of bias is similarly high (Bruin, 2015). The risk assessment here may be considered more conservative as in Vonderlin et al. (2020), as they did not judge the risk for two domains that usually result in high risks: performance bias and detection bias. These were included here, however, because some of the analyzed studies (e.g., Cheema et al., 2013) aimed at solving the problem of blinding staff and participants. In fact, some studies consistently show low to moderate risks of bias for all five domains (e.g., Baby et al., 2019; Dwivedi et al., 2015, 2016). This suggests that it is also possible to obtain robust results in the field of mindfulness intervention research. Especially, we believe that better RoB results can be achieved when study conductors take into account RoB-domains when planning interventions and summarizing findings. Taking into account the RoB assessment in this review, a solid statement on the evidence of the effectiveness of the presented mindfulness interventions on the target parameters is only possible to a limited extent and future research is necessary. As we did not detect any reporting bias, overall results are credible with respect to missing information.

The literature search was limited to the year 2019, which was shortly after the review protocol was published. The time lag between search and publication of results does not seem to influence up-to-datedness; however, as in following mindfulness research, we did not observe any large innovations or outstanding publications that would significantly change overall results.

The amount of publications detected has led to a number of changes compared to the original protocol. Most of them, however, were minor. Two major differences exist: Firstly, we categorized outcomes instead of analyzing each outcome individually. As explained, similarly to previous meta-analyses on the topic (Bartlett et al., 2019; Lomas et al., 2019; Vonderlin et al., 2020), categorizations were made to guarantee comparability of results. Secondly, the large amount of studies included permitted estimation of meta-regressions with the aim of identifying influences on average *SMDs* by study characteristics or mindfulness intervention types.

As outlined above, risk of bias was generally high. On the other hand, we detected no publication bias, suggesting no considerable lack in diversity of results.

Similarly to previous studies on this topic, the present review is subject to methodological limitations of the investigated RCTs. Generally, we suggest that in future RCTs on the topic, attention should be paid to the following aspects that improve methodological quality and reduce the risks of bias in the results obtained (Michaelsen et al., 2021). Firstly, a study protocol should be published before the start of the study. Secondly, a random number generation algorithm should be applied and the name of the tool should be reported. Thirdly, future RCTs should include sufficiently large study populations. Fourthly, different persons should be appointed for contact with participants and for the review of results. Fifthly, blinding of participants into intervention and control groups to outcome assessors should be guaranteed. Sixthly, it should be aimed for a reduction of drop-out rates and statistical methods to compensate for attrition should be applied. Lastly, study teams should consider implementing possibilities to verify actual exercise time.

Due to a limited number of studies, we are currently unable to draw firm conclusions about the long-term effectiveness. Therefore, we also suggest that future research aims to examine outcomes of occupational MBIs up until twelve months after the end of the intervention as well as include so far rarely investigated aspects, such as resource use and work ability, that also allow conclusions about productivity and efficiency. The latter might be especially relevant for decision making in the business context. Ideally, future publications of RCTs would also report on intervention characteristics, such as dosage and location, as well as information on the organizational environment, e.g., company size and work cultural aspects. Finally, despite no hints of publication bias in this review (in comparison to Bartlett et al., 2019, and Vonderlin et al., 2020), the research community should aim at publishing all results despite negative or null effects.

The findings suggest that policy makers should exercise caution when using these results to inform decision-making, as the variability in outcomes makes it challenging to identify the optimal course of action and allocate resources effectively. It is particularly noteworthy that the present review did not establish the cost-effectiveness of workplace mindfulness interventions, which should be taken into account by policy makers. In the evaluation of cost-effectiveness, various factors must be considered, including absenteeism, incapacity to work, productivity loss, employee turnover, and staff ratios. From the perspective of the healthcare system, the utilization of health care services, such as medical consultations for work-related accidents, is also a crucial indicator. The financial valuation of these factors is subject to variation across the existing literature. In this review, four different research articles (Hartfiel et al., 2017; Singh et al., 2016, 2020; van Dongen et al., 2016) have been included that analyzed distinct cost-effectiveness parameters. However, these studies included different parameters, so that no analysis of these factors was performed.

For business leaders, the results suggest that it is feasible and rewarding to integrate MBIs and MIIs in the workplace because of potentially visible effects on various domains related to mindfulness, physical and mental health parameters, well-being, stress, and work-related aspects. The effects are also sustainable as shown in the short-term and long-term follow-ups. As the populations of employees within the included studies were relatively diverse in terms of professions and sectors, these generally positive results seem to be applicable across various settings. Especially interesting is the finding that digital MBIs and MIIs appear to be as effective as analogue interventions, highlighting its suitability also in home-office contexts, for teams who work in different locations or other complex work environments, or as a cost-saving measure. Also remarkable is the result that post-intervention effectiveness does not seem to depend on the duration of an intervention, except for mental health outcomes.

Despite a general recommendation to implement mindfulness interventions in work settings, the review does not allow to provide specific advices on which specific mindfulness intervention to implement under which circumstances. Especially, certain structural and cultural aspects of an organization, and specific aspects of interventions that could not be captured in our analysis (e.g., size of the company, values of the company, spiritual content of the intervention) could potentially influence the effectiveness. In addition, we were not able to analyze more specific outcomes, such as aggressiveness, creativity or empathy, as well as the cost-effectiveness of MBIs and MIIs, as only few RCTs included relevant measurements. Because of large heterogeneity in the results, practitioners should consider individual factors, such as the general stress level of employees, their health history, and preferences, when making decisions about an intervention.

In conclusion, the present study employed a comprehensive meta-analytical approach to consolidate and synthesize the findings of a considerable number (*k* = 91) of RCTs that examined the efficacy of mindfulness-based interventions (MBIs) and mindfulness-informed interventions (MIIs) in various workplace settings. While the current review substantiates the favorable effects of MBIs and MIIs across all outcomes, it is worth noting that some of the results should be approached with a degree of caution, as certain outcomes may have an overestimated average *SMD*, as indicated by sensitivity analyses. Nevertheless, despite the stringent exclusion criteria for positive outliers, all the effects remain statistically significant. The decision-makers of organizations must examine which outcomes are relevant to them when determining whether to adopt mindfulness interventions and which intervention to implement, while also considering the results of long-term follow-up studies conducted according to rigorous quality of reporting guidelines. Future systematic reviews could focus on particular mindfulness enhancing techniques and present more in-depth outcomes.

## Supplementary Information

Below is the link to the electronic supplementary material.

Supplementary file1 (DOCX 19 KB)

Supplementary file2 (DOCX 21 KB)

Supplementary file3 (DOCX 105 KB)

Supplementary file4 (DOCX 45 KB)

Supplementary file5 (PDF 286 KB)

Supplementary file6 (DOCX 6096 KB)

## Data Availability

Template of data collection forms, data used for all analyses, and analytic code can be obtained from the corresponding author upon request.
