# Supplementary material for: Mindfulness-Based and Mindfulness-Informed Interventions at the Workplace: A Systematic Review and Meta-Regression Analysis of RCTs
Source: Mindfulness (N Y). 2023 May 11:1–34. Online ahead of print. doi: 10.1007/s12671-023-02130-7 (PMC10172073; doi:10.1007/s12671-023-02130-7)
Supplement: Supplementary file 2 — Supplementary file2 (DOCX 21 KB) [file 12671_2023_2130_MOESM2_ESM.docx]

**Supplementary Online Material 2 – Search codes**

The search was based on the following search terms:

- Intervention: mindfulness, meditation, breathing exercise, yoga, tai chi, qi gong / qigong, body-scan, mantra, mind-body therapy.
- Population and setting: work, workplace, job, occupation, employment, employee*, manager
- Control group or study design: controlled-randomized, randomized, RCT, random, controlled study, intervention, control group

These terms were also translated into German and the same search strategy was used. Manual searches in reference lists of retrieved publications and other systematic reviews as well as hand search in Google Scholar ([scholar.google.de](https://scholar.google.de)) were pursued.

Table S2.1 Search Codes

| Database | search code | Date search was pursued |
| --- | --- | --- |
| PubMed | (mindfulness[Title/Abstract] OR mindful[Title/Abstract] OR meditation[Title/Abstract] OR breathing[Title/Abstract] OR Yoga[Title/Abstract] OR "tai chi"[Title/Abstract] OR "qi gong"[Title/Abstract] OR qigong[Title/Abstract] OR "body scan"[Title/Abstract] OR chanting[Title/Abstract] OR mind-body[Title/Abstract]) AND (work[Title/Abstract] OR workplace[Title/Abstract] OR working[Title/Abstract] OR occupation[Title/Abstract] OR occupational[Title/Abstract] OR job[Title/Abstract] OR employed[Title/Abstract] OR employment[Title/Abstract] OR employee[Title/Abstract] OR labo?r[Title/Abstract] OR staff[Title/Abstract]) AND (RCT[Title/Abstract] OR "randomi?ed-controlled"[Title/Abstract] OR "randomi?ed controlled"[Title/Abstract] OR "controlled trial"[Title/Abstract] OR "random allocation"[Title/Abstract] OR "control group"[Title/Abstract] OR intervention[Title/Abstract]) AND ("2005/01/01"[PDAT] : "2019/11/28"[PDAT]) AND ((English[lang] OR German[lang])) | 28.11.2019 |
| PsycINFO | Abstract:(mindfulness OR mindful OR meditation OR breathing OR Yoga OR "tai chi" OR "qi gong" OR qigong OR "body scan" OR chanting OR mind-body) AND (work OR workplace OR working OR occupation OR occupational OR job OR employed OR employment OR employee OR labo?r OR staff) AND (RCT OR “randomi?ed-controlled” OR “randomi?ed controlled” OR “controlled trial” OR “random allocation” OR “control group” OR intervention) | 27.11.2019 |
| PubPsych | ((mindfulness OR Achtsam* OR Meditation OR Atem* OR Yoga OR "tai chi" OR "qi gong" OR qigong OR "body scan" OR chanting OR "mind-body") AND (Arbeit* OR Beschäftig* OR job OR Beruf OR Mitarbeiter OR Gesundheitsmanagement OR Betrieb* OR job) AND (RCT OR randomisiert-kontrolliert OR randomized-controlled OR “randomized controlled” OR Kontrollgruppe OR Intervention OR zufällig)) PY>=2005 PY<=2019 | 28.11.2019 |
| Cochrane Central Register of Controlled Trials | (mindfulness OR mindful OR meditation OR breathing OR Yoga OR "tai chi" OR "qi gong" OR qigong OR "body scan" OR chanting OR mind-body) AND (work OR workplace OR working OR occupation OR occupational OR job OR employed OR employment OR employee OR labo*r OR staff) AND (RCT OR “randomi*ed-controlled” OR “randomi*ed controlled” OR “controlled trial” OR “random allocation” OR “control group” OR intervention) in Title Abstract Keyword | 28.11.2019 |
| Scopus | TITLE-ABS-KEY ( ( mindfulness  OR  mindful  OR  meditation  OR  breathing  OR  yoga  OR  {tai chi}  OR  {qi gong}  OR  qigong  OR  {body scan}  OR  chanting  OR  {mind-body} )  AND  ( work  OR  workplace  OR  occupation  OR  occupational  OR  job  OR  employed  OR  employment  OR  employee  OR  labor  OR  staff )  AND  ( rct  OR  {randomized-controlled}  OR  {randomized controlled}  OR  {controlled trial}  OR  {random allocation}  OR  {control group}  OR  intervention ) )  AND  PUBYEAR  >  2004  AND NOT  INDEX ( medline )  AND  ( LIMIT-TO ( LANGUAGE ,  "English" )  OR  LIMIT-TO ( LANGUAGE ,  "German" ) ) | 28.11.2019 |
