# Supplementary material for: Mindfulness-Based and Mindfulness-Informed Interventions at the Workplace: A Systematic Review and Meta-Regression Analysis of RCTs
Source: Mindfulness (N Y). 2023 May 11:1–34. Online ahead of print. doi: 10.1007/s12671-023-02130-7 (PMC10172073; doi:10.1007/s12671-023-02130-7)
Supplement: Supplementary file 3 — Supplementary file3 (DOCX 105 KB) [file 12671_2023_2130_MOESM3_ESM.docx]

**Supplementary Online Material 3: Description of outcome categories and instruments assigned to outcome categories**

Table S3.1 Description of outcome categories

| **Outcome subcategories** |
| --- |
| *Blood pressure* is usually measured non-invasively in the form of systolic and diastolic pressure using a blood pressure monitor. Elevated blood pressure is associated with stressful experiences, and it has been scientifically proven that blood pressure can be lowered through meditation exercises (Goldstein et al. 2012). |
| *Heart rate* is an autonomic function of the body and is defined as the number of heartbeats per unit of time. Mindfulness and meditation exercises can lower the heart rate (Khalsa et al. 2015). |
| *Heart rate variability (HRV)* describes the changes in heart rhythm. This is influenced by the activities of the autonomic nervous system, which regulates tension and relaxation. Under stress, the heart rhythm tends to be high, while at rest it tends to be low. A low HRV indicates a weak adaptability of the body to the affective, cognitive and psychological aspects of stress. In contrast, high HRV is associated with a better ability to regulate stress (Hansen et al. 2004; Lehrer 2009). |
| *Pain*: In contrast to the physiological parameters, pain is a self-assessed parameter. Pain is usually assessed by means of visual analogue scales. |
| *Subjective physical health* includes instruments that capture participants' subjective assessment of their physical health status. These include, for example, various subscales of the Medical Outcomes Study, such as the Short Form 36 (SF-36) and the Patient Health Questionnaire with nine questions. |
| *Depression*: According to the World Health Organization (WHO 2020), depression is “a common mental disorder that may be characterized by sadness, disinterest and loss of enjoyment, feelings of guilt and low self-esteem, sleep disturbances, loss of appetite, fatigue and poor concentration. It can occur over a long period of time or recurrently and can affect a person’s ability to work, learn or simply live. In the worst cases, depression can lead to suicide.” Further information on symptomatology and diagnostics has been prepared, for example, by the Robert Koch Institute (Bretschneider et al. 2017). A number of studies have shown the reduction capacity of mindfulness methods on depression-associated symptoms (Cramer et al. 2013). |
| *Stress* is usually a sudden, threatening or simply challenging situation that forces a reaction. It triggers physiological adaptation mechanisms, the so-called stress response (Esch et al. 2005). Such endogenous or auto-regulatory reactions aim to enable people to react adequately, if at all possible, to extraordinary situations (Esch 2002). It is important to emphasize that this reaction does not necessarily have to be experienced as such. It is therefore possible to be stressed without feeling or knowing it or evaluating it negatively (Matthews 2016). Unlike identified disorders, stress is more general in its definition and measurement, encompassing psychophysiological and behavioral symptoms that are not specific to a particular disorder, including anxious and depressive reactions (Lomas et al. 2019). Stress experience is associated with various disorders (Esch et al. 2002). |
| *Anxiety*: According to the State-Trait Anxiety Inventory (Spielberger et al. 1970), state anxiety is defined as a “transient momentary emotional state resulting from situational stress”. Dispositional anxiety is the “predisposition to react with anxiety in stressful situations”. Characteristic of generalized anxiety disorder are worries that relate to real dangers, whereby their risk of occurrence is greatly overestimated. These worries can be transferred to numerous areas and result in protection and avoidance behavior (Bandelow et al. 2013). |
| *Subjective mental health* includes various instruments that allow participants to self-assess their mental health, for example using the Psychological General Well-Being Index (Grossi und Compare 2014). Intrapersonal affective or emotional states that reflect a sense of subjective well-being or distress are recorded here. |
| *Affect* is usually understood as an intense, relatively short-lasting feeling. Basically, the current affective state must be distinguished from the comparatively stable habitual tendency to experience affect (trait) (Eschenbeck 2009). In the studies presented here, the contemporary experience of both negative and positive affects was evaluated. The Positive and Negative Affect Scale (PANAS) (e.g., Watson et al. 1988) was used in most of the studies examined here. |
| *Psychological inflexibility* refers to the inability to maintain or change behavior in terms of long-term value-compliant goals with the intention of avoiding unpleasant emotions (Bond et al. 2011). Psychological inflexibility results in the inability to engage with new situations. A reduction through mindfulness-informed practices has been demonstrated in some studies (Lloyd et al. 2013). |
| *Life satisfaction* is defined in the context of the *Satisfaction with Life Scale* as “a global assessment of a person’s quality of life according to his or her chosen criteria” (Shin und Johnson 1978). |
| *Job satisfaction* is the evaluative assessment of a person’s work situation (Weiss 2002). Job satisfaction refers to important organizational outcomes such as tasks and contextual performance. Because of its similarity to life satisfaction, it is subsumed in the well-being category. |
| *Subjective well-being* is the assessment of one’s own level of happiness and life satisfaction (Myers et al. 2008). The most commonly used instrument is the *WHO Five Well-being Index*, which consists of five questions. As with life and job satisfaction, there is an expected positive correlation between subjective well-being and mindfulness (Keng et al. 2011). |
| *Life satisfaction* is defined in the context of the *Satisfaction with Life Scale* as “a global assessment of a person’s quality of life according to his or her chosen criteria” (Shin und Johnson 1978). |
| *Relaxation*: This category measures how well a person can relax during free time and recover from work (Sonnentag und Fritz 2007). A positive correlation between practicing mindfulness techniques and the ability to relax has already been shown (Baer 2003). |
| *Sleep*: This category includes sleep quality and quantity as well as various sleep disorders (e.g., insomnia). Neurobiological studies have shown that meditation leads to a change in the melatonin balance and can thus positively influence the sleep-wake rhythm (Esch 2014; Michaelsen und Esch 2021). Several studies have already shown that sufficient and good sleep is beneficial for health, well-being and performance (Magnavita und Garbarino 2017) and is therefore widely investigated in the mindfulness studies conducted in the work context. |
| *Resilience* describes the process of coping with adversity, trauma, threats or significant stressful experiences and recovering (“bouncing back”) one’s initial level of strength, presence and satisfaction in a timely manner (Joyce et al. 2018). Resilience is assessed through self-assessment, for example on the basis of the *Connor-Davidson Resilience Scale* (Connor und Davidson 2003). |
| *Mindfulness*: In the present studies, mindfulness is examined as a target parameter. Mindfulness is thus seen as a trait or learnable skill and operationalized through different questionnaires (Davidson 2010). |
| *Self-compassion* is the friendly, understanding and respectful attitude towards oneself in situations of suffering (Neff und Germer 2013). Because of the attention to oneself, mindfulness is a central component of self-compassion (Neff und Dahm 2015). A review by Wilson et al. (2019) demonstrated a positive effect of mindfulness-based practices on self-compassion. |
| S*elf-efficacy* deals with a person’s belief in their ability to influence events that affect their life (Bandura 2010). In the present studies, various work-specific aspects of self-efficacy were measured through subjective assessments, for example, the assessment of caring ability among healthcare workers. The category of self-efficacy here also includes the related self-achievement, which can also serve as a resource in dealing with stress (Hallsten et al. 2005). |
| *Burnout* is a state of exhaustion that can result from chronic overwork. Burnout is characterized by emotional exhaustion, cynicism and reduced performance (Leiter et al. 2015). In mindfulness studies conducted in the setting of work, burnout is one of the most studied parameters. |
| *Work engagement* is characterized by a positive work-related state of fulfilment (Schaufeli et al. 2006). The most frequently used instrument in the present studies is the *Utrecht Work Engagement Scale* as well as questions developed by the authors themselves for participants on their work engagement. |
| *Absenteeism* describes absence from work due to various causes such as illness or lack of motivation (Jahn 2014). It is calculated as the average number of days absent per person employed in a company within a certain period of time. In the context of the present studies, absenteeism is self-reported. |
| *Productivity*: In this study, productivity describes the work performance of an employee. This is partly self-assessed or collected at the company level. Productivity indicators here include absolute and relative presentism, i.e. showing up for work despite illness. These two aspects were measured using the *World Health Organization Health and Work Performance Questionnaire*, which calculates the difference between self-assessed performance and the average performance level of the reference group (here the other participants in the intervention) (absolute presentism) or is defined as the ratio of one’s own performance compared to the possible performance measured by the average performance of the other colleagues/participants, i.e. relative presentism (Kessler et al. 2004; Kessler und Ustün 2004). |

Table S3.2 Instruments assigned to outcome categories

| **Fine outcomes (25)** | **Name of instrument: sub-scale of instrument (number of items)** |
| --- | --- |
|  |  |
| Blood Pressure (BP) | Blood pressure |
|  | Blood pressure: Diastolic |
|  | Blood pressure: Systolic |
| Heart rate (HR) | Heart rate |
| Heart rate variability (HRV) | HRV |
|  | HRV: HF |
|  | HRV: LF |
|  | HRV: LF neutral |
|  | HRV: LF recovery |
|  | HRV: LF stressor |
|  | HRV: LF/HF |
|  | HRV: pNN50 |
|  | HRV: RMSSD |
|  | HRV: RR interval |
|  | HRV: SDNN |
| Pain | Body pain (visual-analogue scale): low back |
|  | Body pain (visual-analogue scale): upper arm or neck |
|  | Center for Epidemiological Studies Depression Scale (CES-D): average pain |
|  | Center for Epidemiological Studies Depression Scale (CES-D): current pain |
|  | Center for Epidemiological Studies Depression Scale (CES-D): worst pain |
|  | Medical Outcomes Study (SF-36): bodily pain |
|  | Pain |
|  | Pain (Visual Analog Scale) |
|  | Roland Morris Disability Questionnaire (RMDQ) |
|  | Symptom Checklist 90-Revised (SCL-90-R): somatization |
| Subjective physical Health (SubjPhysHealth) | Daily Physical Symptom Checklist (DPS): Gastrointestinal subscale |
|  | Daily Physical Symptom Checklist (DPS): General Aches subscale |
|  | EuroQol (EQ-5D): utility score |
|  | EuroQol (EQ-5D-5L) |
|  | General Health Questionnaire (GHQ-12) |
|  | General Health Questionnaire (GHQ-30) |
|  | General Health Questionnaire (GHQ-28): general health |
|  | General Health Questionnaire (GHQ-28): somatic complaints |
|  | Health complaints |
|  | Health Questionnaire (HQ): Frequency of somatic complaints |
|  | Health Questionnaire (HQ): Intensity of somatic complaints |
|  | Medical Outcomes Study (SF-12): physical health |
|  | Medical Outcomes Study (SF-36): general health |
|  | Medical Outcomes Study (SF-36): physical functioning |
|  | Medical Outcomes Study (SF-36): role-physical |
|  | Medical Outcomes Study (SF-36): vitality |
|  | Medical Outcomes Study (SF-36): physical health |
|  | Patient Health Questionnaire (9) |
|  | Psychological General Well-Being Index (PGWBI): general health |
|  | Psychological General Well-Being Index (PGWBI): vitality |
|  | Shirom Vigor Scale: physical strength |
|  | Symptom Checklist 90-Revised (SCL-90-R): global severity index |
|  | Symptom Checklist-90 (SCL-90) |
| Affect | Affective Go/No-Go task: Total commissions |
|  | Bad moods at home (percent of time 1-100): self-designed questions |
|  | Bad moods at work (percent of time 1-100): self-designed questions |
|  | Bradburn’s Affect Experience Index (AEI): negative affect |
|  | Bradburn’s Affect Experience Index (AEI): positive affect |
|  | Explicit affect using ambulatory assessments: negative affect |
|  | Explicit affect using ambulatory assessments: positive affect |
|  | Implicit Positive and Negative Affect Test: implicit positive affect |
|  | Implicit Positive and Negative Affect Test: implicit negative affect |
|  | Positive affect throughout the day |
|  | Positive and Negative Affect Schedule (PANAS) (10): negative affect |
|  | Positive and Negative Affect Schedule (PANAS): negative affect |
|  | Positive and Negative Affect Schedule (PANAS): positive affect |
|  | Positive and Negative Affect Schedule (PANAS-X): Attentiveness |
|  | Positive and Negative Affect Schedule (PANAS-X): Jovial |
|  | Positive and Negative Affect Schedule (PANAS-X): negative affect |
|  | Positive and Negative Affect Schedule (PANAS-X): Reduced fatigue |
|  | Positive and Negative Affect Schedule (PANAS-X): Reduced fear |
|  | Positive and Negative Affect Schedule (PANAS-X): Reduced guilt |
|  | Positive and Negative Affect Schedule (PANAS-X): Reduced hostility |
|  | Positive and Negative Affect Schedule (PANAS-X): Reduced sadness |
|  | Positive and Negative Affect Schedule (PANAS-X): Reduced shyness |
|  | Positive and Negative Affect Schedule (PANAS-X): Self-assured |
|  | Positive and Negative Affect Schedule (PANAS-X): Serenity |
|  | Momentary negative affect: (self-designed, 2) |
|  | Momentary positive affect: (self-designed, 2) |
| Anxiety | Anxiety (visual analog scale) |
|  | Beck Anxiety Inventory |
|  | Brief Symptom Inventory: anxiety |
|  | CDC Healthy Days - Days Anxious (part of CDC HRQOL-14) |
|  | Depression, Anxiety, and Stress Scale (DASS): anxiety |
|  | General Health Questionnaire (GHQ-28): anxiety |
|  | Generalised Anxiety Disorder scale (GAD-7) |
|  | Hospital Anxiety and Depression Scale (HADS) |
|  | Hospital Anxiety and Depression Scale (HADS): anxiety |
|  | Mood & Anxiety Symptoms Questionnaire-30 (MASQ-30): anxiety |
|  | Profile of Mood States (POMS): Anxiety |
|  | PROMIS: instrument for sleep disturbance (short form): anxiety |
|  | Self-Rating Anxiety Scale |
|  | Smith Anxiety Scale (SAS) |
|  | State-Trait Anxiety Inventory (STAI) |
|  | State-Trait Anxiety Inventory (STAI): state anxiety |
|  | State-Trait Anxiety Inventory (STAI): trait anxiety |
|  | Psychological General Well-Being Index (PGWBI): anxiety |
|  | Symptom Checklist 90-Revised (SCL-90-R): anxiety |
|  | Penn State Worry Questionnaire: trait worry |
|  | State worry and stressors: state worry - duration |
|  | State worry and stressors: state worry - frequency |
|  | State worry and stressors: state worry - severity |
| Depression | Beck Depression Inventory |
|  | Brief Symptom Inventory: depression |
|  | Center for Epidemiological Studies Depression Scale (CES-D) |
|  | Depression (Visual Analogue Scale) |
|  | Depression, Anxiety, and Stress Scale (DASS) |
|  | Depression, Anxiety, and Stress Scale (DASS): depression |
|  | General Health Questionnaire (GHQ-28): depression |
|  | Hospital Anxiety and Depression Scale (HADS): depression |
|  | Medical Outcomes Study (SF-36): depression |
|  | Patient Health Questionnaire (9): depression |
|  | Profile of Mood States (POMS): Depression |
|  | PROMIS: instrument for sleep disturbance (short form): depression |
|  | Psychological Distress Manifestation Scale (PDMS): Anxiety-Depression |
|  | Psychological General Well-Being Index (PGWBI): depressed mood |
|  | Self-Rating Depression Scale (SDS) |
|  | Symptom Checklist 90-Revised (SCL-90-R): depression |
| Distress | Cornell Medical Index Health Questionnaires |
|  | Cornell Medical Index Health Questionnaires: emotional distress |
|  | Cornell Medical Index Health Questionnaires: physical distress |
|  | Cornell Medical Index Health Questionnaires: total distress |
|  | General measures of psychological distress: Global Severity Index |
|  | General measures of psychological distress: Positive Symptoms Distress Index1 |
|  | General measures of psychological distress: Total of Positive Symptoms |
|  | Interpersonal Reactivity Index (IRI): personal distress |
|  | Kessler Psychological Distress Scale (10) |
|  | Mood & Anxiety Symptoms Questionnaire-30 (MASQ-30): distress |
|  | Psychological distress (mix from surveys) |
|  | Psychological Distress Manifestations at Work Scale |
|  | Symptom Checklist 90-Revised (SCL-90-R): positive symptom distress index |
| Subjective Mental Health (SubjPsychHealth) | Brief Symptom Inventory (BSI) |
|  | Brief Symptom Inventory: global symptoms |
|  | Brief Symptom Inventory: somatization |
|  | CDC Healthy Days - Days in Poor Mental Health (part of CDC HRQOL-14) |
|  | Concise Health Risk Tracking scale (CHRT): suicidal ideation |
|  | General Health Questionnaire (GHQ-12): mental health |
|  | Medical Outcomes Study: mental health (5) |
|  | Medical Outcomes Study (SF-12): mental health |
|  | Medical Outcomes Study (SF-36): emotional role functioning |
|  | Medical Outcomes Study (SF-36): emotional well-being |
|  | Medical Outcomes Study (SF-36): mental health |
|  | Psychological General Well-Being Index (PGWBI) |
|  | Psychological Well-Being Manifestations at Work Scale |
|  | Self-Report Questionnaire-20 (SRQ-20) |
|  | Shirom Vigor Scale: emotional Energy |
|  | Shirom Vigor Scale: cognitive liveliness |
|  | Symptom Checklist 90-Revised (SCL-90-R) |
|  | Symptom Checklist 90-Revised (SCL-90-R): obsessive-compulsive |
|  | Symptom Checklist 90-Revised (SCL-90-R): positive symptom total |
|  | Symptom Checklist-90 (SCL-90): positive number |
|  | Warwick Edinburgh Mental Well-being Scale |
| Psychological Inflexibility (PsychInflexibility) | Acceptance and Action Questionnaire (short) |
|  | Acceptance and Action Questionnaire-II |
|  | Brief Experiential Avoidance Questionnaire (BEAQ) |
| Stress | Calgary Symptoms of Stress Inventory (C-SOSI) |
|  | Demand Control Support Questionnaire (DCSQ): control |
|  | Demand Control Support Questionnaire (DCSQ): demand |
|  | Depression, Anxiety, and Stress Scale (DASS): stress |
|  | Effort-Reward Imbalance questionnaire: work stress |
|  | General stress (self-designed questions) |
|  | Impact of Events Scale‐Revised (IES‐R) |
|  | Implicit Association Test modified: implicit stress |
|  | Lipp Stress Symptoms Inventory (ISSL): last month |
|  | Lipp Stress Symptoms Inventory (ISSL): last week |
|  | Lipp Stress: Last 24 hSymptoms Inventory (ISSL) |
|  | Nursing Stress Scale |
|  | Occupational Stress Indicator (OSI-2) |
|  | Perceived Stress Scale (PSS) |
|  | Perceived Stress Scale (PSS) (10) |
|  | Perceived Stress Scale (PSS) (14) |
|  | Perceived Stress Scale (PSS): modified to reflect work in schools |
|  | Police Stress Questionnaire (PSQ): operational stressors |
|  | Police Stress Questionnaire (PSQ): organizational stressors |
|  | Psychological Stress Measure (9) |
|  | Questionnaire on Medical Workers' Stress (QMWS) |
|  | Self-reported job stress |
|  | Staff Stressor Questionnaire (SSQ) |
|  | Stress (1) |
|  | Stress (self-designed) |
|  | Stress Adaption Scale (60) |
|  | Uehata Stress Questionnaire (14) |
|  | Work stress (self-designed) |
|  | Work-related stress (self-designed) |
|  | Work-related stress (Visual Analogue Scale) |
|  | Work-Related Stress Indicator Tool (WSIT) |
|  | Work-related stress scale by Lan (2004) |
| Job Satisfaction (JobSatisfaction) | Brayfield-Rothe Scale |
|  | Bell Adjustment Inventory: Job |
|  | Household Income Labour Dynamics in Australia: job control |
|  | Household Income Labour Dynamics in Australia: job demand/complexity |
|  | Household Income Labour Dynamics in Australia: job security |
|  | Job in General Scale (JIG) |
|  | Job in General Scale (JIG) (short) |
|  | Job satisfaction (1) |
|  | Job satisfaction (Judge, Locke, Durham, and Kluger 1998) (5) |
|  | Job Satisfaction Questionnaire (JSQ) |
|  | Job Satisfaction Scale (2) |
|  | Job Satisfaction Scale: instrinsic job satisfaction |
|  | McCloskey/Mueller Satisfaction Scale (MMSS) |
|  | Satisfaction with work life (self-designed, 5) |
|  | Satisfaction with Work-Family Balance Scale: satisfaction with work-life balance |
|  | Work Limitations Questionnaire (WLQ) (25) |
|  | Work-Family Conflict Scale: strain-based work-family conflict |
|  | Work-Life Conflict Scale (Waumsley, Houston, & Marks 2010) (3) |
|  | Effort-Reward Imbalance Questionnaire: Effort score |
| Life Statisfaction (LifeSatisfaction) | Assessment of Quality of Life (AQoL-4D) |
|  | Bell Adjustment Inventory: Emotional |
|  | Bell Adjustment Inventory: Health |
|  | Bell Adjustment Inventory: Home |
|  | European Social Survey: Life Satisfaction |
|  | Harmony in Life Scale (HILS) |
|  | Life satisfaction (3) |
|  | Life satisfaction: single-item scale from Lucas and Donnellan (2011) |
|  | Orientation to Life Questionnaire (13) |
|  | Overall quality of life: Linear Analog Self Assessment Scale (LASA) |
|  | Overall quality of life: Linear Analog Self-Assessment Scale (LASA-12) |
|  | Satisfaction with home life (self-designed questions) |
|  | Satisfaction with life questionnaire: health |
|  | Satisfaction with life questionnaire: quality of life |
|  | Satisfaction With Life Scale (SWLS) |
| Wellbeing | General well-being (self-designed questions) |
|  | Well-being: Visual Analogue Scale |
|  | WHO-Five Well-being Index (WHO5) |
|  | Work-related well-being (self-designed) |
|  | Psychological General Well-Being Index (PGWBI): positive well-being |
| Relaxation | Measure of Current Status: relax |
|  | Recovery Experience Questionnaire: detachment from work during off-time |
|  | Recovery Experience Questionnaire: today's psychological detachment |
|  | Smith Relaxation States Inventory (SRSI-3): basic relaxation |
|  | Smith Relaxation States Inventory (SRSI-3): core-mindfulness |
|  | Smith Relaxation States Inventory (SRSI-3): transcendence |
|  | Smith Relaxation States Inventory: disposition |
| Sleep | Insomnia Severity Index (ISI) |
|  | Jenkins Sleep Scale (JSS): Non-restorative sleep |
|  | Jenkins Sleep Scale (JSS): Total sleep score |
|  | Pittsburgh Sleep Quality Index (PSQI) |
|  | Pittsburgh Sleep Quality Index (PSQI): Sleep disturbances |
|  | Pittsburgh Sleep Quality Index (PSQI): Sleep duration |
|  | Pittsburgh Sleep Quality Index (PSQI): Sleep efficiency |
|  | Pittsburgh Sleep Quality Index (PSQI): Sleep quality |
|  | Pittsburgh Sleep Quality Index: last night's levels of sleep duration |
|  | Pittsburgh Sleep Quality Index: last night's levels of sleep quality |
|  | Pittsburgh Sleep Quality Index: sleep quality and duration |
|  | PROMIS: instrument for sleep disturbance (short form) |
|  | PROMIS: instrument for sleep disturbance (short form): sleep difficulties |
|  | Sleep quantity and quality from Kahneman et al. (2004): quality of sleep |
|  | Sleep quantity and quality from Kahneman et al. (2004): quantity of sleep |
|  | Sleep (Visual Aanalog Scale) |
|  | Sleeping time: mean daily minutes asleep (recorded by Charge2 device) |
|  | Sleep quantity and quality from Kahneman et al. (2004): insomnia (7) |
| Mindfulness | Cognitive and Affective Mindfulness Scale-Revised (CAMS-R) |
|  | Cognitive and Affective Mindfulness Scale-Revised (CAMS-R) (12) |
|  | Cognitive and Affective Mindfulness Scale-Revised (CAMS-R) (7) |
|  | Experiences Questionnaire: Decentering |
|  | Five Facet Mindfulness Questionnaire (FFMQ) (14) |
|  | Five Facet Mindfulness Questionnaire (FFMQ) (39) |
|  | Five Facet Mindfulness Questionnaire (FFMQ) + Mindfulness in Teaching Scale (MTS) (5) |
|  | Five Facet Mindfulness Questionnaire (FFMQ): acting with awareness |
|  | Five Facet Mindfulness Questionnaire (FFMQ): describing |
|  | Five Facet Mindfulness Questionnaire (FFMQ): nonjudging |
|  | Five Facet Mindfulness Questionnaire (FFMQ): nonreacting |
|  | Five Facet Mindfulness Questionnaire (FFMQ): observing |
|  | Five Facet Mindfulness Questionnaire (FFMQ): overall |
|  | Five Facet Mindfulness Questionnaire Short form (FFMQ-SF): acting with awareness |
|  | Five Facet Mindfulness Questionnaire Short form (FFMQ-SF): describing |
|  | Five Facet Mindfulness Questionnaire Short form (FFMQ-SF): non-judging |
|  | Five Facet Mindfulness Questionnaire Short form (FFMQ-SF): non-reacting |
|  | Five Factor Mindfulness Questionnaire (FFM) |
|  | Freiburg Mindfulness Inventory (FMI) |
|  | Kentucky Inventory of Mindfulness Skills-Short Version (KIMS) |
|  | Mindful Attention Awareness Scale (MAAS) |
|  | Mindful Attention Awareness Scale (MAAS): state |
|  | Toronto Mindfulness Scale (TMS) |
| Resilience | Block Ego-Resilience scale (10) |
|  | Brief Resilience Scale (BRS) |
|  | Connor-Davidson Resilience Scale (CD-RISC) |
|  | Resilience Scale (14) |
|  | Resilience short scale (8) (Soucek et al. 2015) |
| Self Compassion (SelfCompassion) | Self-Compassion Scale (SCS) |
|  | Self-Compassion Scale (SCS) modified |
|  | Self-Compassion Scale (SCS) short |
|  | Self-Compassion Scale (SCS): common humanity |
|  | Self-Compassion Scale (SCS): isolation |
|  | Self-Compassion Scale (SCS): mindfulness |
|  | Self-Compassion Scale (SCS): over-identification |
|  | Self-Compassion Scale (SCS): self-judgment |
|  | Self-Compassion Scale (SCS): self-kindness |
| Self Efficacy (SelfEfficacy) | Caring Efficacy Scale |
|  | Inventory of Positive Psychological Attitudes (IPPA): self-confidence during stress |
|  | Occupational self-efficacy scale (short) |
|  | Self-Efficacy Scale: modified |
|  | Teacher Self-Efficacy Scale |
|  | Performance-based self-esteem scale |
|  | Rosenberg Self-Esteem Scale (RSES) |
| Absenteeism | Absences from work |
|  | Absenteeism |
|  | Health-related lost productive time: Absent days |
|  | Health-related lost productive time: Lost days total |
|  | Sickness Absence |
| Burnout | Copenhagen Burnout Inventory (CBI) |
|  | Experience and Evaluation of Work: need for recovery scale (11) |
|  | Maslach Burnout Inventory - Educators Survey (MBI-ES): depersonalization |
|  | Maslach Burnout Inventory - Educators Survey (MBI-ES): emotional exhaustion |
|  | Maslach Burnout Inventory - Educators Survey (MBI-ES): personal accomplishment |
|  | Maslach Burnout Inventory (MBI) |
|  | Maslach Burnout Inventory (MBI) (shortened) |
|  | Maslach Burnout Inventory (MBI): depersonalization |
|  | Maslach Burnout Inventory (MBI): emotional exhaustion |
|  | Maslach Burnout Inventory (MBI): personal accomplishment |
|  | Maslach Burnout Inventory (MBI): professional efficacy |
|  | Maslach Burnout Inventory-Educators Survey (MBI-ES) |
|  | MBI-Human Services Survey (MBI-HSS): depersonalization |
|  | MBI-Human Services Survey (MBI-HSS): emotional exhaustion |
|  | MBI-Human Services Survey (MBI-HSS): personal accomplishment |
|  | Oldenburg Burnout Inventory (OLBI) |
|  | Oldenburg Burnout Inventory (OLBI): emotional exhaustion (4) |
|  | Professional Quality of Life (ProQOL): burnout |
|  | Shirom-Melamed Burnout Measure (SMBM) |
| Work Engagement (WorkEngagement) | Utrecht Work Engagement Scale (UWES) (17) |
|  | Utrecht Work Engagement Scale (UWES) (9) |
|  | Utrecht Work Engagement Scale (UWES) (9): dedication |
|  | Utrecht Work Engagement Scale (UWES): absorption |
|  | Work Commitment |
|  | Work Engagement |
|  | Company's own productivity measure |
| Productivity | WHO Health and Work Performance Questionnaire (HPQ): absolute presenteeism |
|  | WHO Health and Work Performance Questionnaire (HPQ): relative presenteeism |
|  | Health-related lost productive time: Inefficient days |
|  | One self-designed item on daily productivity |
|  | Productivity (1) |

References

Baer, Ruth A. (2003): Mindfulness Training as a Clinical Intervention: A Conceptual and Empirical Review. In: *Clinical Psychology: Science and Practice* 10 (2), S. 125–143. DOI: 10.1093/clipsy.bpg015.

Bandelow, Borwin; Boerner J, Reinhard; Kasper, Siegfried; Linden, Michael; Wittchen, Hans-Ulrich; Möller, Hans-Jürgen (2013): The diagnosis and treatment of generalized anxiety disorder. In: *Deutsches Ärzteblatt international* 110 (17), 300-309. DOI: 10.3238/arztebl.2013.0300.

Bandura, Albert (2010): Self-Efficacy. In: Irving B. Weiner und Raymond J. Corsini (Hg.): The Corsini encyclopedia of psychology. Hoboken, NJ: Wiley (Wiley Online library).

Bond, Frank W.; Hayes, Steven C.; Baer, Ruth A.; Carpenter, Kenneth M.; Guenole, Nigel; Orcutt, Holly K. et al. (2011): Preliminary psychometric properties of the Acceptance and Action Questionnaire-II: a revised measure of psychological inflexibility and experiential avoidance. In: *Behavior therapy* 42 (4), S. 676–688. DOI: 10.1016/j.beth.2011.03.007.

Bretschneider, Julia; Kuhnert, Ronny; Hapke, Ulfert (2017): Depressive Symptomatik bei Erwachsenen in Deutschland. In: *Journal of Health Monitoring* 2 (3), S. 81–88. DOI: 10.17886/RKI-GBE-2017-058.

Connor, Kathryn M.; Davidson, Jonathan R. T. (2003): Development of a new resilience scale: the Connor-Davidson Resilience Scale (CD-RISC). In: *Depression and anxiety* 18 (2), S. 76–82. DOI: 10.1002/da.10113.

Cramer, Holger; Lauche, Romy; Langhorst, Jost; Dobos, Gustav (2013): Yoga for depression: a systematic review and meta-analysis. In: *Depression and anxiety* 30 (11), S. 1068–1083. DOI: 10.1002/da.22166.

Davidson, Richard J. (2010): Empirical explorations of mindfulness: conceptual and methodological conundrums. In: *Emotion (Washington, D.C.)* 10 (1), S. 8–11. DOI: 10.1037/a0018480.

Esch, Tobias (2002): Gesund im Stress: Der Wandel des Stresskonzeptes und seine Bedeutung für Prävention, Gesundheit und Lebensstil. In: *Gesundheitswesen (Bundesverband der Arzte des Offentlichen Gesundheitsdienstes (Germany))* 64 (2), S. 73–81. DOI: 10.1055/s-2002-20275.

Esch, Tobias (2014): Die neuronale Basis von Meditation und Achtsamkeit. In: *SUCHT* 60 (1), S. 21–28. DOI: 10.1024/0939-5911.a000288.

Esch, Tobias; Stefano, George B.; Fricchione, Gregory L.; Benson, Herbert (2002): The role of stress in neurodegenerative diseases and mental disorders. In: *Neuro endocrinology letters* 23 (3), S. 199–208.

Eschenbeck, H. (2009): Positive und Negative Affektivität. In: Jürgen Bengel und Matthias Jerusalem (Hg.): Handbuch der Gesundheitspsychologie und medizinischen Psychologie. Göttingen: Hogrefe, S. 86–91.

Flook, Lisa; Goldberg, Simon B.; Pinger, Laura; Bonus, Katherine; Davidson, Richard J. (2013): Mindfulness for teachers: A pilot study to assess effects on stress, burnout and teaching efficacy. In: *Mind, brain and education : the official journal of the International Mind, Brain, and Education Society* 7 (3), S. 1–22. DOI: 10.1111/mbe.12026.

Goldstein, Carly M.; Josephson, Richard; Xie, Susan; Hughes, Joel W. (2012): Current perspectives on the use of meditation to reduce blood pressure. In: *International journal of hypertension* 2012, S. 578397. DOI: 10.1155/2012/578397.

Grossi, Enzo; Compare, Angelo (2014): Psychological General Well-Being Index (PGWB). In: Alex C. Michalos (Hg.): Encyclopedia of Quality of Life and Well-Being Research. Dordrecht: Springer, S. 5152–5156.

Hallsten, Lennart; Josephson, Malin; Torgén, Margareta (2005): Performance-based self-esteem. A driving force in burnout processes and its assessment. Stockholm: Arbetslivsinstitutet (Arbete och hälsa, 4).

Hansen, Anita Lill; Johnsen, Bjørn Helge; Sollers, John J.; Stenvik, Kjetil; Thayer, Julian F. (2004): Heart rate variability and its relation to prefrontal cognitive function: the effects of training and detraining. In: *European journal of applied physiology* 93 (3), S. 263–272. DOI: 10.1007/s00421-004-1208-0.

Jahn, Frauke (2014): Absentismus und Präsentismus – zwei Seiten einer Medaille. In: Dirk Windemuth, Olaf Petermann und Detlev Jung (Hg.): Praxishandbuch psychische Belastungen im Beruf. Vorbeugen - erkennen - behandeln ; [Bonus-Material im Internet. 2., erw. Aufl., Stand: November 2013. Wiesbaden: Universum-Verl.

Joyce, Sadhbh; Shand, Fiona; Tighe, Joseph; Laurent, Steven J.; Bryant, Richard A.; Harvey, Samuel B. (2018): Road to resilience: a systematic review and meta-analysis of resilience training programmes and interventions. In: *BMJ open* 8 (6), e017858. DOI: 10.1136/bmjopen-2017-017858.

Keng, Shian-Ling; Smoski, Moria J.; Robins, Clive J. (2011): Effects of mindfulness on psychological health: a review of empirical studies. In: *Clinical psychology review* 31 (6), S. 1041–1056. DOI: 10.1016/j.cpr.2011.04.006.

Kessler, Ronald C.; Ames, Minnie; Hymel, Pamela A.; Loeppke, Ronald; McKenas, David K.; Richling, Dennis E. et al. (2004): Using the World Health Organization Health and Work Performance Questionnaire (HPQ) to evaluate the indirect workplace costs of illness. In: *Journal of occupational and environmental medicine* 46 (6 Suppl), S23-S37. DOI: 10.1097/01.jom.0000126683.75201.c5.

Kessler, Ronald C.; Ustün, T. Bedirhan (2004): The World Mental Health (WMH) Survey Initiative Version of the World Health Organization (WHO) Composite International Diagnostic Interview (CIDI). In: *International journal of methods in psychiatric research* 13 (2), S. 93–121. DOI: 10.1002/mpr.168.

Khalsa, Sahib S.; Rudrauf, David; Davidson, Richard J.; Tranel, Daniel (2015): The effect of meditation on regulation of internal body states. In: *Frontiers in psychology* 6, 924. DOI: 10.3389/fpsyg.2015.00924.

Lehrer, Paul M. (2009): Biofeedback training to increase heart rate variability. In: Paul M. Lehrer, Robert L. Woolfolk und Wesley E. Sime (Hg.): Principles and practice of stress management. 3rd ed. New York, London: Guilford, S. 227–248.

Leiter, Michael P.; Maslach, Christina; Frame, Kelly (2015): Burnout. In: Robin L. Cautin und Scott O. Lilienfeld (Hg.): The encyclopedia of clinical psychology. Hoboken, NJ: John Wiley & Sons, Inc, S. 1–7.

Lloyd, Joda; Bond, Frank W.; Flaxman, Paul E. (2013): The value of psychological flexibility: Examining psychological mechanisms underpinning a cognitive behavioural therapy intervention for burnout. In: *Work & Stress* 27 (2), S. 181–199. DOI: 10.1080/02678373.2013.782157.

Lomas, Tim; Medina, Juan Carlos; Ivtzan, Itai; Rupprecht, Silke; Eiroa-Orosa, Francisco José (2019): Mindfulness-based interventions in the workplace: An inclusive systematic review and meta-analysis of their impact upon wellbeing. In: *The Journal of Positive Psychology* 14 (5), S. 625–640. DOI: 10.1080/17439760.2018.1519588.

Magnavita, Nicola; Garbarino, Sergio (2017): Sleep, Health and Wellness at Work: A Scoping Review. In: *International journal of environmental research and public health* 14, 1347. DOI: 10.3390/ijerph14111347.

Matthews, G. (2016): Distress. In: George Fink (Hg.): Stress. Concepts, cognition, emotion, and behavior. London, UK: Academic Press an imprint of Elsevier (Handbook of strees, vol. 1), S. 219–226.

Michaelsen, Maren M.; Esch, Tobias (2021): Die neuronale Basis von Meditation und Achtsamkeit im Bildungskontext. In: T. Iwers und C. Roloff (Hg.): Achtsamkeit in Bildungsprozessen - Professionalisierung und Praxis: Springer, Berlin.

Myers, David G.; Reiss, Matthias; Wahl, Svenja; Hoppe-Graff, Siegfried (2008): Psychologie. 2., erw. und aktualisierte Aufl. Heidelberg: Springer (Springer-Lehrbuch).

Neff, Kristin D.; Dahm, Katie A. (2015): Self-Compassion: What It Is, What It Does, and How It Relates to Mindfulness. In: Brian D. Ostafin, Michael D. Robinson und Brian P. Meier (Hg.): Handbook of Mindfulness and Self-Regulation. 1st ed. 2015. New York: Springer, S. 121–137.

Neff, Kristin D.; Germer, Christopher K. (2013): A pilot study and randomized controlled trial of the mindful self-compassion program. In: *Journal of clinical psychology* 69 (1), S. 28–44. DOI: 10.1002/jclp.21923.

Schaufeli, Wilmar B.; Bakker, Arnold B.; Salanova, Marisa (2006): The Measurement of Work Engagement With a Short Questionnaire. In: *Educational and Psychological Measurement* 66 (4), S. 701–716. DOI: 10.1177/0013164405282471.

Shin, D. C.; Johnson, D. M. (1978): Avowed happiness as an overall assessment of the quality of life. In: *Social Indicators Research* 5 (1-4), S. 475–492. DOI: 10.1007/BF00352944.

Sonnentag, Sabine; Fritz, Charlotte (2007): The Recovery Experience Questionnaire: development and validation of a measure for assessing recuperation and unwinding from work. In: *Journal of occupational health psychology* 12 (3), S. 204–221. DOI: 10.1037/1076-8998.12.3.204.

Spielberger, Charles Donald; Gorsuch, Richard L.; Lushene, Robert E. (1970): STAI manual for the Stait-Trait Anxiety Inventory ("self-evaluation questionnaire"). Palo Alto, CA: Consulting Psychologists Press.

Watson, David; Clark, Lee Anna; Tellegen, Auke (1988): Development and validation of brief measures of positive and negative affect: The PANAS scales. In: *Journal of Personality and Social Psychology* 54 (6), S. 1063–1070. DOI: 10.1037/0022-3514.54.6.1063.

Weiss, Howard M. (2002): Deconstructing job satisfaction. In: *Human Resource Management Review* 12 (2), S. 173–194. DOI: 10.1016/S1053-4822(02)00045-1.

WHO (2020): Definition einer Depression. Online verfügbar unter https://www.euro.who.int/de/health-topics/noncommunicable-diseases/mental-health/news/news/2012/10/depression-in-europe/depression-definition#:~:text=Eine%20Depression%20ist%20eine%20weit,und%20Konzentrationsschw%C3%A4chen%20gekennzeichnet%20sein%20kann.

Wilson, Alexander C.; Mackintosh, Kate; Power, Kevin; Chan, Stella W. Y. (2019): Effectiveness of Self-Compassion Related Therapies: a Systematic Review and Meta-analysis. In: *Mindfulness* 10 (6), S. 979–995. DOI: 10.1007/s12671-018-1037-6.
