# Supplementary material for: Mindfulness-Based and Mindfulness-Informed Interventions at the Workplace: A Systematic Review and Meta-Regression Analysis of RCTs
Source: Mindfulness (N Y). 2023 May 11:1–34. Online ahead of print. doi: 10.1007/s12671-023-02130-7 (PMC10172073; doi:10.1007/s12671-023-02130-7)
Supplement: Supplementary file 4 — Supplementary file4 (DOCX 45 KB) [file 12671_2023_2130_MOESM4_ESM.docx]

**Supplementary Online Material 4: Excluded studies after full-text screening**

Abbott, Jo-Anne M.; Kaldo, Viktor; Klein, Britt; Austin, David; Hamilton, Catherine; Piterman, Leon et al. (2009): A cluster randomised trial of an internet-based intervention program for tinnitus distress in an industrial setting. In: *Cognitive behaviour therapy* 38 (3), S. 162–173. DOI: 10.1080/16506070902763174.

Abbott, Jo-Anne M.; Kaldo, Viktor; Klein, Britt; Austin, David; Hamilton, Catherine; Piterman, Leon et al. (2010): Human values education and mindfulness meditation as a tool for emotional regulation and stress prevention for teachers: an efficiency study – Behavioral Psychology / Psicología Conductual. Online verfügbar unter <https://www.behavioralpsycho.com/product/human-values-education-and-mindfulness-meditation-as-a-tool-for-emotional-regulation-and-stress-prevention-for-teachers-an-efficiency-study/?lang=en>, zuletzt aktualisiert am 30.06.2021, zuletzt geprüft am 30.06.2021.

ACTRN12611000270910 (2011): Acceptance and Commitment Therapy (ACT) to enhance stress resilience in police recruits: a randomized control trial.

ACTRN12617000300370 (2019): The effect of mobile mindfulness meditation on distress in two cohorts: incoming university students and university staff. In: [*http://www.who.int/trialsearch/Trial2.aspx?TrialID=ACTRN12617000300370*](http://www.who.int/trialsearch/Trial2.aspx?TrialID=ACTRN12617000300370). DOI: 10.1002/central/CN-01857256.

Alexopoulos, Evangelos C.; Zisi, Marilena; Manola, Georgia; Darviri, Christina (2014): Short-term effects of a randomized controlled worksite relaxation intervention in Greece. In: *Annals of agricultural and environmental medicine : AAEM* 21 (2), S. 382–387. DOI: 10.5604/1232-1966.1108609.

Allexandre, D.; Neuman, A.; Hunter, J.; Morledge, T.; Roizen, M. (2012): P02.63. Efficacy of an 8-week online mindfulness stress management program in a corporate call center. In: *BMC Complementary and Alternative Medicine* 12 (S1). DOI: 10.1186/1472-6882-12-S1-P119.

Amar, A. D.; Hlupic, Vlatka; Tamwatin, Tanmika (2014): EFFECT OF MEDITATION ON SELF-PERCEPTION OF LEADERSHIP SKILLS: A CONTROL GROUP STUDY OF CEOs. In: *AMPROC* 2014 (1), S. 14282. DOI: 10.5465/ambpp.2014.300.

Ancona, Matthew R.; Mendelson, Tamar (2014): Feasibility and preliminary outcomes of a yoga and mindfulness intervention for school teachers. In: *Advances in School Mental Health Promotion* 7 (3), S. 156–170. DOI: 10.1080/1754730X.2014.920135.

Anderson, Vidya L.; Levinson, Edward M.; Barker, William; Kiewra, Kathleen R. (1999): The effects of meditation on teacher perceived occupational stress, state and trait anxiety, and burnout. In: *School Psychology Quarterly* 14 (1), S. 3–25. DOI: 10.1037/h0088995.

Ando, Michiyo; Natsume, Takako; Kukihara, Hiroko; Shibata, Hiroko; Ito, Sayoko (2011): Efficacy of mindfulness-based meditation therapy on the sense of coherence and mental health of nurses. In: *Health* 03 (02), S. 118–122. DOI: 10.4236/health.2011.32022.

Angerer, P.; Li, J. (2017): Evaluation of short-term and long-term effects of work stress interventions on CVD risk factors: focusing on the individual level.

Aranda Auserón, Gloria; Elcuaz Viscarret, M. Rosario; Fuertes Goñi, Carmen; Güeto Rubio, Victoria; Pascual Pascual, Pablo; Sainz de Murieta García de Galdeano, Enrique (2018): Evaluación de la efectividad de un programa de mindfulness y autocompasión para reducir el estrés y prevenir el burnout en profesionales sanitarios de atención primaria. In: *Atencion primaria* 50 (3), S. 141–150. DOI: 10.1016/j.aprim.2017.03.009.

Arcari, Patricia M. (1996): Efficacy of a workplace smoking cessation program: Mindfulness meditation vs cognitive-behavioral interventions - ProQuest. Online verfügbar unter <https://www.proquest.com/openview/aa649f3de86574344fd569b0519ee1d6/1?cbl=18750&diss=y&pq-origsite=gscholar>, zuletzt aktualisiert am 30.06.2021, zuletzt geprüft am 30.06.2021.

Arora, Sonal; Aggarwal, Rajesh; Moran, Aidan; Sirimanna, Pramudith; Crochet, Patrice; Darzi, Ara et al. (2011): Mental practice: effective stress management training for novice surgeons. In: *Journal of the American College of Surgeons* 212 (2), S. 225–233. DOI: 10.1016/j.jamcollsurg.2010.09.025.

Artemiou, Elpida; Gilbert, Gregory E.; Callanan, Anne; Marchi, Silvia; Bergfelt, Don R. (2018): Mind-body therapies: an intervention to reduce work-related stress in veterinary academia. In: *The Veterinary record* 183 (19), S. 596. DOI: 10.1136/vr.104815.

Bachmann, Katharina; Lam, Alexandra P.; Sörös, Peter; Kanat, Manuela; Hoxhaj, Eliza; Matthies, Swantje et al. (2018): Effects of mindfulness and psychoeducation on working memory in adult ADHD: A randomised, controlled fMRI study. In: *Behaviour research and therapy* 106, S. 47–56. DOI: 10.1016/j.brat.2018.05.002.

Baker, Christine; Huxley, Peter; Dennis, Michael; Islam, Saiful; Russell, Ian (2015): Alleviating staff stress in care homes for people with dementia: protocol for stepped-wedge cluster randomised trial to evaluate a web-based Mindfulness- Stress Reduction course. In: *BMC psychiatry* 15, S. 317. DOI: 10.1186/s12888-015-0703-7.

Barattucci, Massimiliano; Padovan, Anna Maria; Vitale, Ermanno; Rapisarda, Venerando; Ramaci, Tiziana; Giorgio, Andrea de (2019): Mindfulness-Based IARA Model® Proves Effective to Reduce Stress and Anxiety in Health Care Professionals. A Six-Month Follow-Up Study. In: *International journal of environmental research and public health* 16 (22). DOI: 10.3390/ijerph16224421.

Bazzano, Alessandra N.; Anderson, Christopher E.; Hylton, Chelsea; Gustat, Jeanette (2018): Effect of mindfulness and yoga on quality of life for elementary school students and teachers: results of a randomized controlled school-based study. In: *Psychology research and behavior management* 11, S. 81–89. DOI: 10.2147/PRBM.S157503.

Bellad A.S., Goudar S.S. (2021): Effect of yoga on motor performance of upper limbs among laboratory workers - Randomized controlled trial. Online verfügbar unter <https://www.researchgate.net/publication/286989074_Effect_of_yoga_on_motor_performance_of_upper_limbs_among_laboratory_workers_-_Randomized_controlled_trial>, zuletzt aktualisiert am 30.06.2021, zuletzt geprüft am 30.06.2021.

Benn, Rita; Akiva, Tom; Arel, Sari; Roeser, Robert W. (2012): Mindfulness training effects for parents and educators of children with special needs. In: *Developmental psychology* 48 (5), S. 1476–1487. DOI: 10.1037/a0027537.

Beth Israel Deaconess Medical Center (2020): Inner Engineering Online (IEO) Intervention for a Specific Company Employee Program. NCT04126564, 2019P000715. Hg. v. ClinicalTrials.gov. Online verfügbar unter <https://clinicaltrials.gov/ct2/show/NCT04126564>, zuletzt aktualisiert am 04.08.2020, zuletzt geprüft am 30.06.2021.

Blake, Holly; Lai, Betsy; Coman, Emil; Houdmont, Jonathan; Griffiths, Amanda (2019): Move-It: A Cluster-Randomised Digital Worksite Exercise Intervention in China: Outcome and Process Evaluation. In: *International journal of environmental research and public health* 16 (18). DOI: 10.3390/ijerph16183451.

Boccia, Anthony: Reducing individual stress in the workplace through yoga and the strength deployment inventory: An experimental study.

Bostock, S. K.; Steptoe, A. (2021): CAN FINDING HEADSPACE REDUCE WORK STRESS? A RANDOMISED CONTROLLED WORKPLACE TRIAL OF A MINDFULNESS MEDITATION APP | Request PDF. Online verfügbar unter <https://www.researchgate.net/publication/293476630_CAN_FINDING_HEADSPACE_REDUCE_WORK_STRESS_A_RANDOMISED_CONTROLLED_WORKPLACE_TRIAL_OF_A_MINDFULNESS_MEDITATION_APP>, zuletzt aktualisiert am 30.06.2021, zuletzt geprüft am 30.06.2021.

Brendel, William; Hankerson, Sarah; Byun, Sangwon; Cunningham, Birdie (2016): Cultivating leadership Dharma. In: *Journal of Mgmt Development* 35 (8), S. 1056–1078. DOI: 10.1108/JMD-09-2015-0127.

Canterbury Christ Church University; Sussex Partnership NHS Foundation Trust; University of Sussex (2019): A Randomised Controlled Trial of a Mindfulness-based Cognitive Therapy Self-help Intervention for UK National Health Service Employees. NCT03030040, EmilyIronmongerMRP2016. Hg. v. ClinicalTrials.gov. Online verfügbar unter <https://clinicaltrials.gov/ct2/show/NCT03030040>, zuletzt aktualisiert am 22.03.2019, zuletzt geprüft am 30.06.2021.

Carvalho, Joana Sampaio de; Pinto, Alexandra Marques; Marôco, João (2017): Results of a Mindfulness-Based Social-Emotional Learning Program on Portuguese Elementary Students and Teachers: a Quasi-Experimental Study. In: *Mindfulness* 8 (2), S. 337–350. DOI: 10.1007/s12671-016-0603-z.

Chan, J.; Singh, N. (2016): Reducing 1: 1 observations through mindfulness-based positive behaviour supports training for caregivers.

Cheema, Birinder S.; Houridis, Angelique; Busch, Lisa; Raschke-Cheema, Verena; Melville, Geoff W.; Marshall, Paul W. et al. (2013): Effect of an office worksite-based yoga program on heart rate variability: outcomes of a randomized controlled trial. In: *BMC Complementary and Alternative Medicine* 13, S. 82. DOI: 10.1186/1472-6882-13-82.

Chokkalingam; Kumari, Sony; Akhilesh, K. B.; Nagendra, H. R. (2015): Effect of Integrated Yoga on Emotional Stability and Performance of Employees: An Action Research Study. In: *Prabandhan: Indian Journal of Management* 8 (8), S. 7. DOI: 10.17010/pijom/2015/v8i8/75049.

Christakis, Ioannis; Pagkratis, Marios T.; Varvogli, Lisa; Darviri, Christina; Chroussos, George (2012): Measuring the stress of the surgeons in training and use of a novel interventional program to combat it. In: *Journal of the Korean Surgical Society* 82 (5), S. 312–316. DOI: 10.4174/jkss.2012.82.5.312.

Christopher, Michael S.; Hunsinger, Matthew; Goerling, Lt Richard J.; Bowen, Sarah; Rogers, Brant S.; Gross, Cynthia R. et al. (2018): Mindfulness-based resilience training to reduce health risk, stress reactivity, and aggression among law enforcement officers: A feasibility and preliminary efficacy trial. In: *Psychiatry research* 264, S. 104–115. DOI: 10.1016/j.psychres.2018.03.059.

Cohen-Katz, Joanne; Wiley, Susan D.; Capuano, Terry; Baker, Debra M.; Kimmel, Sharon; Shapiro, Shauna (2005): The effects of mindfulness-based stress reduction on nurse stress and burnout, Part II: A quantitative and qualitative study. In: *Holistic nursing practice* 19 (1), S. 26–35. DOI: 10.1097/00004650-200501000-00008.

Cooley, Kieran; Szczurko, Orest; Perri, Dan; Mills, Edward J.; Bernhardt, Bob; Zhou, Qi; Seely, Dugald (2009): Naturopathic care for anxiety: a randomized controlled trial ISRCTN78958974. In: *PloS one* 4 (8), e6628. DOI: 10.1371/journal.pone.0006628.

CTRI/2018/02/012206 (2018): Effect of yoga on stress among nursing staff in a tertiary care hospital.

Deady, M.; Johnston, D. A.; Glozier, N.; Milne, D.; Choi, I.; Mackinnon, A. et al. (2018): A smartphone application for treating depressive symptoms: study protocol for a randomised controlled trial. In: *BMC psychiatry* 18 (1), S. 166. DOI: 10.1186/s12888-018-1752-5.

Dreusicke, M.; Ruff, K. M.; Wolever, R. (2016): Mindfulness program delivered virtually in the workplace shows correlation between decreased stress and increased productivity.

DRKS00014015 (2018): Muße im Krankenhaus? Eine achtsamkeitsbasierte Intervention bei AssistenzärztInnen. Online verfügbar unter <https://www.drks.de/drks_web/navigate.do?navigationId=trial.HTML&TRIAL_ID=DRKS00014015>, zuletzt aktualisiert am 30.06.2021, zuletzt geprüft am 30.06.2021.

Duarte, Joana; Pinto-Gouveia, José (2017): Mindfulness, self-compassion and psychological inflexibility mediate the effects of a mindfulness-based intervention in a sample of oncology nurses. In: *Journal of Contextual Behavioral Science* 6 (2), S. 125–133. DOI: 10.1016/j.jcbs.2017.03.002.

Dunne, Pádraic J.; Lynch, Julie; Prihodova, Lucia; O’Leary, Caoimhe; Ghoreyshi, Atiyeh; Basdeo, Sharee A. et al. (2019): Burnout in the emergency department: Randomized controlled trial of an attention-based training program. In: *Journal of integrative medicine* 17 (3), S. 173–180. DOI: 10.1016/j.joim.2019.03.009.

Dwivedi, Umesh; Kumari, Sony; Akhilesh, K. B.; Nagendra, H. R. (2015): Effect of Yoga Practices in Reducing Aggression and Counterproductive Work Behavior: A Randomized Controlled Trial. In: *Prabandhan: Indian Journal of Management* 8 (10), S. 21. DOI: 10.17010/pijom/2015/v8i10/79822.

Feicht, T.; Wittmann, M.; Jose, G.; Mock, A.; Hirschhausen, E. von; Esch, T. (2013): Evaluation of a seven-week web-based happiness training to improve psychological well-being, reduce stress, and enhance mindfulness and flourishing: a randomized controlled occupational health study. In: *Evidence-based complementary and alternative medicine : eCAM* 2013, S. 676953. DOI: 10.1155/2013/676953.

Frank, Jennifer L.; Reibel, Diane; Broderick, Patricia; Cantrell, Todd; Metz, Stacie (2015): The Effectiveness of Mindfulness-Based Stress Reduction on Educator Stress and Well-Being: Results from a Pilot Study. In: *Mindfulness* 6 (2), S. 208–216. DOI: 10.1007/s12671-013-0246-2.

Fredrickson, Barbara L.; Cohn, Michael A.; Coffey, Kimberly A.; Pek, Jolynn; Finkel, Sandra M. (2008): Open hearts build lives: positive emotions, induced through loving-kindness meditation, build consequential personal resources. In: *Journal of personality and social psychology* 95 (5), S. 1045–1062. DOI: 10.1037/a0013262.

Gilmartin, Heather; Saint, Sanjay; Rogers, Mary; Winter, Suzanne; Snyder, Ashley; Quinn, Martha; Chopra, Vineet (2018): Pilot randomised controlled trial to improve hand hygiene through mindful moments. In: *BMJ quality & safety* 27 (10), S. 799–806. DOI: 10.1136/bmjqs-2017-007359.

Griffith, Jay M.; Hasley, Joseph P.; Liu, Hong; Severn, Daniel G.; Conner, Latoya H.; Adler, Lawrence E. (2008): Qigong stress reduction in hospital staff. In: *Journal of Alternative and Complementary Medicine* 14 (8), S. 939–945. DOI: 10.1089/acm.2007.0814.

H. Toivanen; E. Länsimies; O. Hänninen (1993): Sympathovagal interaction in stress and relaxation; heart rate variability in home nurses. Online verfügbar unter <https://www.semanticscholar.org/paper/Sympathovagal-interaction-in-stress-and-relaxation%3B-Toivanen-L%C3%A4nsimies/4c5786d445af831542248a5014ff8c0a12ca10f6>.

Hallquist, Rachel A. (2018): New teachers and wellbeing.

Hamilton-West, Kate; Pellatt-Higgins, Tracy; Pillai, Neil (2018): Does a modified mindfulness-based cognitive therapy (MBCT) course have the potential to reduce stress and burnout in NHS GPs? Feasibility study. In: *Primary health care research & development* 19 (6), S. 591–597. DOI: 10.1017/S1463423618000129.

Harris, Alexis R.; Jennings, Patricia A.; Katz, Deirdre A.; Abenavoli, Rachel M.; Greenberg, Mark T. (2016): Promoting Stress Management and Wellbeing in Educators: Feasibility and Efficacy of a School-Based Yoga and Mindfulness Intervention. In: *Mindfulness* 7 (1), S. 143–154. DOI: 10.1007/s12671-015-0451-2.

Harris, Alexis Rae (2014): Promoting educators’ social-emotional competence, stress management, and wellbeing through a school-based contemplative intervention: An evaluation of efficacy, implementation process, and mechanisms. Online verfügbar unter <https://etda.libraries.psu.edu/catalog/22415>.

Hoge, E.; Bui, T. H.E.; Metcalf, C.; Pollack, M. H.; Simon, N. M. (2012): Mindfulness training improves resilience: reductions in adrenocorticotropic hormone (ACTH) response to laboratory stress.

Hue, Ming-tak; Lau, Ngar-sze (2015): Promoting well-being and preventing burnout in teacher education: a pilot study of a mindfulness-based programme for pre-service teachers in Hong Kong. In: *Teacher Development* 19 (3), S. 381–401. DOI: 10.1080/13664530.2015.1049748.

Hwang, Yoon-Suk; Goldstein, Harvey; Medvedev, Oleg N.; Singh, Nirbhay N.; Noh, Jae-Eun; Hand, Kirstine (2019): Mindfulness-Based Intervention for Educators: Effects of a School-Based Cluster Randomized Controlled Study. In: *Mindfulness* 10 (7), S. 1417–1436. DOI: 10.1007/s12671-019-01147-1.

Jay, Kenneth; Brandt, Mikkel; Hansen, Klaus; Sundstrup, Emil; Jakobsen, Markus D.; Schraefel, M. C. et al. (2015): Effect of Individually Tailored Biopsychosocial Workplace Interventions on Chronic Musculoskeletal Pain and Stress Among Laboratory Technicians: Randomized Controlled Trial. In: *Pain physician* 18 (5), S. 459–471.

Jay, Kenneth; Brandt, Mikkel; Jakobsen, Markus Due; Sundstrup, Emil; Berthelsen, Kasper Gymoese; Schraefel, Mc et al. (2016): Ten weeks of physical-cognitive-mindfulness training reduces fear-avoidance beliefs about work-related activity: Randomized controlled trial. In: *Medicine* 95 (34), e3945. DOI: 10.1097/MD.0000000000003945.

Jennings, Patricia A.; Snowberg, Karin E.; Coccia, Michael A.; Greenberg, Mark T. (2011): Improving Classroom Learning Environments by Cultivating Awareness and Resilience in Education (CARE): Results of Two Pilot Studies. In: *The Journal of Classroom Interaction* 46 (1), S. 37–48. Online verfügbar unter <http://www.jstor.org/stable/23870550>.

Jha, Amishi P.; Morrison, Alexandra B.; Dainer-Best, Justin; Parker, Suzanne; Rostrup, Nina; Stanley, Elizabeth A. (2015): Minds “at attention”: mindfulness training curbs attentional lapses in military cohorts. In: *PloS one* 10 (2), e0116889. DOI: 10.1371/journal.pone.0116889.

Johnson, Douglas C.; Thom, Nathaniel J.; Stanley, Elizabeth A.; Haase, Lori; Simmons, Alan N.; Shih, Pei-An B. et al. (2014): Modifying resilience mechanisms in at-risk individuals: a controlled study of mindfulness training in Marines preparing for deployment. In: *The American journal of psychiatry* 171 (8), S. 844–853. DOI: 10.1176/appi.ajp.2014.13040502.

Josefsson, Torbjörn; Lindwall, Magnus; Broberg, Anders G. (2014): The Effects of a Short-term Mindfulness Based Intervention on Self-reported Mindfulness, Decentering, Executive Attention, Psychological Health, and Coping Style: Examining Unique Mindfulness Effects and Mediators. In: *Mindfulness* 5 (1), S. 18–35. DOI: 10.1007/s12671-012-0142-1.

Joshi, Vidya (2017): Nerve muscle physiology changes with yoga in professional computer users. In: *Natl J Physiol Pharm Pharmacol*, S. 1. DOI: 10.5455/njppp.2017.7.0410002042017.

Joshi, Vidya S.; Bellad, Anjana S. (2011): Effect of yogic exercises on symptoms of musculoskeletal disorders of upper limbs among computer users: a randomised controlled trial. In: *Indian journal of medical sciences* 65 (10), S. 424–428. DOI: 10.4103/0019-5359.109256.

Joyce, Sadhbh; Shand, Fiona; Lal, Tara J.; Mott, Brendan; Bryant, Richard A.; Harvey, Samuel B. (2019): Resilience@Work Mindfulness Program: Results From a Cluster Randomized Controlled Trial With First Responders. In: *Journal of medical Internet research* 21 (2), e12894. DOI: 10.2196/12894.

Justo, Franco (2010): Mindfulness program for increasing resilience and preventing burnouts in secondary school teachers. Online verfügbar unter <https://www.researchgate.net/publication/289202474_Mindfulness_program_for_increasing_resilience_and_preventing_burnouts_in_secondary_school_teachers>, zuletzt aktualisiert am 30.06.2021, zuletzt geprüft am 30.06.2021.

Kao, Henry; Zhu, Lin; Chao, An an; Chen, Hao Yi; Liu, Ivy Cy; Zhang, Manlin (2014): Calligraphy and meditation for stress reduction: an experimental comparison. In: *Psychology research and behavior management* 7, S. 47–52. DOI: 10.2147/PRBM.S55743.

Kaplan, Seth; Bradley-Geist, Jill C.; Ahmad, Afra; Anderson, Amanda; Hargrove, Amber K.; Lindsey, Alex (2014): A Test of Two Positive Psychology Interventions to Increase Employee Well-Being. In: *J Bus Psychol* 29 (3), S. 367–380. DOI: 10.1007/s10869-013-9319-4.

Kemper, Kathi J. (2017): Brief Online Mindfulness Training: Immediate Impact. In: *Journal of Evidence-based Complementary & Alternative Medicine* 22 (1), S. 75–80. DOI: 10.1177/2156587216639199.

Kemper, Kathi J.; Khirallah, Michael (2015): Acute Effects of Online Mind-Body Skills Training on Resilience, Mindfulness, and Empathy. In: *Journal of Evidence-based Complementary & Alternative Medicine* 20 (4), S. 247–253. DOI: 10.1177/2156587215575816.

Kim, Johanna Inyang; Yun, Je-Yeon; Park, Heyeon; Park, Suk-Young; Ahn, Youngsheen; Lee, Hansol et al. (2018): A Mobile Videoconference-Based Intervention on Stress Reduction and Resilience Enhancement in Employees: Randomized Controlled Trial. In: *Journal of medical Internet research* 20 (10), e10760. DOI: 10.2196/10760.

Kirk, Ulrich; Gu, Xiaosi; Sharp, Carla; Hula, Andreas; Fonagy, Peter; Montague, P. Read (2016): Mindfulness training increases cooperative decision making in economic exchanges: Evidence from fMRI. In: *Neuroimage* 138, S. 274–283. DOI: 10.1016/j.neuroimage.2016.05.075.

Klatt, M.; Steinberg, B.; Marks, D.; Duchemin, A. (2012): OA04.04. Changes in physiological and psychological markers of stress in hospital personnel after a low-dose mindfulness-based worksite intervention. In: *BMC Complementary and Alternative Medicine* 12 (S1). DOI: 10.1186/1472-6882-12-S1-O16.

Kuhlmann, S. M.; Huss, M.; Bürger, A.; Hammerle, F. (2016): Coping with stress in medical students: results of a randomized controlled trial using a mindfulness-based stress prevention training (MediMind) in Germany. In: *BMC medical education* 16 (1), S. 316. DOI: 10.1186/s12909-016-0833-8.

Lahn, Molly J.: Indices of heart rate variability and compassion in healthcare professionals following stress resilience training.

Li, Bo; Li, Xin-xin; Ma, Chang-yan (2013): The effects of mindfulness-based group training on improving employees’ body and mind conditions. [The effects of mindfulness-based group training on improving employees’ body and mind conditions.].

Loiselle, Marie E. (2018): Academic Physician Burnout and Transcendental Meditation: A Mixed Methods Randomized Controlled Trial - ProQuest. Online verfügbar unter <https://www.proquest.com/openview/b0954e8cf2dbf56c16050796c3978fa4/1?pq-origsite=gscholar&cbl=18750>, zuletzt aktualisiert am 30.06.2021, zuletzt geprüft am 30.06.2021.

Lui, Wai Sze: A randomized controlled trial study to alleviate healthcare workers’ burnout and perceived stress by mindful practice program.

Mache, Stefanie; Vitzthum, Karin; Klapp, Burghard F.; Groneberg, David A. (2015): Evaluation of a Multicomponent Psychosocial Skill Training Program for Junior Physicians in Their First Year at Work: A Pilot Study. In: *Family medicine* 47 (9), S. 693–698.

Mak, Winnie W. S.; Chan, Amy T. Y.; Cheung, Eliza Y. L.; Lin, Cherry L. Y.; Ngai, Karin C. S. (2015): Enhancing Web-based mindfulness training for mental health promotion with the health action process approach: randomized controlled trial. In: *Journal of medical Internet research* 17 (1), e8. DOI: 10.2196/jmir.3746.

Malarkey, William B.; Jarjoura, David; Klatt, Maryanna (2013): Workplace based mindfulness practice and inflammation: a randomized trial. In: *Brain, behavior, and immunity* 27 (1), S. 145–154. DOI: 10.1016/j.bbi.2012.10.009.

Manas, Israel; Franco, Clemente; Martinez, Eduardo J. (2011): Reducing Levels of Teacher Stress and the Days of Sick Leave in Secondary School Teachers through a Mindfulness Training Programme | Request PDF. Online verfügbar unter <https://www.researchgate.net/publication/317481370_Reducing_Levels_of_Teacher_Stress_and_the_Days_of_Sick_Leave_in_Secondary_School_Teachers_through_a_Mindfulness_Training_Programme>, zuletzt aktualisiert am 30.06.2021, zuletzt geprüft am 30.06.2021.

Mander, Johannes; Kröger, Paula; Heidenreich, Thomas; Flückiger, Christoph; Lutz, Wolfgang; Bents, Hinrich; Barnow, Sven (2015): The Process-Outcome Mindfulness Effects in Trainees (PrOMET) study: protocol of a pragmatic randomized controlled trial. In: *BMC psychology* 3 (1), S. 25. DOI: 10.1186/s40359-015-0082-3.

Manocha, R. (2011): FP03-3 A Randomised Trial of Mental Silence Orientated Meditation for Work-related Stress, Anxiety and Depressive Feelings. In: *Asian Journal of Psychiatry* 4, S31. DOI: 10.1016/S1876-2018(11)60122-6.

Manocha, R.; Black, D.; Sarris, J.; Stough, C. (2011): A randomized, controlled trial of meditation for work stress, anxiety and depressed mood in full-time workers. In: *Evidence-based complementary and alternative medicine : eCAM* 2011, S. 960583. DOI: 10.1155/2011/960583.

Manotas, Manuel Andres (2012): Brief mindfulness training to improve mental health with colombian healthcare professionals.

McConachie, Douglas Alexander James; McKenzie, Karen; Morris, Paul Graham; Walley, Robert M. (2014): Acceptance and mindfulness-based stress management for support staff caring for individuals with intellectual disabilities. In: *Research in developmental disabilities* 35 (6), S. 1216–1227. DOI: 10.1016/j.ridd.2014.03.005.

McElligott, Deborah; Holz, Mary Beth; Carollo, Laurie; Somerville, Susan; Baggett, Margarita; Kuzniewski, Sally; Shi, Qiuhu (2003): A pilot feasibility study of the effects of touch therapy on nurses. In: *The Journal of the New York State Nurses’ Association* 34 (1), S. 16–24.

Mealer, M.; Conrad, D.; Evans, J.; Jooste, K.; Rothbaum, B.; Moss, M. (2014a): A 12-Week Multimodal Resilience Training Program For Intensive Care Unit Nurses: Results Of A Pilot Study | C106. INTERPROFESSIONAL TOPICS IN THE PROVISION OF CRITICAL CARE, zuletzt aktualisiert am 30.06.2021, zuletzt geprüft am 30.06.2021.

Mealer, Meredith; Conrad, David; Evans, John; Jooste, Karen; Solyntjes, Janet; Rothbaum, Barbara; Moss, Marc (2014b): Feasibility and acceptability of a resilience training program for intensive care unit nurses. In: *American journal of critical care : an official publication, American Association of Critical-Care Nurses* 23 (6), e97-105. DOI: 10.4037/ajcc2014747.

Molina, Agustin; O’Shea, Deirdre (2020): Mindful Emotion Regulation, Savouring and Proactive Behaviour: The Role of Supervisor Justice. In: *Applied Psychology* 69 (1), S. 148–175. DOI: 10.1111/apps.12206.

Moody, Karen; Kramer, Deborah; Santizo, Ruth O.; Magro, Laurence; Wyshogrod, Diane; Ambrosio, John et al. (2013): Helping the helpers: mindfulness training for burnout in pediatric oncology—a pilot program. In: *Journal of pediatric oncology nursing : official journal of the Association of Pediatric Oncology Nurses* 30 (5), S. 275–284. DOI: 10.1177/1043454213504497.

Morrison Wylde, Chelsey; Mahrer, Nicole E.; Meyer, Rika M. L.; Gold, Jeffrey I. (2017): Mindfulness for Novice Pediatric Nurses: Smartphone Application Versus Traditional Intervention. In: *Journal of pediatric nursing* 36, S. 205–212. DOI: 10.1016/j.pedn.2017.06.008.

NCT03097510 (2017): Meditation and Emotional Intelligence.

NCT04038658 (2019): Digital Worksite Exercise Intervention in China: outcome and Process Evaluation.

Nedeljkovic, Marko; Ausfeld-Hafter, Brigitte; Seiler, Roland; Wirtz, Petra H. (2012): Minderung physiologischer Reaktivität auf psychosozialen Stress durch Taiji-Training - wer profitiert besonders? Eine Untersuchung moderierender Effekte von selbstberichteter dispositioneller psychologischer Stressreaktivität und Achtsamkeit.

Netterstrøm, Bo; Friebel, Lene; Ladegaard, Yun (2013): Effects of a multidisciplinary stress treatment programme on patient return to work rate and symptom reduction: results from a randomised, wait-list controlled trial. In: *Psychotherapy and psychosomatics* 82 (3), S. 177–186. DOI: 10.1159/000346369.

Oman, Doug; Hedberg, John; Thoresen, Carl E. (2006): Passage meditation reduces perceived stress in health professionals: a randomized, controlled trial. In: *Journal of consulting and clinical psychology* 74 (4), S. 714–719. DOI: 10.1037/0022-006X.74.4.714.

Oman, Doug; Thoresen, Carl E.; Hedberg, John (2010): Does passage meditation foster compassionate love among health professionals?: a randomised trial. In: *Mental Health, Religion & Culture* 13 (2), S. 129–154. DOI: 10.1080/13674670903261954.

Page, Kathryn M.; Vella-Brodrick, Dianne A. (2013): The Working for Wellness Program: RCT of an Employee Well-Being Intervention. In: *J Happiness Stud* 14 (3), S. 1007–1031. DOI: 10.1007/s10902-012-9366-y.

Palumbo, Mary Val; Wu, Ge; Shaner-McRae, Hollie; Rambur, Betty; McIntosh, Barbara (2012): Tai Chi for older nurses: a workplace wellness pilot study. In: *Applied Nursing Research* 25 (1), S. 54–59. DOI: 10.1016/j.apnr.2010.01.002.

Patient Safety Translational Research Centre (2021): An Acceptance and Commitment Therapy-based workplace intervention for improving wellbeing of NHS staff. Online verfügbar unter <https://yhpstrc.org/research-themes-partners/workforce-engagement-and-wellbeing/our-projects/act/>, zuletzt aktualisiert am 30.06.2021, zuletzt geprüft am 30.06.2021.

Pauls, Nina; Schlett, Christian; Soucek, Roman; Ziegler, Michael; Frank, Nicole (2016): Resilienz durch Training personaler Ressourcen stärken: Evaluation einer web-basierten Achtsamkeitsintervention. In: *Gr Interakt Org* 47 (2), S. 105–117. DOI: 10.1007/s11612-016-0315-9.

Pérula-de Torres, Luis-Angel; Atalaya, Juan Carlos Verdes-Montenegro; García-Campayo, Javier; Roldán-Villalobos, Ana; Magallón-Botaya, Rosa; Bartolomé-Moreno, Cruz et al. (2019): Controlled clinical trial comparing the effectiveness of a mindfulness and self-compassion 4-session programme versus an 8-session programme to reduce work stress and burnout in family and community medicine physicians and nurses: MINDUUDD study protocol. In: *BMC family practice* 20 (1), S. 24. DOI: 10.1186/s12875-019-0913-z.

Pidgeon, Aileen M.; Ford, Lucas; Klaassen, Frances (2014): Evaluating the effectiveness of enhancing resilience in human service professionals using a retreat-based Mindfulness with Metta Training Program: a randomised control trial. In: *Psychology, health & medicine* 19 (3), S. 355–364. DOI: 10.1080/13548506.2013.806815.

Pomykala, Kelsey L.; Silverman, Daniel Hs; Geist, Cheri L.; Voege, Patricia; Siddarth, Prabha; Nazarian, Nora et al. (2012): A pilot study of the effects of meditation on regional brain metabolism in distressed dementia caregivers. In: *Aging health* 8 (5), S. 509–516. DOI: 10.2217/ahe.12.46.

Prasek, Aimee: Randomized Controlled Trial to Evaluate a Self-Guided, Web-Based Mindfulness Program for Stress Reduction and Wellbeing Promotion. Online verfügbar unter <https://conservancy.umn.edu/handle/11299/175304>.

Rachiwong, S.; Panasiriwong, P.; Saosomphop, J.; Widjaja, W.; Ajjimaporn, A. (2015): Effects of Modified Hatha Yoga in Industrial Rehabilitation on Physical Fitness and Stress of Injured Workers. In: *Journal of occupational rehabilitation* 25 (3), S. 669–674. DOI: 10.1007/s10926-015-9574-5.

Ramsey, Alex T.; Jones, Eric E. (2015): Minding the interpersonal gap: mindfulness-based interventions in the prevention of ostracism. In: *Consciousness and cognition* 31, S. 24–34. DOI: 10.1016/j.concog.2014.10.003.

Ranta, Randhir (2012): Stress and anger management among police personnel through Indian psychological techniques | Request PDF. Online verfügbar unter <https://www.researchgate.net/publication/288653028_Stress_and_anger_management_among_police_personnel_through_Indian_psychological_techniques>, zuletzt aktualisiert am 30.06.2021, zuletzt geprüft am 30.06.2021.

Ranta, Randhir Singh (2009): Management of stress and coping behaviour of police personnel through Indian psychological techniques. - PsycNET. Online verfügbar unter <https://psycnet.apa.org/record/2009-01888-006>, zuletzt aktualisiert am 16.06.2021, zuletzt geprüft am 30.06.2021.

Sadeghnia, Alireza; Danaei, Navid; Barkatein, Behzad (2016): A Comparison of the Effect of Nasal bi-level Positive Airway Pressure and Sigh-positive Airway Pressure on the Treatment of the Preterm Newborns Weighing Less than 1500 g Affiliated with Respiratory Distress Syndrome. In: *International journal of preventive medicine* 7, S. 21. DOI: 10.4103/2008-7802.173930.

Schmid, Arlene A.; van Puymbroeck, Marieke; Fruhauf, Christine A.; Bair, Matthew J.; Portz, Jennifer Dickman (2019): Yoga improves occupational performance, depression, and daily activities for people with chronic pain. In: *Work (Reading, Mass.)* 63 (2), S. 181–189. DOI: 10.3233/WOR-192919.

Shapiro, Shauna L.; Astin, John A.; Bishop, Scott R.; Cordova, Matthew (2005): Mindfulness-Based Stress Reduction for Health Care Professionals: Results From a Randomized Trial. In: *International Journal of Stress Management* 12 (2), S. 164–176. DOI: 10.1037/1072-5245.12.2.164.

Shapiro, Shauna L.; Astin, John A.; Bishop, Scott R.; Cordova, Matthew (2021): Mindfulness-Based Stress Reduction for Health Care Professionals: Results From a Randomized Trial. - PsycNET. Online verfügbar unter <https://psycnet.apa.org/record/2005-05099-004>, zuletzt aktualisiert am 16.06.2021, zuletzt geprüft am 30.06.2021.

Shete, Sanjay Uddhav; Verma, Anita; Kulkarni, Dattatraya Devarao; Bhogal, Ranjeet Singh (2017): Effect of yoga training on inflammatory cytokines and C-reactive protein in employees of small-scale industries. In: *Journal of education and health promotion* 6, S. 76. DOI: 10.4103/jehp.jehp_65_17.

Shonin, Edo; van Gordon, William (2015): Managers’ Experiences of Meditation Awareness Training. In: *Mindfulness* 6 (4), S. 899–909. DOI: 10.1007/s12671-014-0334-y.

Siedsma, Matthew; Emlet, Lillian (2015): Physician burnout: can we make a difference together? In: *Critical care (London, England)* 19, S. 273. DOI: 10.1186/s13054-015-0990-x.

Simon, Al; Harnett, Susanne; Nagler, Eden; Thomas, Latisha (2010): Research on the Effect of the Inner Resilience Program on Teacher and Student Wellness and Classroom Climate I. Online verfügbar unter <https://resilienceorg.files.wordpress.com/2017/07/irp-evaluation-finalexecutivesummary-feb-2010.pdf>, zuletzt geprüft am 30.06.2021.

Steinberg, Beth; Bartimole, Lucy; Habash, Diane; Fristad, Mary A. (2017): Tai Chi for Workplace Wellness: Pilot Feasibility Study. In: *Explore (New York, N.Y.)* 13 (6), S. 407–408. DOI: 10.1016/j.explore.2016.12.017.

Strijk, Jorien E.; Proper, Karin I.; van der Beek, Allard J.; van Mechelen, Willem (2009): The Vital@Work Study. The systematic development of a lifestyle intervention to improve older workers’ vitality and the design of a randomised controlled trial evaluating this intervention. In: *BMC public health* 9, S. 408. DOI: 10.1186/1471-2458-9-408.

Strijk, Jorien E.; Proper, Karin I.; van der Beek, Allard J.; van Mechelen, Willem (2011): A process evaluation of a worksite vitality intervention among ageing hospital workers. In: *Int J Behav Nutr Phys Act* 8 (1), S. 58. DOI: 10.1186/1479-5868-8-58.

Strijk, Jorien E.; Proper, Karin I.; van der Beek, Allard J.; van Mechelen, Willem (2012): A worksite vitality intervention to improve older workers’ lifestyle and vitality-related outcomes: results of a randomised controlled trial. In: *Journal of epidemiology and community health* 66 (11), S. 1071–1078. DOI: 10.1136/jech-2011-200626.

Strijk, Jorien E.; Proper, Karin I.; van Mechelen, Willem; van der Beek, Allard J. (2013): Effectiveness of a worksite lifestyle intervention on vitality, work engagement, productivity, and sick leave: results of a randomized controlled trial. In: *Scandinavian journal of work, environment & health* 39 (1), S. 66–75. DOI: 10.5271/sjweh.3311.

Szczurko, Orest; Cooley, Kieran; Busse, Jason W.; Seely, Dugald; Bernhardt, Bob; Guyatt, Gordon H. et al. (2007): Naturopathic care for chronic low back pain: a randomized trial. In: *PloS one* 2 (9), e919. DOI: 10.1371/journal.pone.0000919.

Taylor, Cynthia; Harrison, Jessica; Haimovitz, Kyla; Oberle, Eva; Thomson, Kimberly; Schonert-Reichl, Kimberly; Roeser, Robert W. (2016): Examining Ways That a Mindfulness-Based Intervention Reduces Stress in Public School Teachers: a Mixed-Methods Study. In: *Mindfulness* 7 (1), S. 115–129. DOI: 10.1007/s12671-015-0425-4.

Telles, Shirley; Dash, Manoj; Naveen, K. V. (2009): Effect of yoga on musculoskeletal discomfort and motor functions in professional computer users. In: *Work (Reading, Mass.)* 33 (3), S. 297–306. DOI: 10.3233/WOR-2009-0877.

The PLOS ONE Staff (2015): Correction: Effectiveness of a worksite mindfulness-related multi-component health promotion intervention on work engagement and mental health: results of a randomized controlled trial. In: *PLOS ONE* 10 (3), e0122428. DOI: 10.1371/journal.pone.0122428.

The University of Hong Kong; Lok Sin Tong Benevolent Society, Kowloon (2016): Mindfulness Training for Smoking Cessation in Women in the Workplace - a Pilot Randomized Controlled Trial. NCT02497339, LSTWomen. Hg. v. ClinicalTrials.gov. Online verfügbar unter <https://clinicaltrials.gov/ct2/show/NCT02497339>, zuletzt aktualisiert am 12.05.2016, zuletzt geprüft am 30.06.2021.

Todd, Charlotte; Cooksey, Roxanne; Davies, Helen; McRobbie, Clare; Brophy, Sinead (2019): Mixed-methods evaluation comparing the impact of two different mindfulness approaches on stress, anxiety and depression in school teachers. In: *BMJ open* 9 (7), e025686. DOI: 10.1136/bmjopen-2018-025686.

Toivanen, H.; Länsimies, E.; Jokela, V.; Hänninen, O. (1993): Impact of regular relaxation training on the cardiac autonomic nervous system of hospital cleaners and bank employees. In: *Scandinavian journal of work, environment & health* 19 (5), S. 319–325. DOI: 10.5271/sjweh.1468.

Travis, Fred; Valosek, Laurent; Konrad, Arthur; Link, Janice; Salerno, John; Scheller, Ray; Nidich, Sanford (2018): Effect of meditation on psychological distress and brain functioning: A randomized controlled study. In: *Brain and cognition* 125, S. 100–105. DOI: 10.1016/j.bandc.2018.03.011.

Trent, Natalie L.; Borden, Sara; Miraglia, Mindy; Pasalis, Edi; Dusek, Jeffery A.; Khalsa, Sat Bir Singh (2019): Improvements in Psychological and Occupational Well-Being in a Pragmatic Controlled Trial of a Yoga-Based Program for Professionals. In: *Journal of Alternative and Complementary Medicine* 25 (6), S. 593–605. DOI: 10.1089/acm.2018.0526.

Trombka, Marcelo; Demarzo, Marcelo; Bacas, Daniel Campos; Antonio, Sonia Beira; Cicuto, Karen; Salvo, Vera et al. (2018): Study protocol of a multicenter randomized controlled trial of mindfulness training to reduce burnout and promote quality of life in police officers: the POLICE study. In: *BMC psychiatry* 18 (1), S. 151. DOI: 10.1186/s12888-018-1726-7.

Tsai, S. L.; Crockett, M. S. (1993): Effects of relaxation training, combining imagery, and meditation on the stress level of Chinese nurses working in modern hospitals in Taiwan. In: *Issues in mental health nursing* 14 (1), S. 51–66. DOI: 10.3109/01612849309006890.

Tsang, Hector W. H.; Cheung, W. M.; Chan, Alan H. L.; Fung, Kelvin M. T.; Leung, Ada Y.; Au, Doreen W. H. (2015): A pilot evaluation on a stress management programme using a combined approach of cognitive behavioural therapy (CBT) and complementary and alternative medicine (CAM) for elementary school teachers. In: *Stress and health : journal of the International Society for the Investigation of Stress* 31 (1), S. 35–43. DOI: 10.1002/smi.2522.

University College, London; British Heart Foundation; Headspace Meditation Limited; Technical University Dresden (2012): A Randomised Controlled Trial to Investigate the Effects of the Headspace Mindfulness Smartphone App on Psychological Well-being and Biological Indicators of Stress in the Workplace. NCT01661569, 3035/002. Hg. v. ClinicalTrials.gov. Online verfügbar unter <https://clinicaltrials.gov/ct2/show/NCT01661569>, zuletzt aktualisiert am 09.08.2012, zuletzt geprüft am 30.06.2021.

Valley, Morgan Anne: Feasibility of a mindfulness-based stress reduction intervention on health care safety. Feasibility of a mindfulness-based stress reduction intervention on health care safety. Colorado State University. Libraries; Colorado State University. Online verfügbar unter <https://mountainscholar.org/handle/10217/176720?show=full>.

van Berkel, Jantien; Boot, Cécile R. L.; Proper, Karin I.; Bongers, Paulien M.; van der Beek, Allard J. (2014): Effectiveness of a worksite mindfulness-related multi-component health promotion intervention on work engagement and mental health: results of a randomized controlled trial. In: *PloS one* 9 (1), e84118. DOI: 10.1371/journal.pone.0084118.

van Berkel, Jantien; Proper, Karin I.; Boot, Cécile R. L.; Bongers, Paulien M.; van der Beek, Allard J. (2011): Mindful “Vitality in Practice”: an intervention to improve the work engagement and energy balance among workers; the development and design of the randomised controlled trial. In: *BMC public health* 11, S. 736. DOI: 10.1186/1471-2458-11-736.

van Dongen, Johanna M.; Strijk, Jorien E.; Proper, Karin I.; van Wier, Marieke F.; van Mechelen, Willem; van Tulder, Maurits W.; van der Beek, Allard J. (2013): A cost-effectiveness and return-on-investment analysis of a worksite vitality intervention among older hospital workers: results of a randomized controlled trial. In: *Journal of occupational and environmental medicine* 55 (3), S. 337–346. DOI: 10.1097/JOM.0b013e31827b738e.

Vera, Francisca M.; Manzaneque, Juan M.; Rodríguez, Francisco M.; Vadillo, Miguel; Navajas, Federico; Heiniger, Ana I. et al. (2019): Assessment of hormonal parameters and psychological well-being in healthy subjects after a Taoist qigong program: An exploratory study. In: *Scandinavian journal of psychology* 60 (1), S. 43–49. DOI: 10.1111/sjop.12501.

Verweij, Hanne; van Ravesteijn, Hiske; van Hooff, Madelon L. M.; Lagro-Janssen, Antoine L. M.; Speckens, Anne E. M. (2018): Mindfulness-Based Stress Reduction for Residents: A Randomized Controlled Trial. In: *Journal of general internal medicine* 33 (4), S. 429–436. DOI: 10.1007/s11606-017-4249-x.

Verweij, Hanne; Waumans, Ruth C.; Smeijers, Danique; Lucassen, Peter L. B. J.; Donders, A. Rogier T.; van der Horst, Henriëtte E.; Speckens, Anne E. M. (2016): Mindfulness-based stress reduction for GPs: results of a controlled mixed methods pilot study in Dutch primary care. In: *The British journal of general practice : the journal of the Royal College of General Practitioners* 66 (643), e99-105. DOI: 10.3399/bjgp16X683497.

Visweswaraiah, N. K. (2013): Yoga for occupational health and rehabilitation.

Wang, Z. Y.; Jin, Z. (2018): 2018 IERI International Conference on Medical The Effects of mindfulness-based cognitive therapy (MBCT) on anxiety and depression among professional women: increased EEG gamma and alpha brainwave amplitude. In: *Basic & clinical pharmacology & toxicology* 123 Suppl 3, S. 3–111. DOI: 10.1111/bcpt.13100.

West, Colin P.; Dyrbye, Liselotte N.; Rabatin, Jeff T.; Call, Tim G.; Davidson, John H.; Multari, Adamarie et al. (2014): Intervention to promote physician well-being, job satisfaction, and professionalism: a randomized clinical trial. In: *JAMA internal medicine* 174 (4), S. 527–533. DOI: 10.1001/jamainternmed.2013.14387.

West, Rebecca Rae: Mindfulness skills training: An innovative approach to stress management for low SES workers.

Wetzel, Cordula M.; George, Akram; Hanna, George B.; Athanasiou, Thanos; Black, Stephen A.; Kneebone, Roger L. et al. (2011): Stress management training for surgeons-a randomized, controlled, intervention study. In: *Annals of surgery* 253 (3), S. 488–494. DOI: 10.1097/SLA.0b013e318209a594.

Yang, Tao: Why and When Does a Mindfulness Intervention Promote Job Performance? The Interpersonal Mechanisms and Individual, Job, and Social Contingencies. Online verfügbar unter <https://conservancy.umn.edu/handle/11299/178933>.

Zahedi Rad, Z.; ShafiAbadi, A.; ZareBahramAbadi, M.; Fattah Moghaddam, L. (2015): The Use of Mindfulness-based Cognitive Therapy for Improving Flow Experience of Nurses in the Psychiatric Hospitals. In: *European Psychiatry* 30, S. 1037. DOI: 10.1016/S0924-9338(15)30815-4.
