# Supplementary material for: Mindfulness-Based and Mindfulness-Informed Interventions at the Workplace: A Systematic Review and Meta-Regression Analysis of RCTs
Source: Mindfulness (N Y). 2023 May 11:1–34. Online ahead of print. doi: 10.1007/s12671-023-02130-7 (PMC10172073; doi:10.1007/s12671-023-02130-7)
Supplement: Supplementary file 5 — Supplementary file5 (PDF 286 KB) [file 12671_2023_2130_MOESM5_ESM.pdf]

Authors	Randomization process	Deviations from intended interventions	Missing outcome data	Measurement of the outcome	Selection of the reported result	Overall
Aikens et al. 2014	?	−	+	−	?	−
Alexander et al. 2015	?	−	−	−	?	−
Allexandre et al. 2016	?	−	+	−	?	−
Amutio et al. 2015a; Amutio et al. 2015b	?	−	−	−	−	−
Arredondo et al. 2017	?	−	?	−	?	−
Baby et al. 2019	+	?	−	+	+	−
Baccarani et al. 2013	?	−	+	−	?	−
Bartlett et al. 2017	−	−	−	−	?	−
Bhandari et al. 2010	?	−	+	−	?	−
Bhandari 2017	?	−	+	−	?	−
Bostock et al. 2019	?	?	+	−	?	−
Brinkborg et al. 2011	?	−	+	−	?	−
Calder Calisi 2017	?	−	−	−	?	−
Cheema et al. 2013	+	+	+	+	+	+
Chin et al. 2019; Slutsky et al. 2019	?	?	−	−	−	−
Christopher et al. 2018	?	−	−	−	?	−
Coelhoso et al. 2019	?	?	−	−	−	−
Cook et al. 2017a	?	−	−	−	?	−
Crain et al. 2017	?	−	−	−	?	−
Dahl 2019; Dahl and Dlugosch 2020	?	−	−	−	−	−
Duchemin et al. 2015; Steinberg et al. 2016	?	−	+	−	−	−
Dwivedi et al. 2015; Dwivedi et al. 2016a	+	+	+	+	−	−
Elder et al. 2014	+	−	−	−	?	−
Fang and Li 2015	?	−	−	−	?	−

Flaxman and Bond 2010	?	−	−	−	?	−
Flook et al. 2013	?	−	+	−	?	−
Franco et al. 2010	?	−	+	+	?	−
Grégoire and Lachance 2015	−	−	−	−	?	−
Grégoire et al. 2015	−	−	−	−	?	−
Hartfield et al. 2011	−	−	−	−	?	−
Hartfiel et al. 2012	?	−	−	−	?	−
Hartfiel et al. 2017	?	−	−	−	−	−
Huang et al. 2015	?	−	−	−	?	−
Hülsheger et al. 2013	?	?	−	−	?	−
Hülsheger et al. 2015	−	−	−	−	?	−
Ireland et al. 2017	?	−	+	−	?	−
Jennings et al. 2017	?	−	?	−	?	−
Klatt et al. 2009	−	−	−	−	?	−
Klatt et al. 2017	?	−	−	−	?	−
Krick and Felfe 2020	?	−	+	−	?	−
Lacerda et al. 2018	?	−	−	−	?	−
Lebares et al. 2019	?	−	?	+	+	−
Lemaire et al. 2011b	?	−	+	−	?	−
Lilly et al. 2019	?	−	−	−	?	−
Lin et al. 2015	−	−	+	−	?	−
Lin et al. 2019	?	−	−	−	?	−
Ludwigs et al. 2019	+	−	−	−	?	−
Mackenzie et al. 2006	−	−	−	−	?	−
Maddux et al. 2018	?	+	−	−	?	−
Manotas et al. 2014	?	−	−	−	?	−
Masih et al. 2020	+	−	+	−	−	−
McConachie et al. 2014	+	−	?	−	?	−
Michel et al. 2014; Rexroth et al. 2017	?	−	−	−	−	−
Mino et al. 2006	?	−	−	−	?	−
Mistretta et al. 2018	?	−	+	−	?	−
Molek-Winiarska and Zolnierczyk-Zreda 2018	?	−	+	−	?	−
Möltner et al. 2018	?	−	−	−	?	−

Nübold et al. 2019	?	–	+	–	?	–
O'Brien et al. 2019	?	+	–	–	?	–
Pandya 2019	+	–	–	–	?	–
Pang and Ruch 2019	?	–	+	–	?	–
Pipe et al. 2009	?	–	–	–	?	–
Querstret et al. 2017	+	–	–	–	?	–
Rao et al. 2017	?	+	+	–	?	–
Riley et al. 2017	?	+	–	–	?	–
Roeser et al. 2013	?	–	–	–	–	–
Sakuma et al. 2012	+	–	–	–	?	–
Schroeder et al. 2018	?	+	–	–	?	–
Shonin et al. 2014	?	–	–	–	?	–
Singh et al. 2016	?	–	+	–	?	–
Singh et al. 2020	?	–	–	–	?	–
Smith et al. 2020	?	–	–	–	?	–
Sood et al. 2011	?	–	–	–	?	–
Sood et al. 2014	+	–	–	–	?	–
Sutarto et al. 2012	?	–	–	–	?	–
Tahamsebi et al. 2018	?	+	–	–	?	–
Taylor et al. 2016	–	–	+	–	–	–
Telles et al. 2012	–	?	+	–	?	–
Valley and Stallones 2017	?	–	–	–	?	–
van Berkel et al. 2014; van Dongen et al. 2016	+	–	–	–	–	–
Versluis et al. 2018	?	–	–	–	?	–
Watanabe et al. 2019	?	–	+	–	+	–
Wolever et al. 2012	?	–	–	–	?	–
Yang et al. 2018	?	–	+	–	?	–
Zolnierczyk-Zreda et al. 2016	+	–	–	–	?	–

+ Low risk

? Some concerns

– High risk
