# Supplementary material for: Mindfulness-Based and Mindfulness-Informed Interventions at the Workplace: A Systematic Review and Meta-Regression Analysis of RCTs
Source: Mindfulness (N Y). 2023 May 11:1–34. Online ahead of print. doi: 10.1007/s12671-023-02130-7 (PMC10172073; doi:10.1007/s12671-023-02130-7)
Supplement: Supplementary file 6 — Supplementary file6 (DOCX 6096 KB) [file 12671_2023_2130_MOESM6_ESM.docx]

**Supplementary Online Material 6: Results and sensitivity analyses of broad categories**

TE = Treatment effect (standardized mean difference – SMD), seTE = standard error of treatment effect

# Post-intervention

## 1.1 Mindfulness

### Figure S6.1.1.1: Forest plot – Mindfulness with all studies
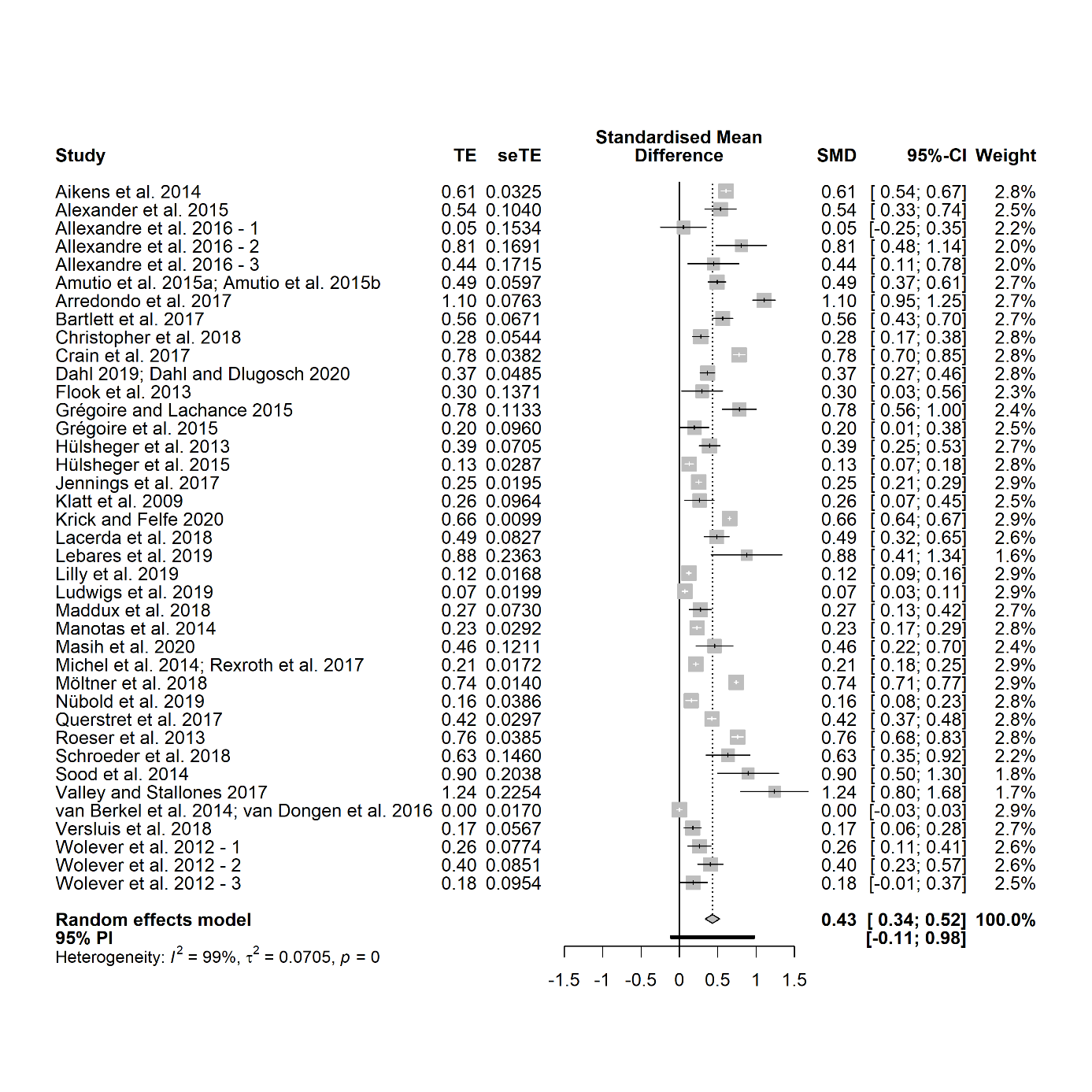


### Figure S6.1.1.2: Funnel plot – Mindfulness with all studies


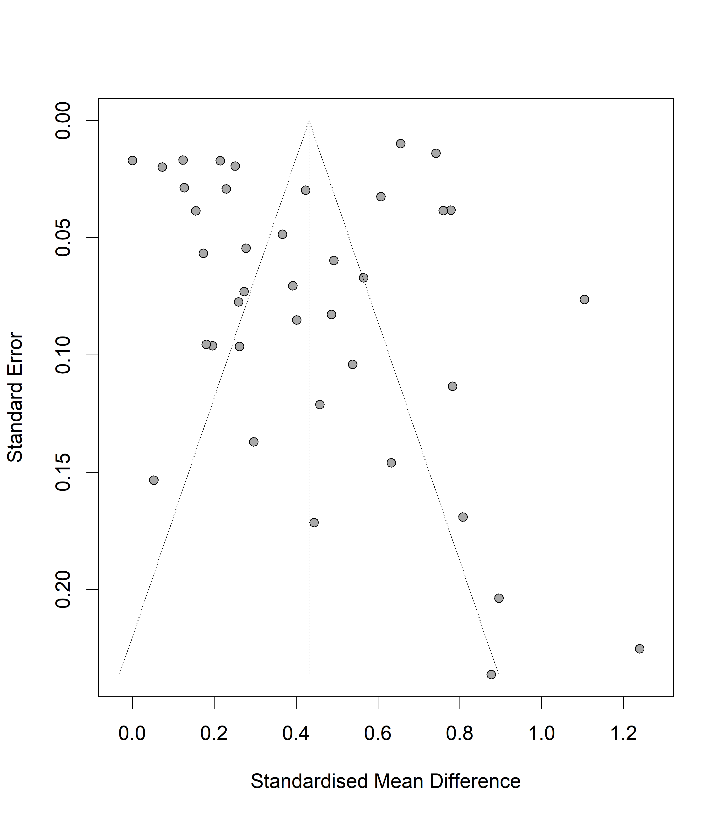


### Table S6.1.1.1: Egger’s test – Mindfulness with all studies

*Eggers' test of the intercept*

| intercept | 95% CI | t | p |
| --- | --- | --- | --- |
| -0.703 | [-4.97 - 3.56] | -0.323 | 0.748 |

Eggers' test does not indicate the presence of funnel plot asymmetry.

*P-curve analysis*

- Total number of provided studies: k = 39

- Total number of p<0.05 studies included into the analysis: k = 36 (92.31%)

- Total number of studies with p<0.025: k = 34 (87.18%)

### Figure S6.1.1.3: Forest plot – Mindfulness without outliers


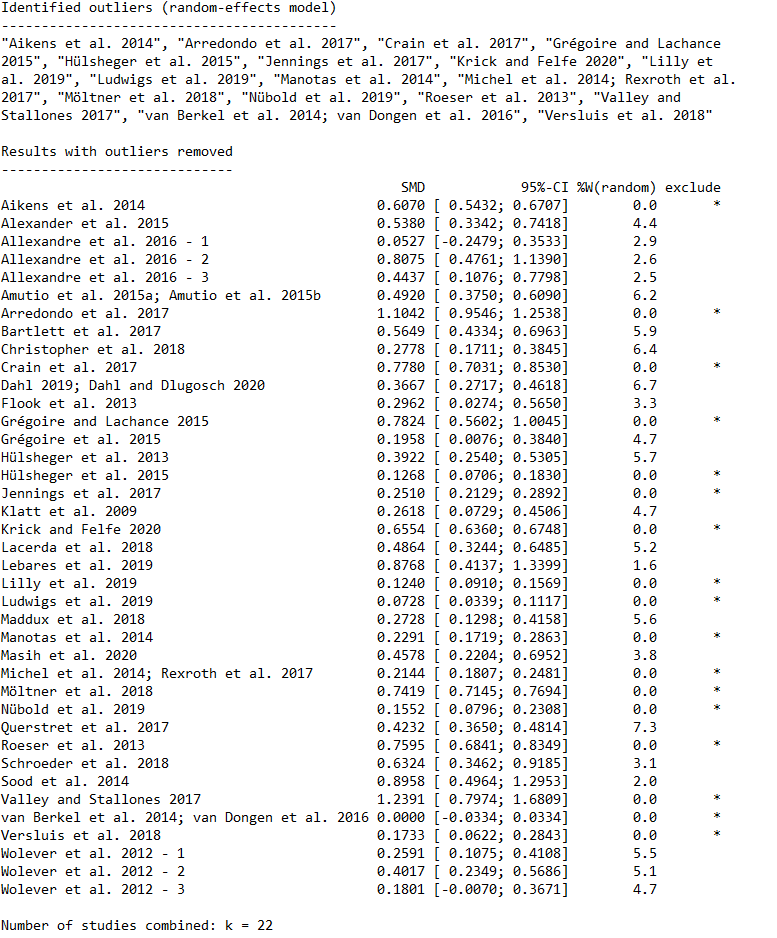


## 1.2 Well-being

### Figure S6.1.2.1: Forest plot – Well-being with all studies


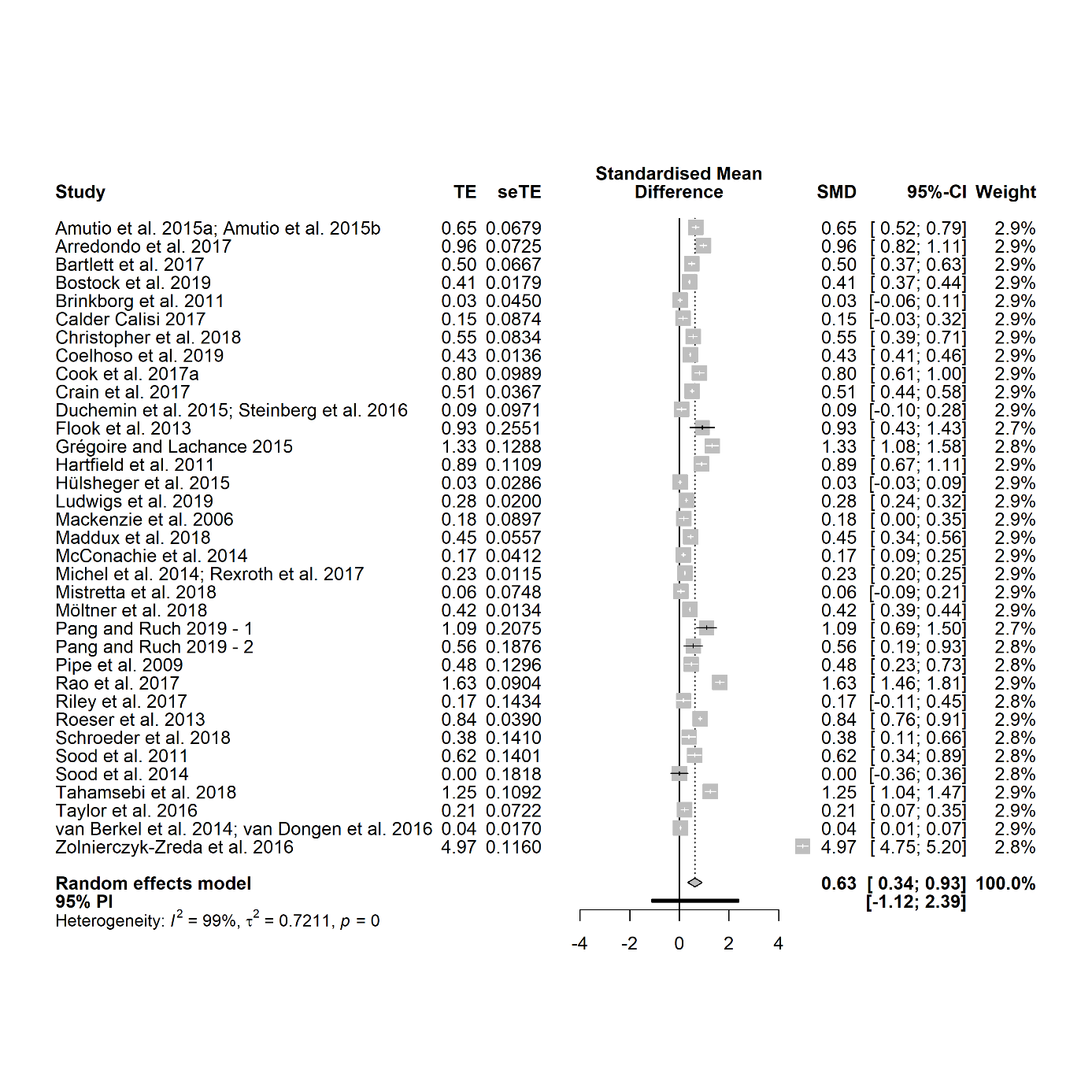


### Figure S6.1.2.2: Funnel plot – Well-being with all studies


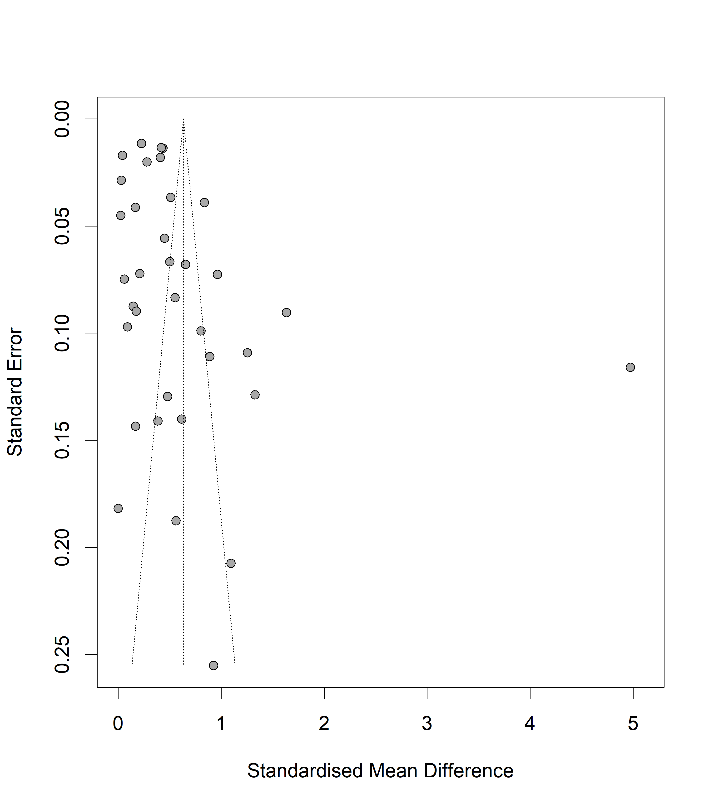


### Table S6.1.2.1: Egger's test – Well-being with all studies

*Eggers' test of the intercept*

| intercept | 95% CI | t | p |
| --- | --- | --- | --- |
| 4.602 | 0.39;8.81] | 2.144 | 0.0395 |

Eggers' test indicates the presence of funnel plot asymmetry.

*P-curve analysis*

- Total number of provided studies: k = 35

- Total number of p<0.05 studies included into the analysis: k = 28 (80%)

- Total number of studies with p<0.025: k = 27 (77.14%)

### Figure S6.1.2.3: Forest plot – Well-being without outliers


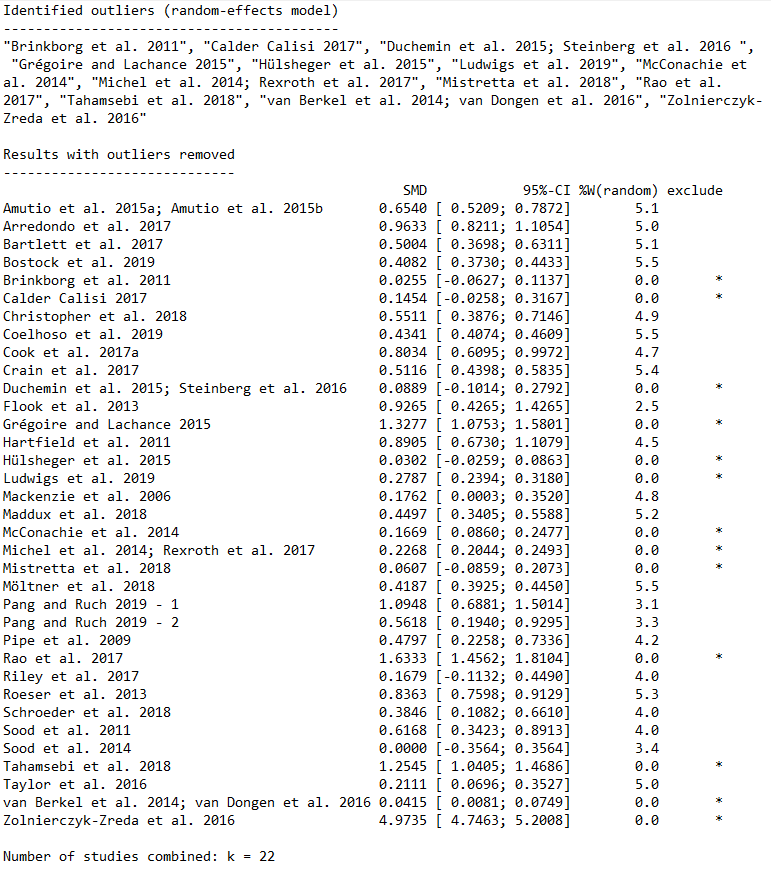


## Physical Health

### Figure S6.1.3.1: Forest plot – Physical Health with all studies


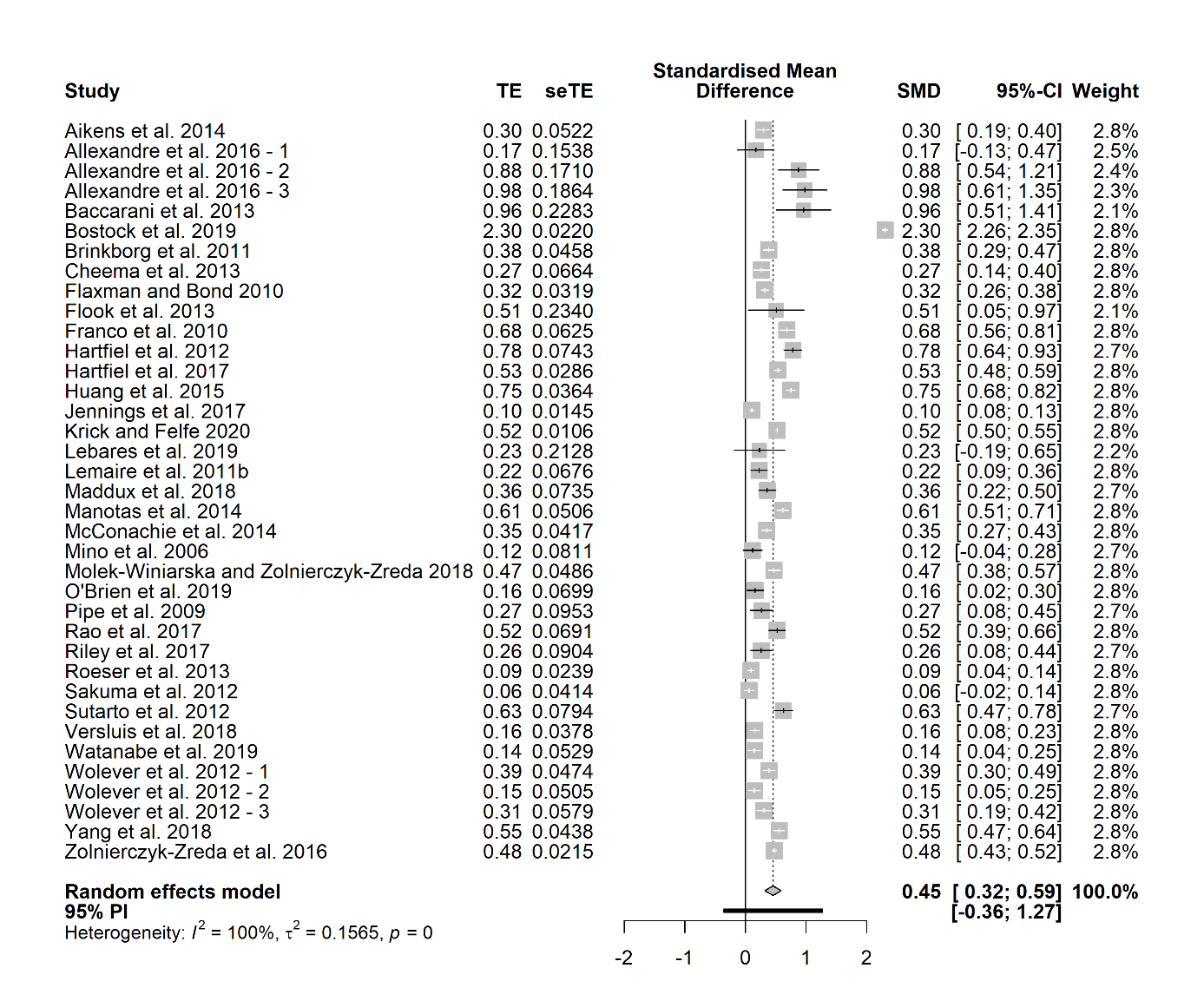


### Figure S6.1.3.2: Funnel plot – Physical Health with all studies


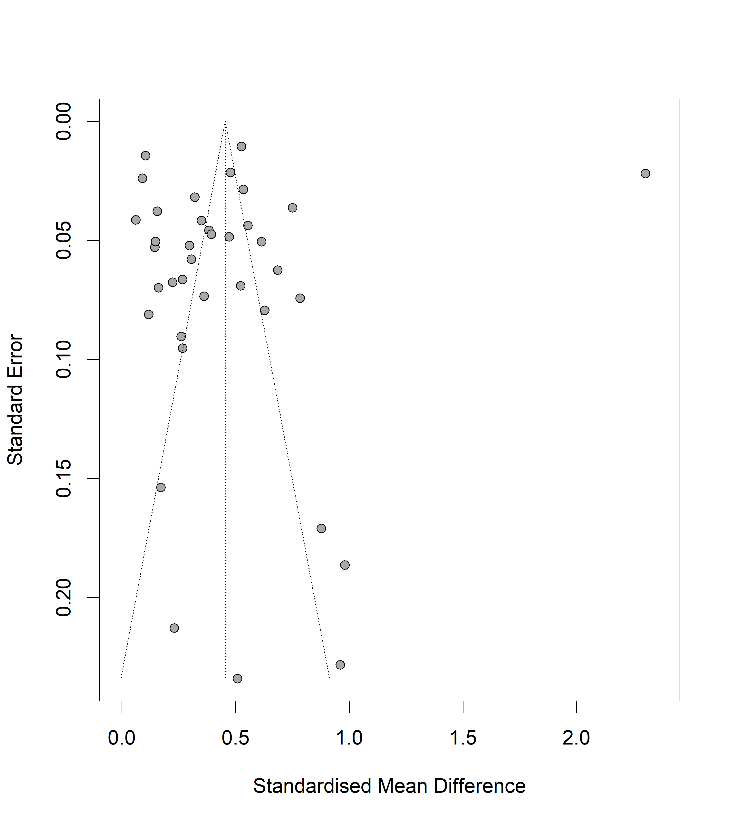


### Table S6.1.3.1: Egger's test – Physical Health with all studies

*Eggers' test of the intercept*

| intercept | 95% CI | t | p |
| --- | --- | --- | --- |
| -1.448 | [-9.36; 6.47] | -0.359 | 0.722 |

Eggers' test does not indicate the presence of funnel plot asymmetry.

*P-curve analysis*

- Total number of provided studies: k = 37

- Total number of p<0.05 studies included into the analysis: k = 33 (89.19%)

- Total number of studies with p<0.025: k = 32 (86.49%) Mental Health

### Figure S6.1.3.3: Forest plot – Physical Health without outliers


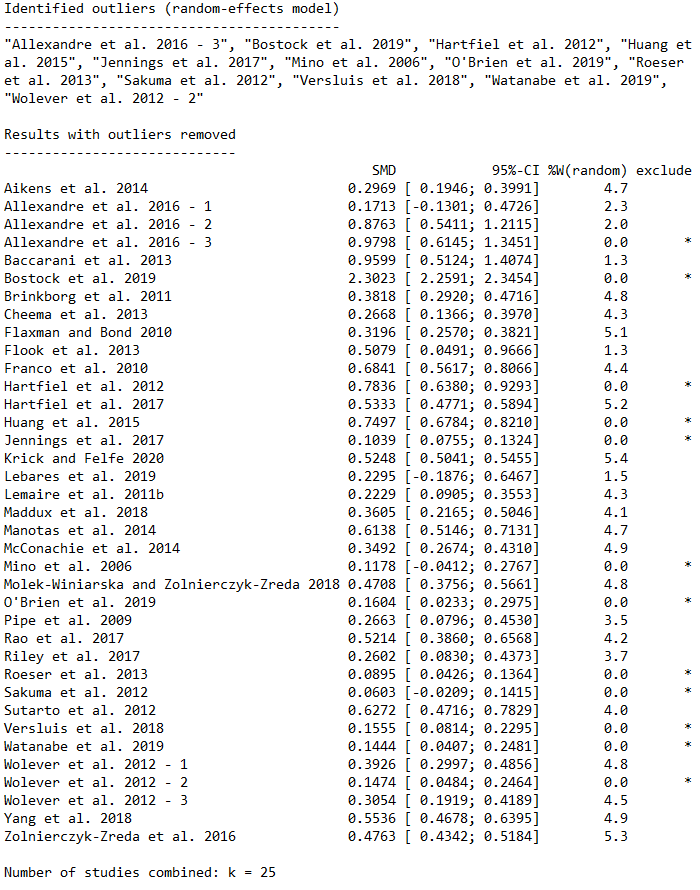


## Mental Health

### Figure S6.1.4.1: Forest plot – Mental Health with all studies


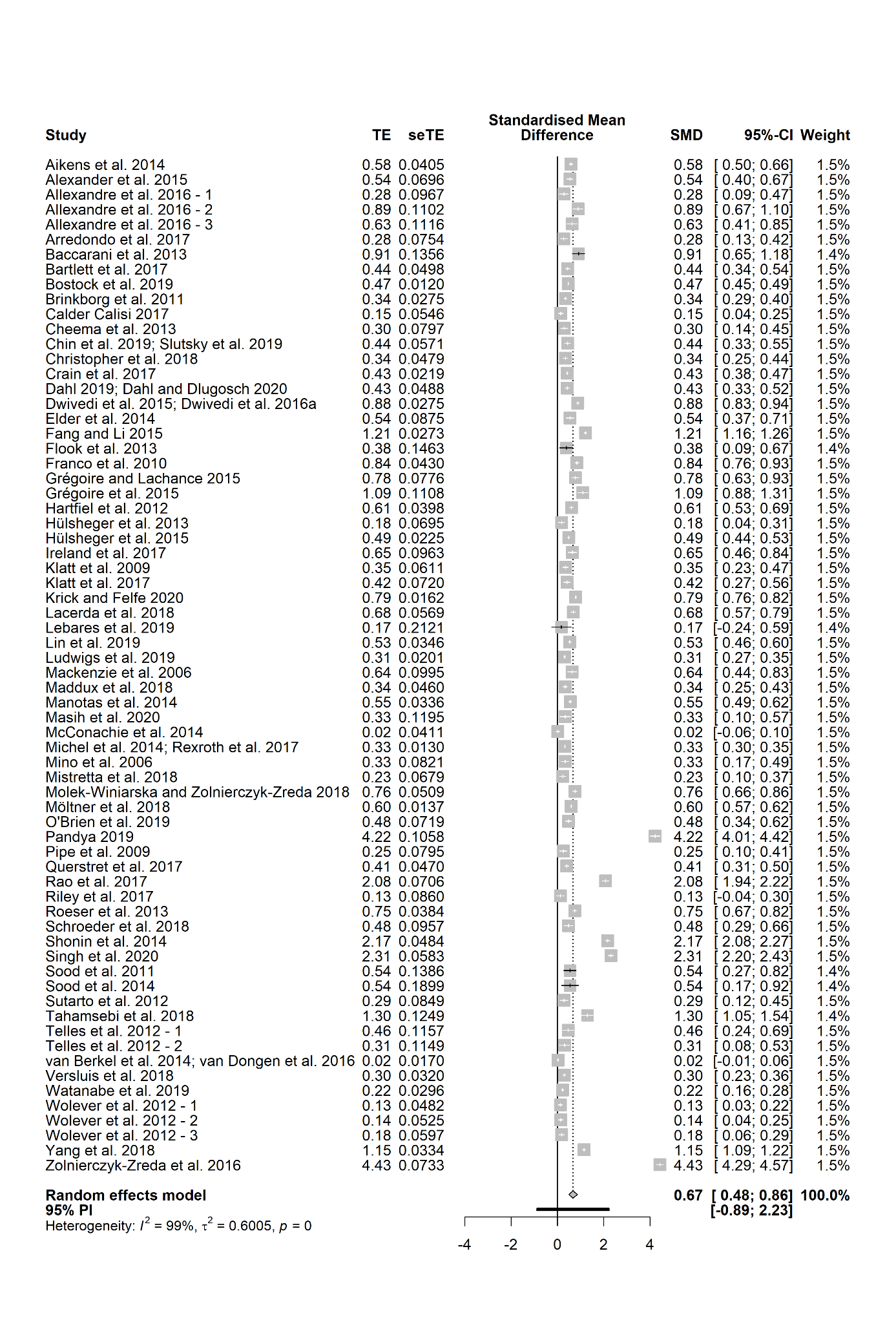


### Figure S6.1.4.2: Funnel plot – Mental Health with all studies


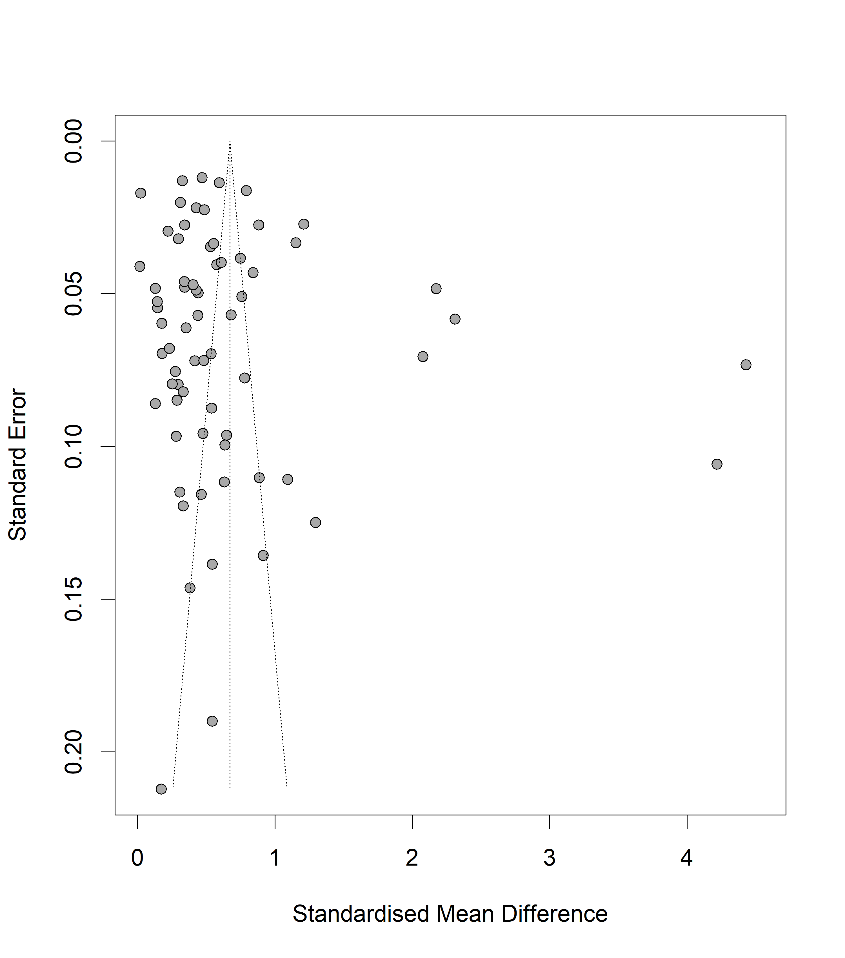


### Table S6.1.4.1: Egger's test – Mental Health with all studies

*Eggers' test of the intercept*

| intercept | 95% CI | t | p |
| --- | --- | --- | --- |
| 4.32 | [-0.44; 9.09] | 1.777 | 0.080 |

Eggers' test does not indicate the presence of funnel plot asymmetry.

*P-curve analysis*

- Total number of provided studies: k = 68

- Total number of p<0.05 studies included into the analysis: k = 64 (94.12%)

- Total number of studies with p<0.025: k = 64 (94.12%)

### Figure S6.1.4.3: Forest plot – Mental Health without outliers


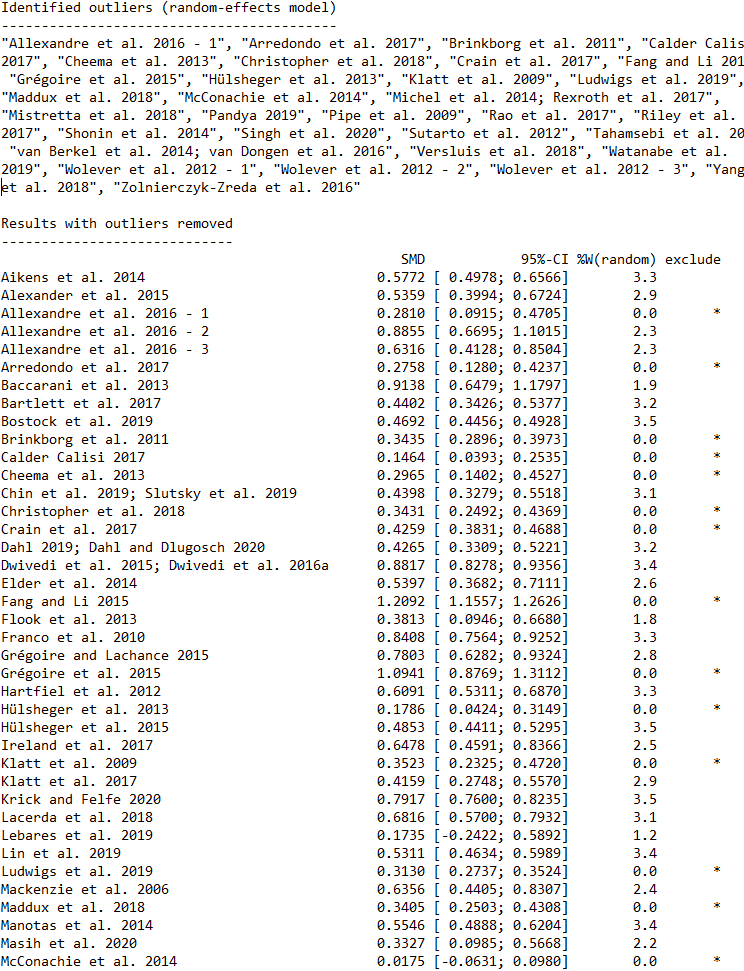


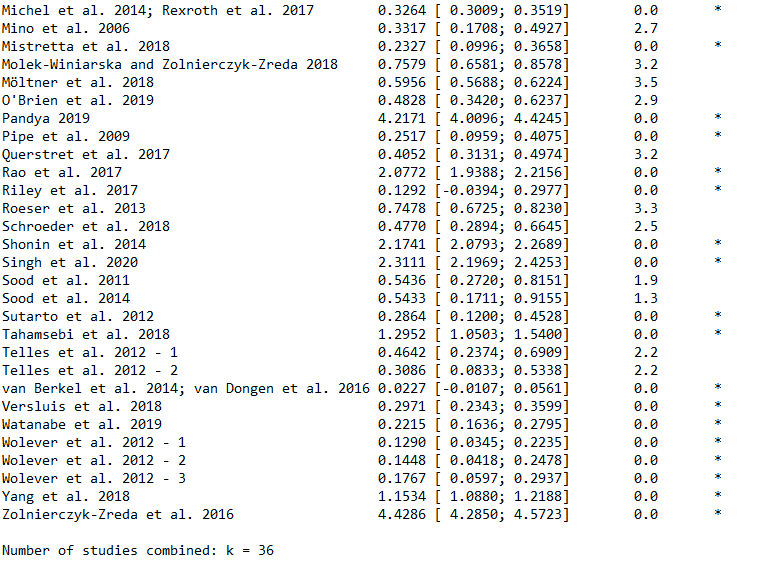


## Stress

### Figure S6.1.5.1: Forest plot – Stress with all studies


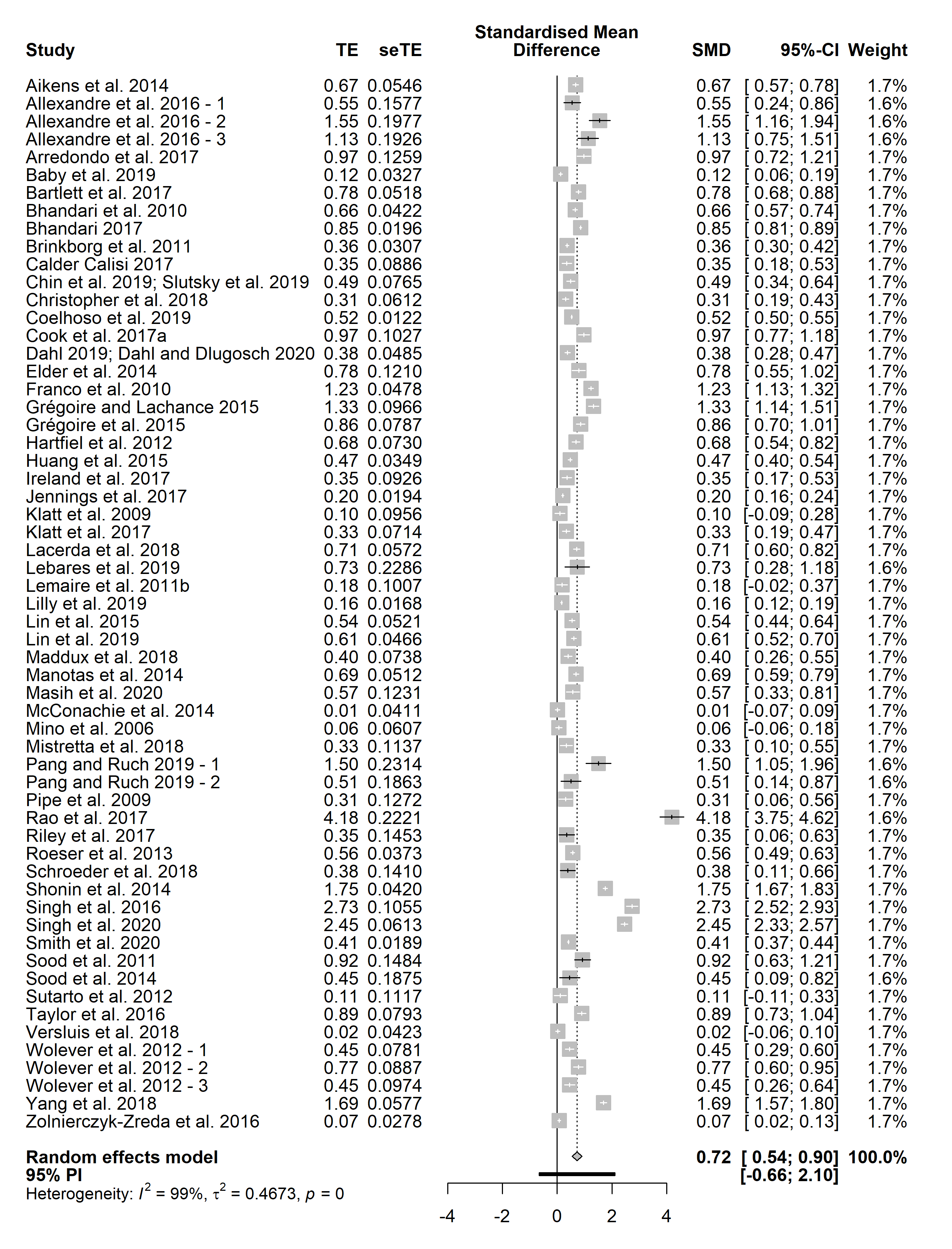


### Figure S6.1.5.2: Funnel plot – Stress with all studies


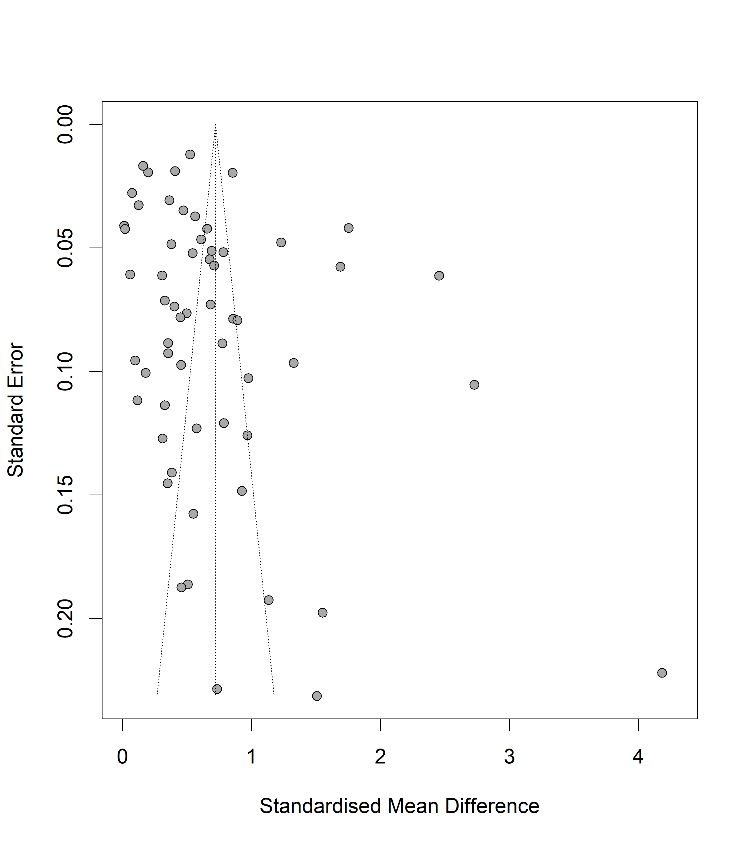


### Table S6.1.5.1: Egger's test – Stress with all studies

*Eggers' test of the intercept*

| intercept | 95% CI | t | p |
| --- | --- | --- | --- |
| 4.298 | [0.56;8.03] | 2.256 | 0.0279 |

Eggers' test does not indicate the presence of funnel plot asymmetry.

*P-curve analysis*

- Total number of provided studies: k = 59

- Total number of p<0.05 studies included into the analysis: k = 53 (89.83%)

- Total number of studies with p<0.025: k = 53 (89.83%)

### Figure S6.1.5.3: Forest plot – Stress without outliers


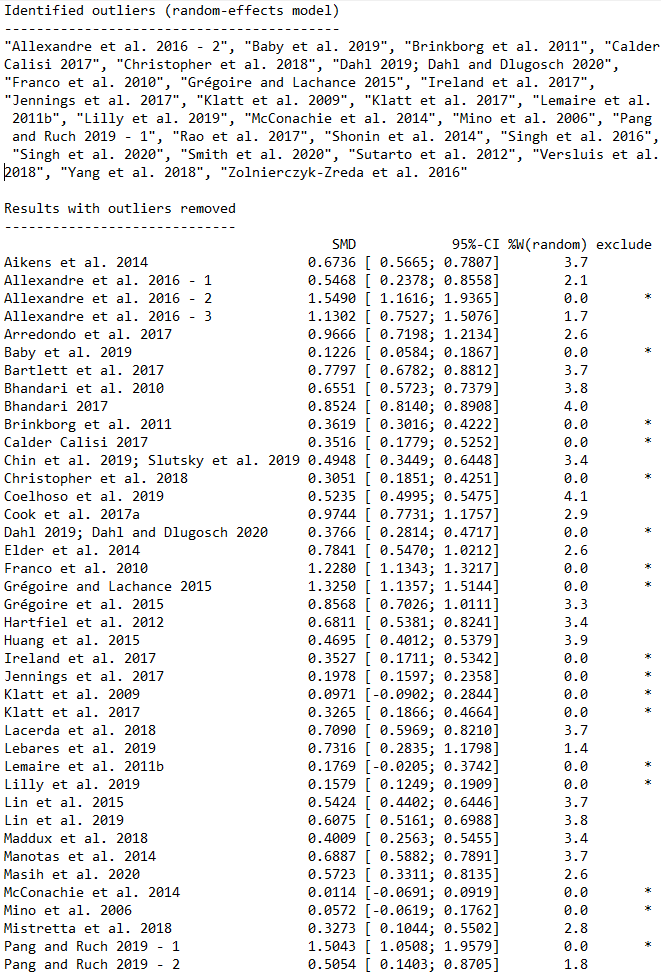


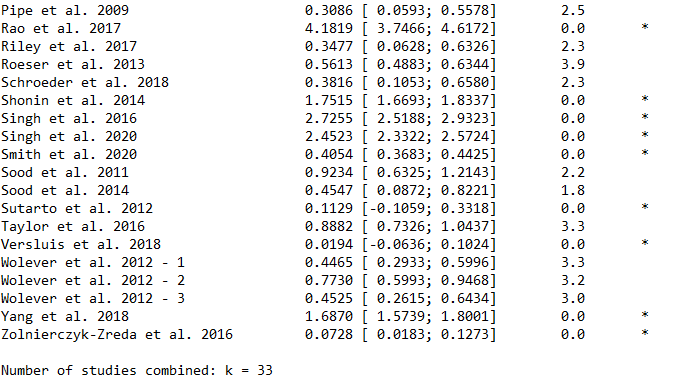


## Resilience

### Figure S6.1.6.1: Forest plot – Resilience with all studies


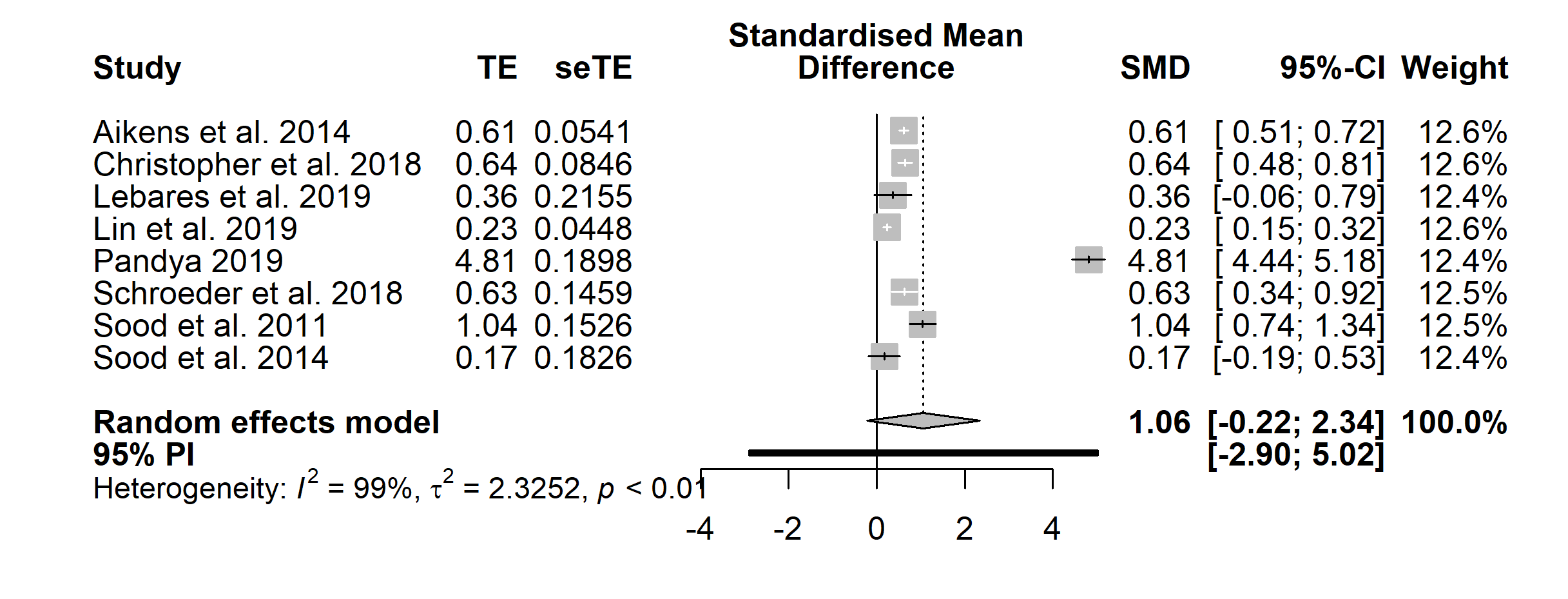


### Figure S6.1.6.2: Funnel plot – Resilience with all studies


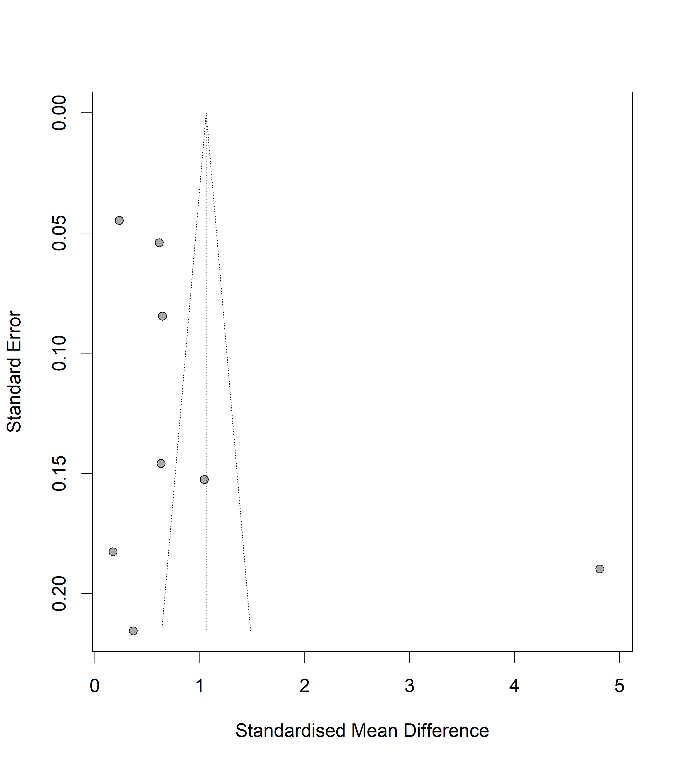


### Table S6.1.6.1: Egger's test – Resilience with all studies

*Eggers' test of the intercept*

| intercept | 95% CI | t | p |
| --- | --- | --- | --- |
| 8.153 | -2.89;19.2] | 1.447 | 0.198 |

Eggers' test does not indicate the presence of funnel plot asymmetry.

*P-curve analysis*

- Total number of provided studies: k = 8

- Total number of p<0.05 studies included into the analysis: k = 6 (75%)

- Total number of studies with p<0.025: k = 6 (75%)

### Figure S6.1.6.3: Forest plot – Resilience without outliers


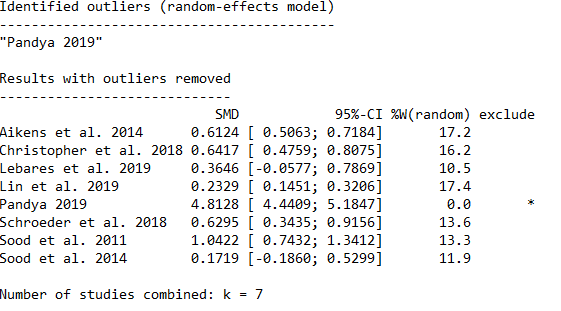


## 1.7 Work-related factors

### Figure S6.1.7.1: Forest plot – Work-related factors with all studies


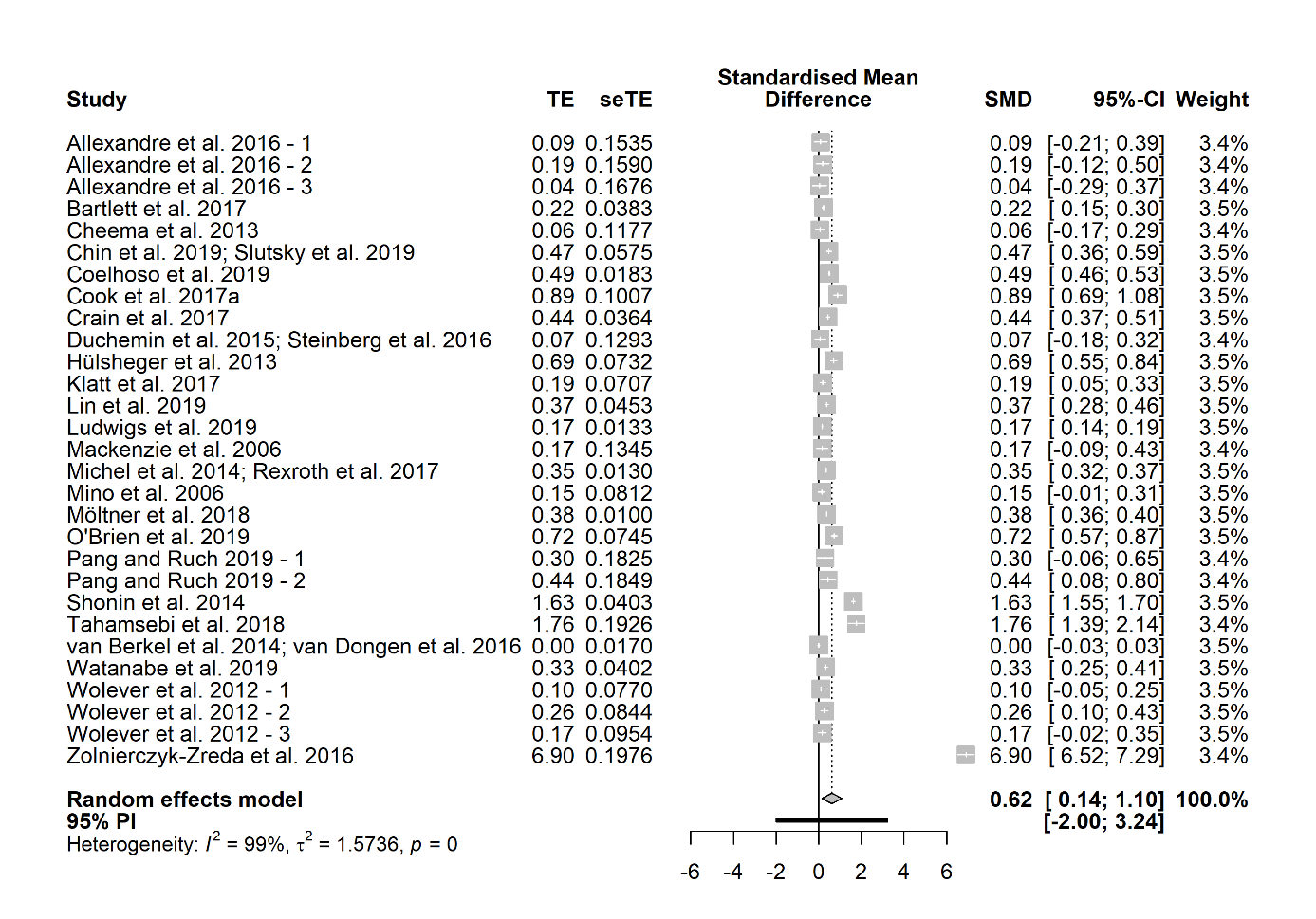


### Figure S6.1.7.2: Funnel plot – Work-related factors with all studies


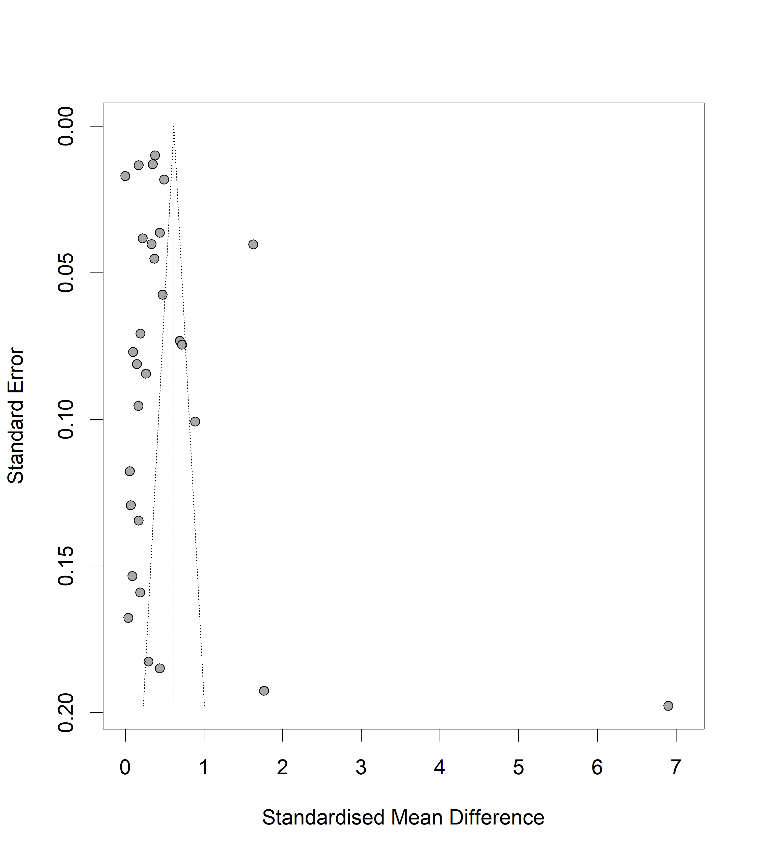


### Table S6.1.7.1: Egger's test – Work-related factors with all studies

*Eggers' test of the intercept*

| intercept | 95% CI | t | p |
| --- | --- | --- | --- |
| 3.535 | [1.53; 8.6] | 1.369 | 0.182 |

Eggers' test does not indicate the presence of funnel plot asymmetry.

*P-curve analysis*

- Total number of provided studies: k = 29

- Total number of p<0.05 studies included into the analysis: k = 18 (62.07%)

- Total number of studies with p<0.025: k = 18 (62.07%)

### Figure S6.1.7.3: Forest plot – Work-related factors without outliers


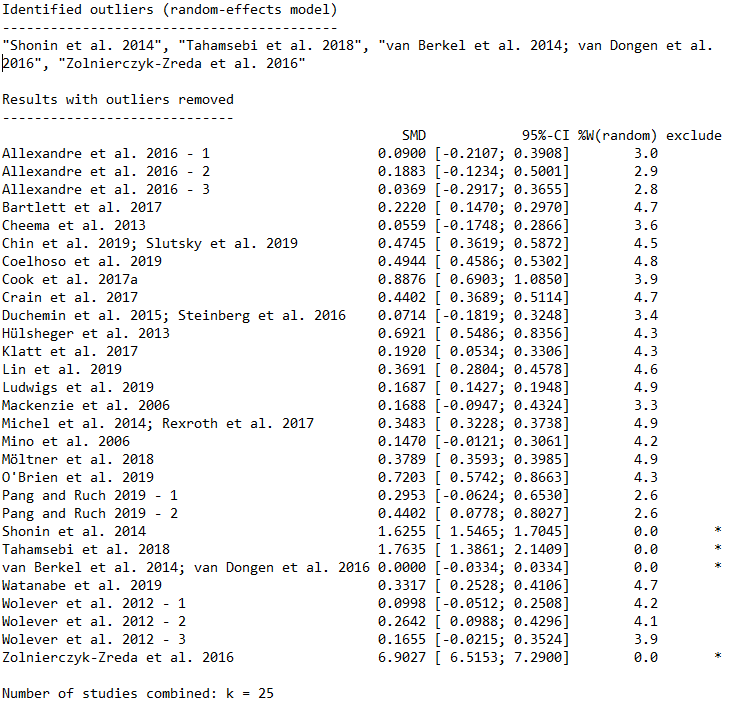


# Short-term follow-up

## Mindfulness

### Figure S6.2.1.1: Forest plot – Mindfulness with all studies


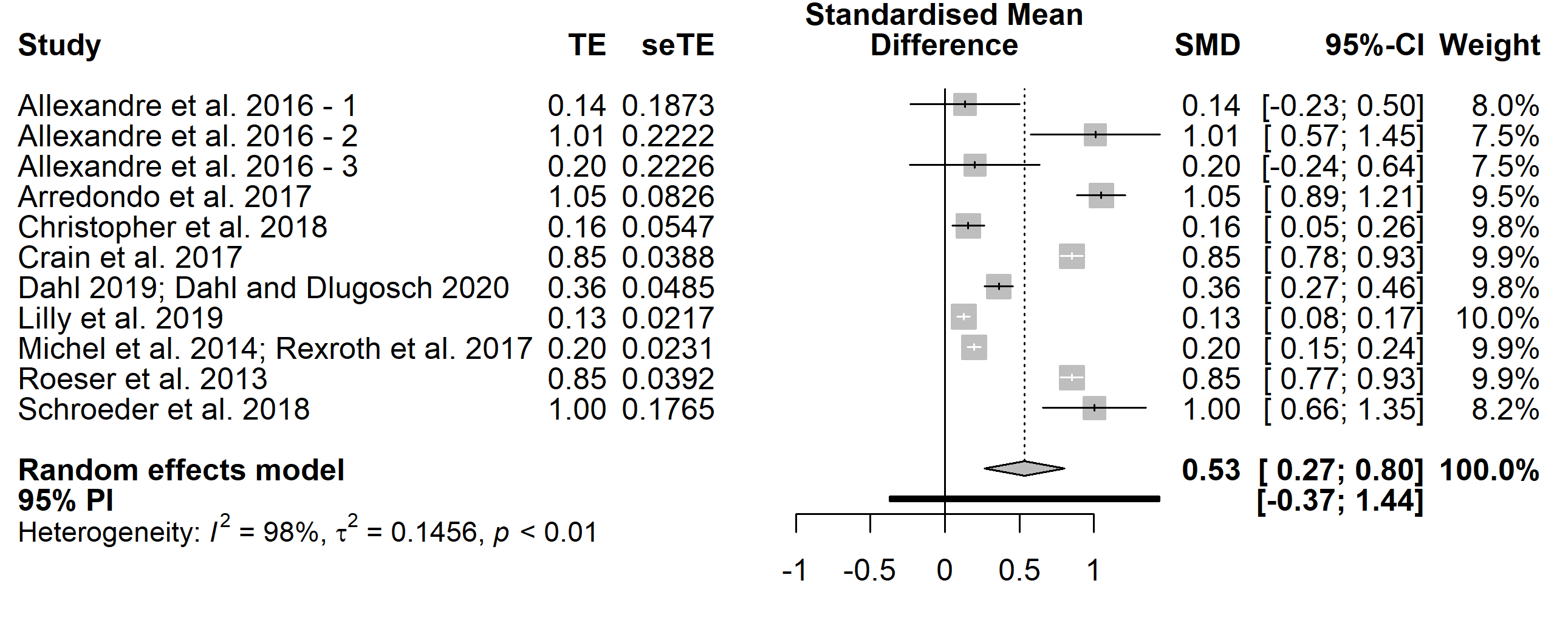


### Figure S6.2.1.2: Funnel plot – Mindfulness with all studies


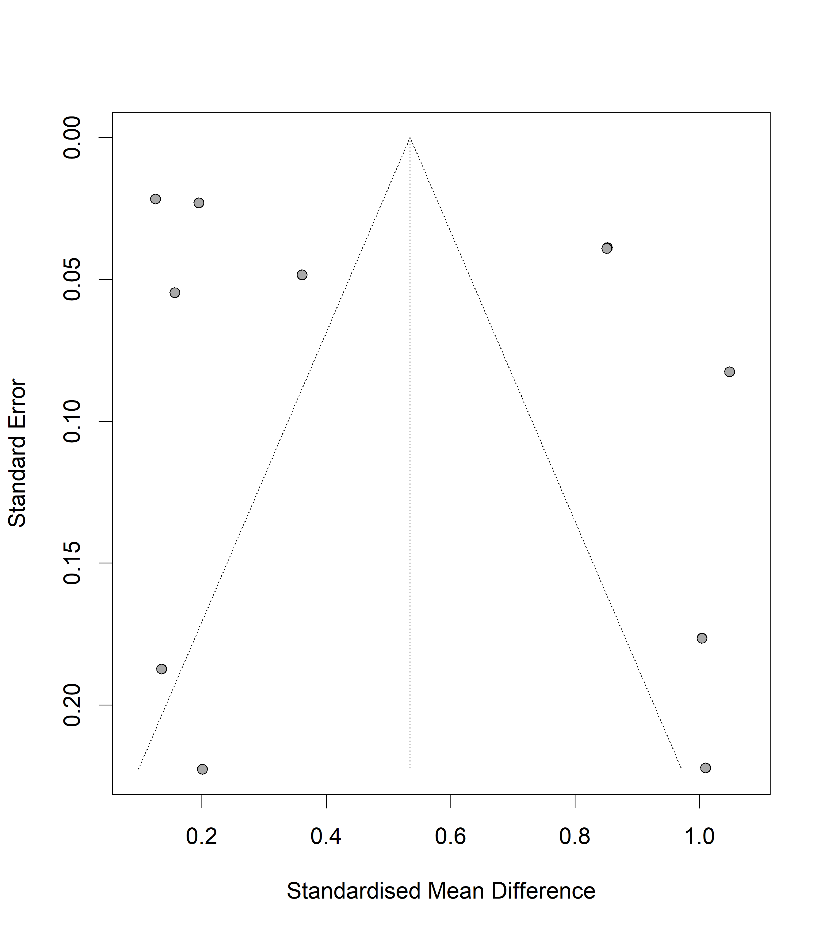


### Table S6.2.1.1: Egger’s test – Mindfulness with all studies

*Eggers' test of the intercept*

| intercept | 95% CI | t | p |
| --- | --- | --- | --- |
| 5.116 | [-2.18; 12.42] | 1.374 | 0.203 |

Eggers' test does not indicate the presence of funnel plot asymmetry.

*P-curve analysis*

- Total number of provided studies: k = 11

- Total number of p<0.05 studies included into the analysis: k = 9 (81.82%)

- Total number of studies with p<0.025: k = 9 (81.82%)

### Figure S6.2.1.3: Forest plot – Mindfulness without outliers


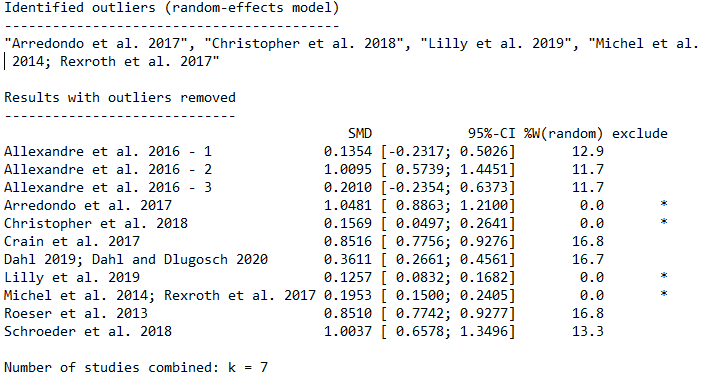


## Well-being

### Figure S6.2.2.1: Forest plot – Well-being with all studies


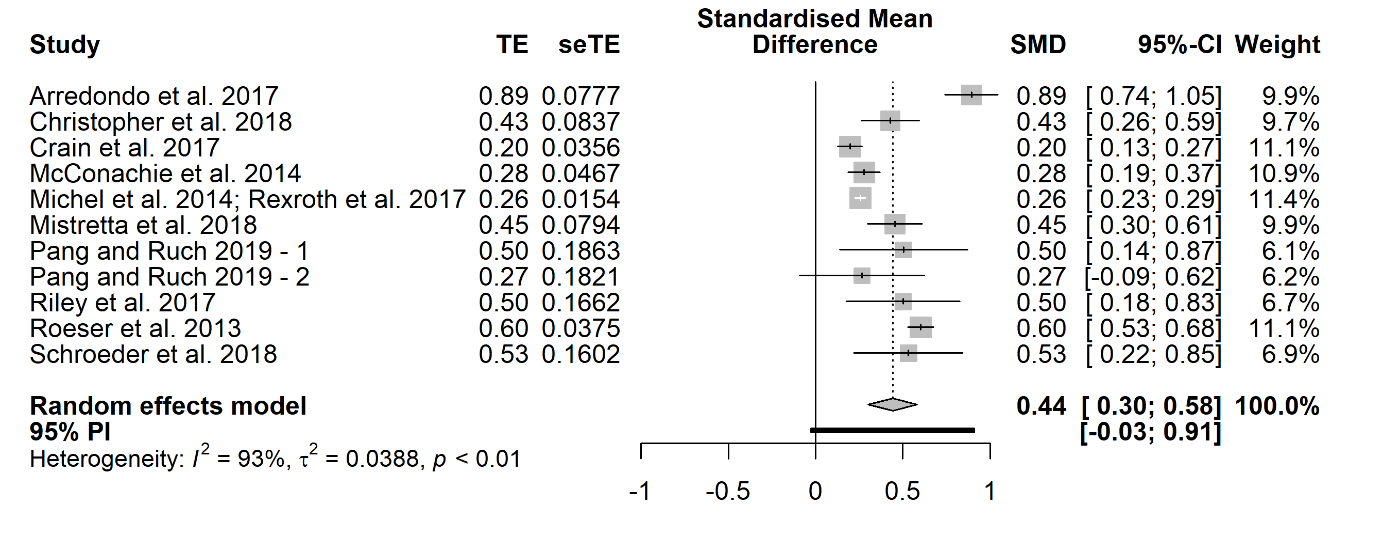


### Figure S6.2.2.2: Funnel plot – Well-being with all studies


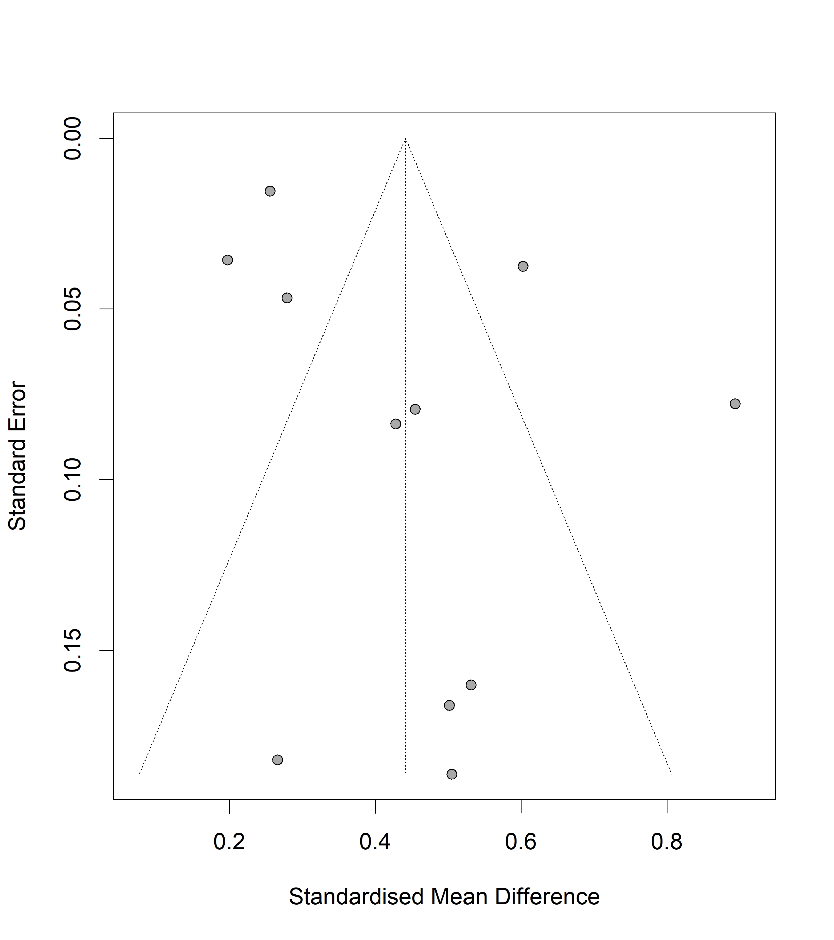


### Table S6.2.2.1: Egger’s test – Well-being with all studies

*Eggers' test of the intercept*

| intercept | 95% CI | t | p |
| --- | --- | --- | --- |
| 2.714 | [-0.4; 5.82] | 1.711 | 0.121 |

Eggers' test does not indicate the presence of funnel plot asymmetry.

*P-curve analysis*

- Total number of provided studies: k = 11

- Total number of p<0.05 studies included into the analysis: k = 10 (90.91%)

- Total number of studies with p<0.025: k = 10 (90.91%)

### Figure S6.2.2.3: Forest plot – Well-being without outliers


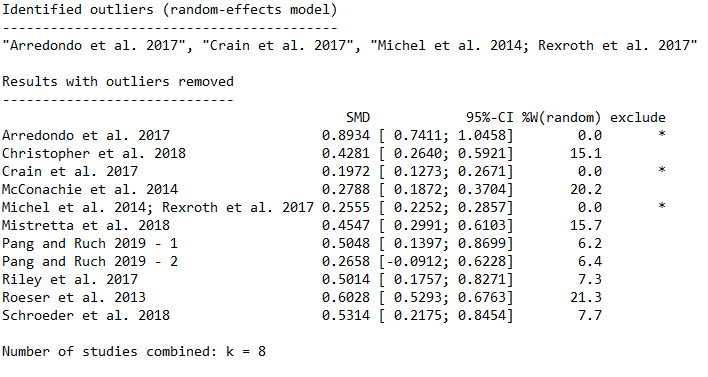


## Physical Health

### Figure S6.2.3.1: Forest plot – Physical Health with all studies


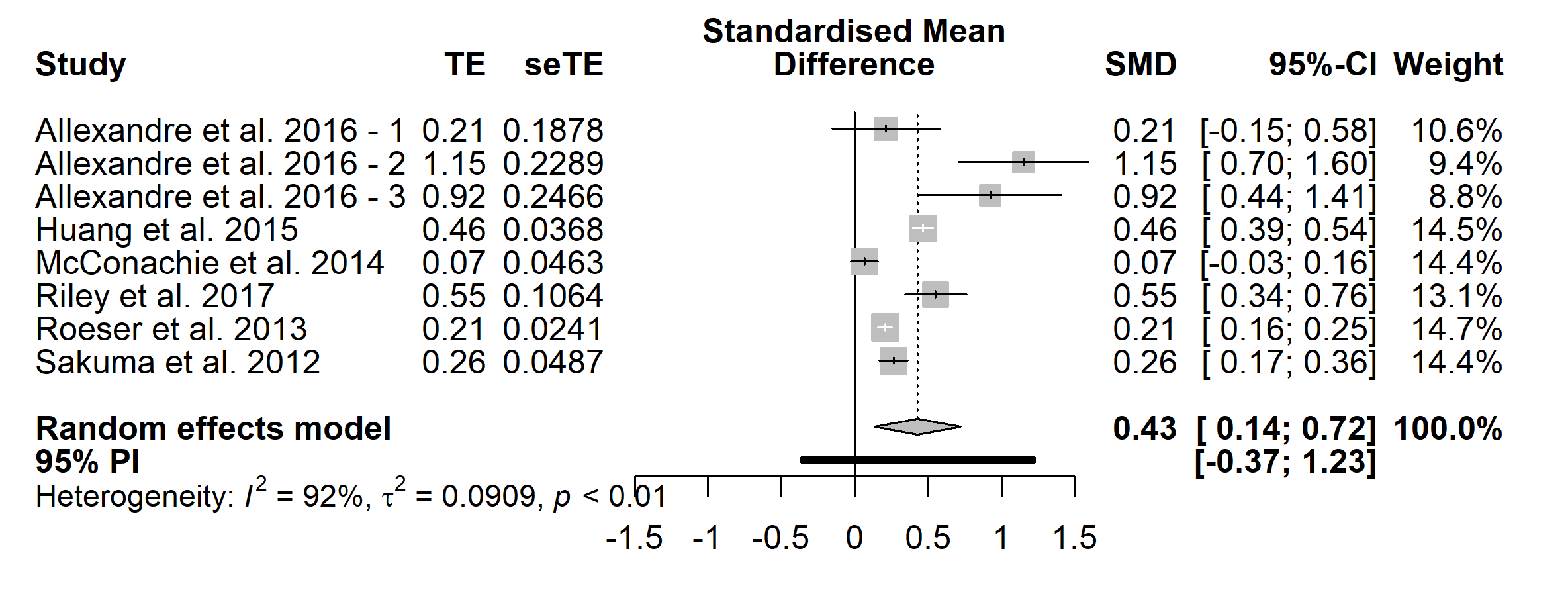


### Figure S6.2.3.2: Funnel plot – Physical Health with all studies


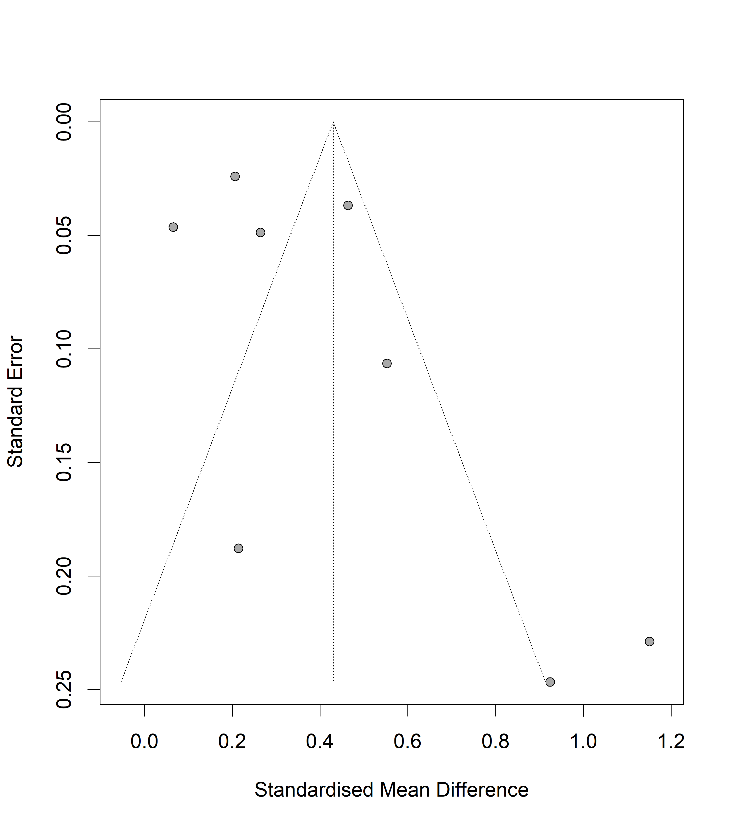


### Table S6.2.3.1: Egger’s test – Physical Health with all studies

*Eggers' test of the intercept*

| intercept | 95% CI | t | p |
| --- | --- | --- | --- |
| 2.594 | [-1.18; 6.36] | 1.349 | 0.226 |

Eggers' test does not indicate the presence of funnel plot asymmetry.

*P-curve analysis*

- Total number of provided studies: k = 8

- Total number of p<0.05 studies included into the analysis: k = 6 (75%)

- Total number of studies with p<0.025: k = 6 (75%)

## Mental Health

### Figure S6.2.4.1: Forest plot – Mental Health with all studies


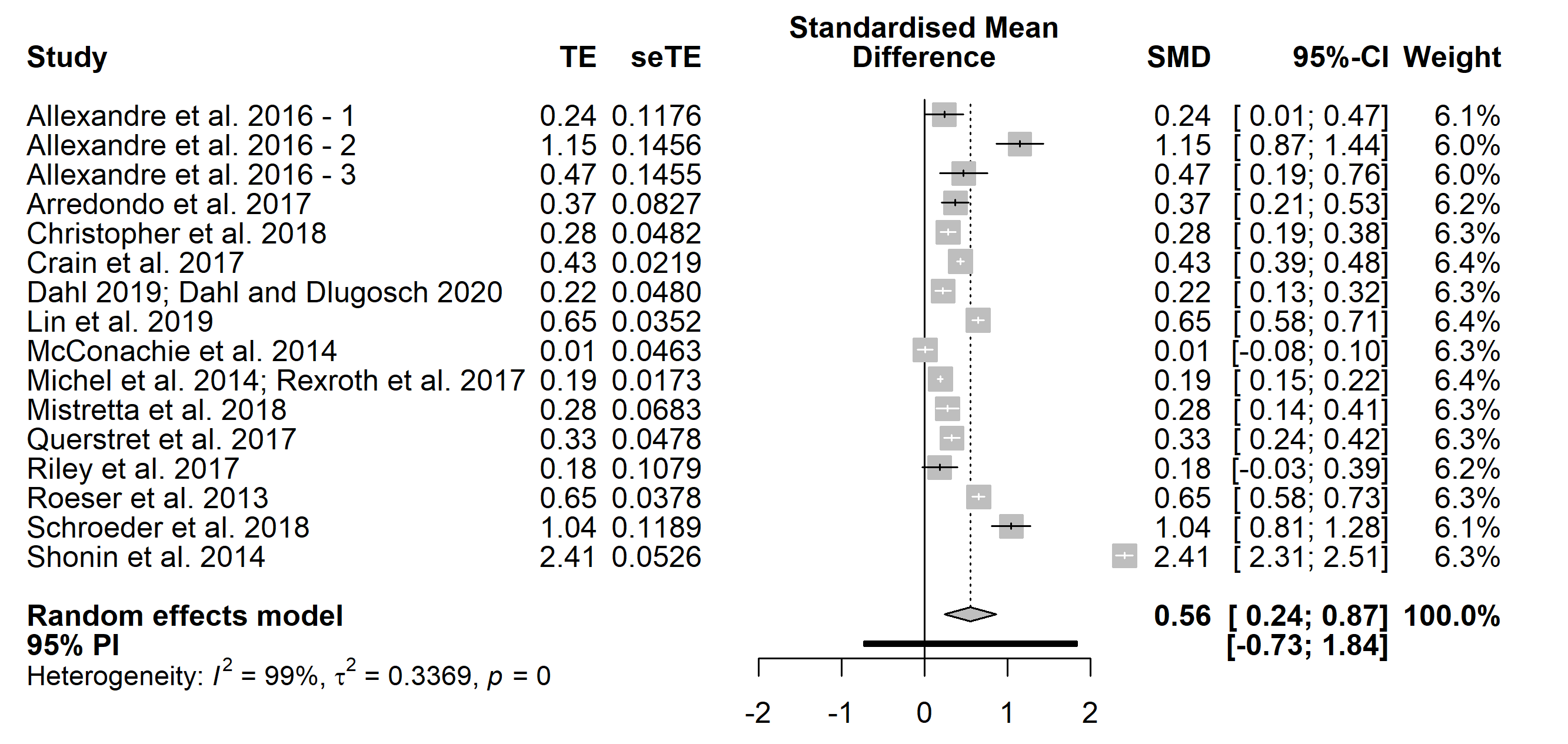


### Figure S6.2.4.2: Funnel plot – Mental Health with all studies


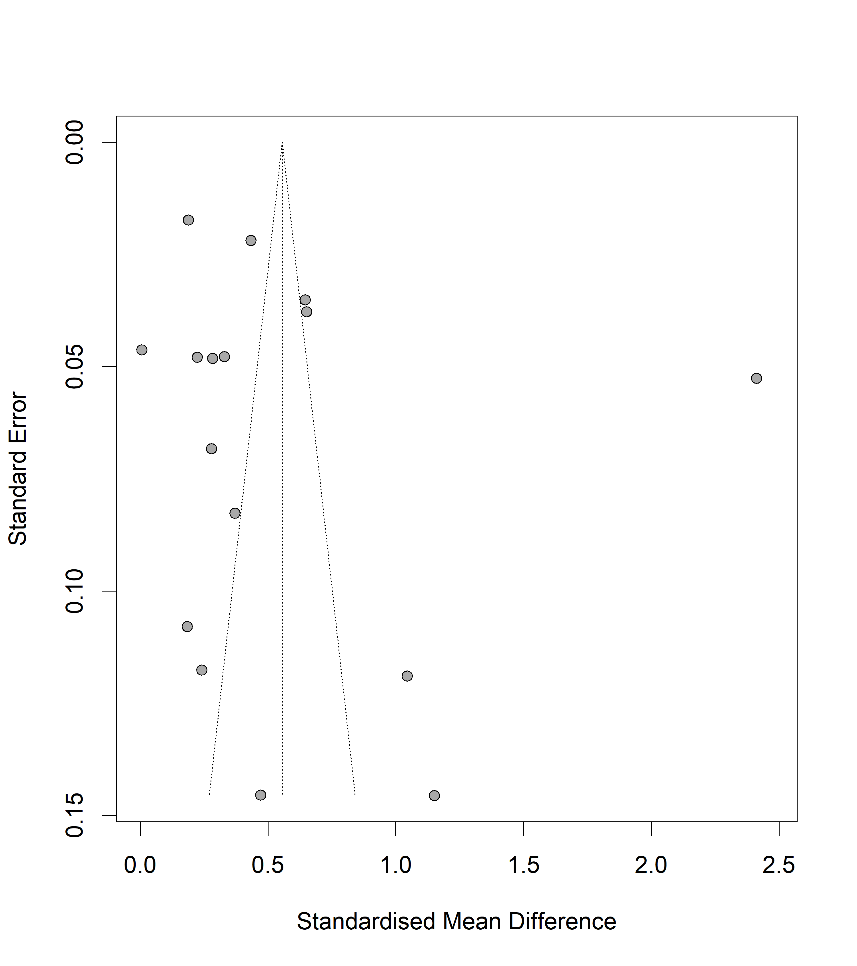


### Table S6.2.4.1: Egger’s test – Mental Health with all studies

*Eggers' test of the intercept*

| intercept | 95% CI | t | p |
| --- | --- | --- | --- |
| 5.186 | [-4.61; 14.98] | 1.037 | 0.317 |

Eggers' test does not indicate the presence of funnel plot asymmetry.

*P-curve analysis*

- Total number of provided studies: k = 16

- Total number of p<0.05 studies included into the analysis: k = 14 (87.5%)

- Total number of studies with p<0.025: k = 13 (81.25%)

### Figure S6.2.4.3: Forest plot – Mental Health without outliers


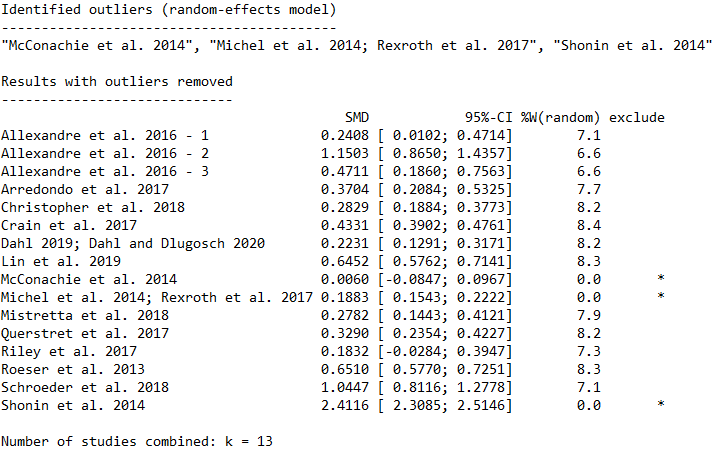


## 2.5 Stress

### Figure S6.2.5.1: Forest plot – Stress with all studies


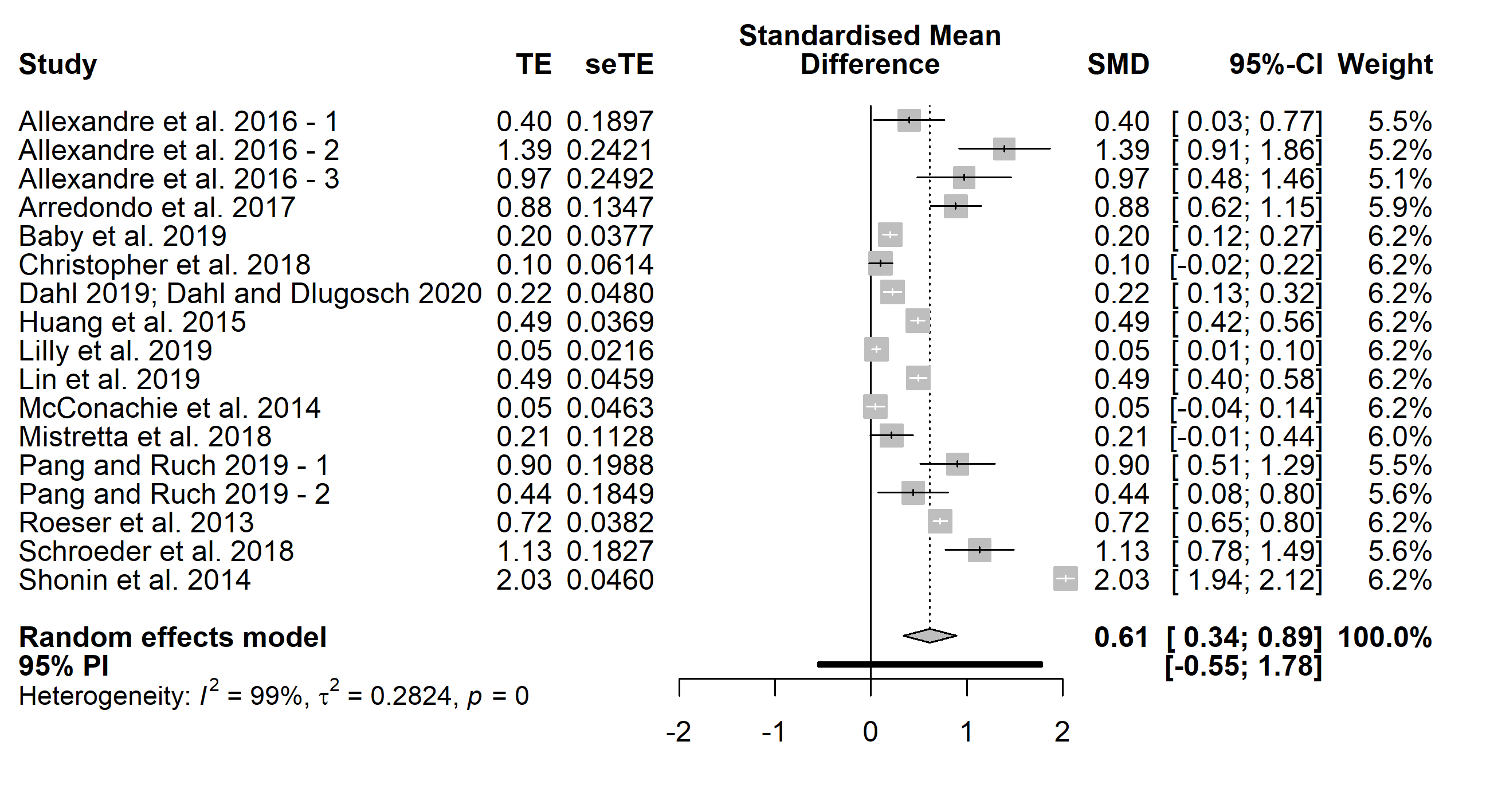


### Figure S6.2.5.2: Funnel plot – Stress with all studies


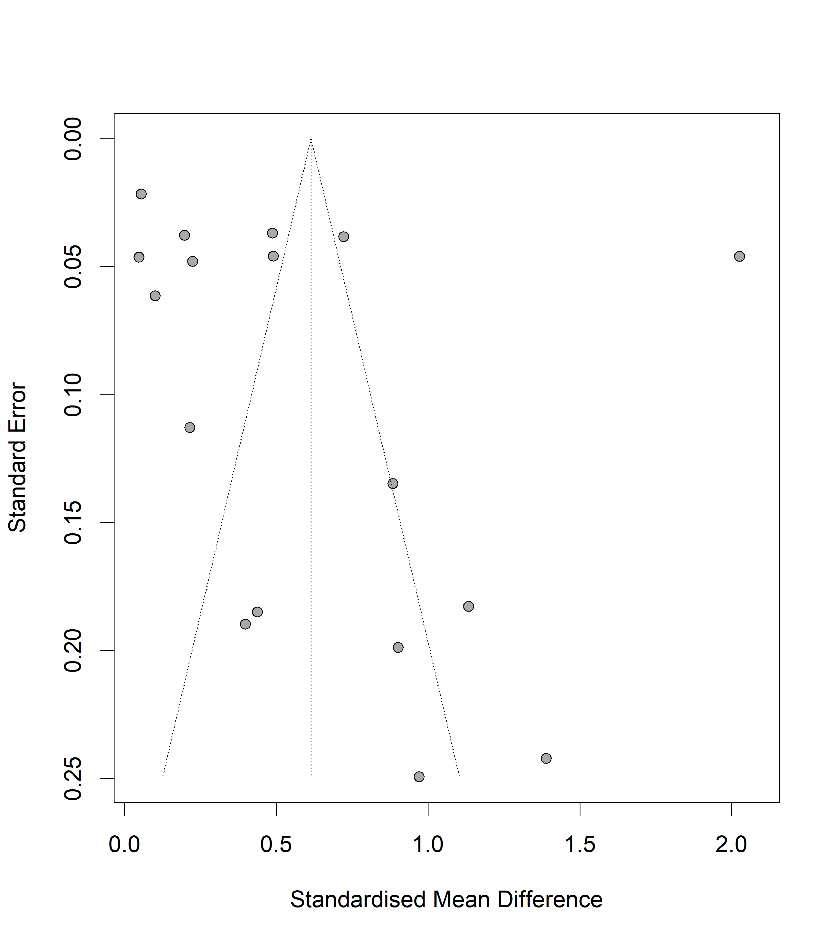


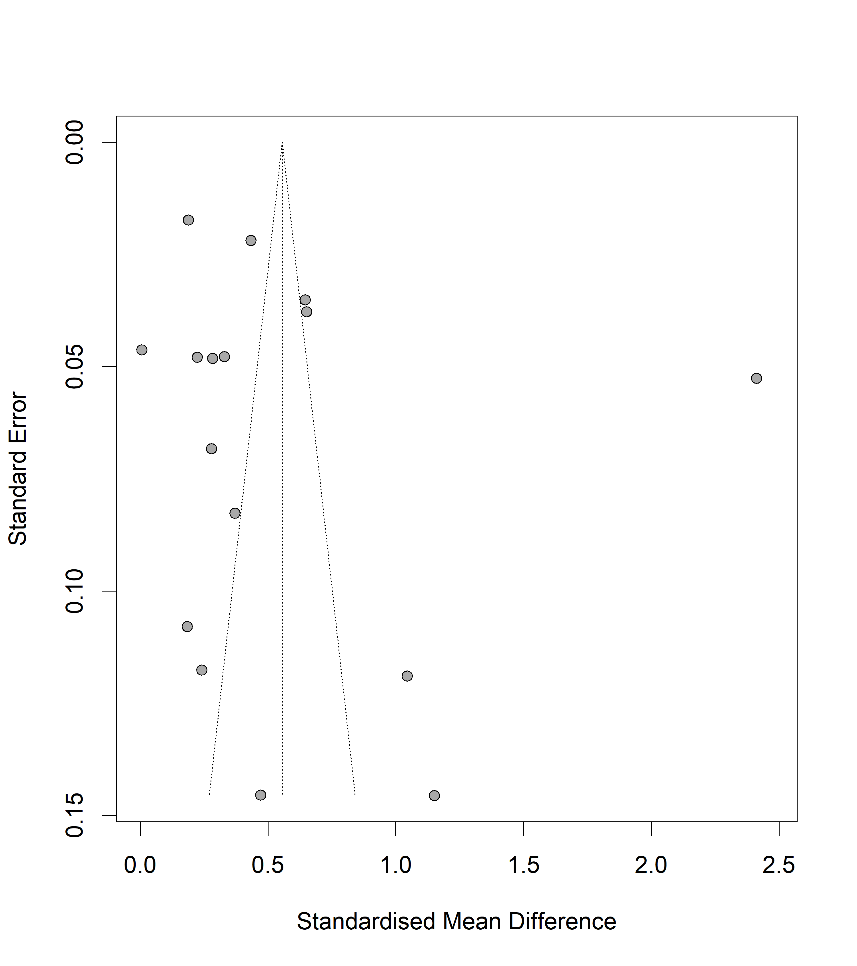


### Table S6.2.5.1: Egger’s test – Stress with all studies

*Eggers' test of the intercept*

| intercept | 95% CI | t | p |
| --- | --- | --- | --- |
| 4.706 | [-3.87;13.28] | 1.075 | 0.299 |

Eggers' test does not indicate the presence of funnel plot asymmetry.

*P-curve analysis*

- Total number of provided studies: k = 17

- Total number of p<0.05 studies included into the analysis: k = 14 (82.35%)

- Total number of studies with p<0.025: k = 13 (76.47%)

### Figure S6.2.5.3: Forest plot – Stress without outliers


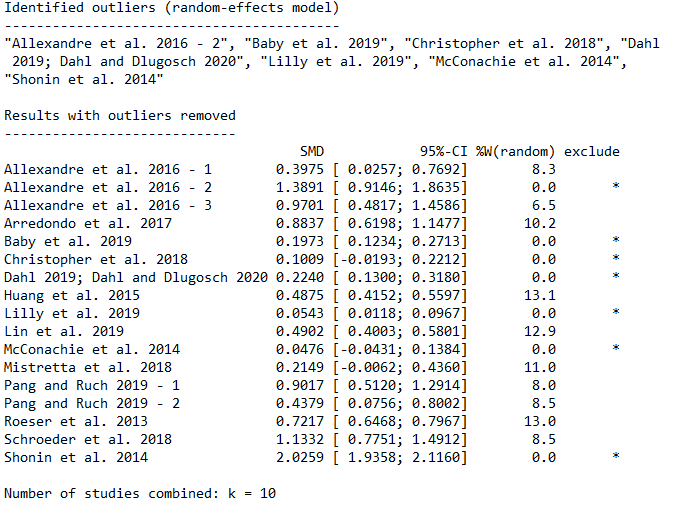


## 2.6 Resilience

/

## 2.7 Work-related factors

### Figure S6.2.7.1: Forest plot – Work-related factors with all studies


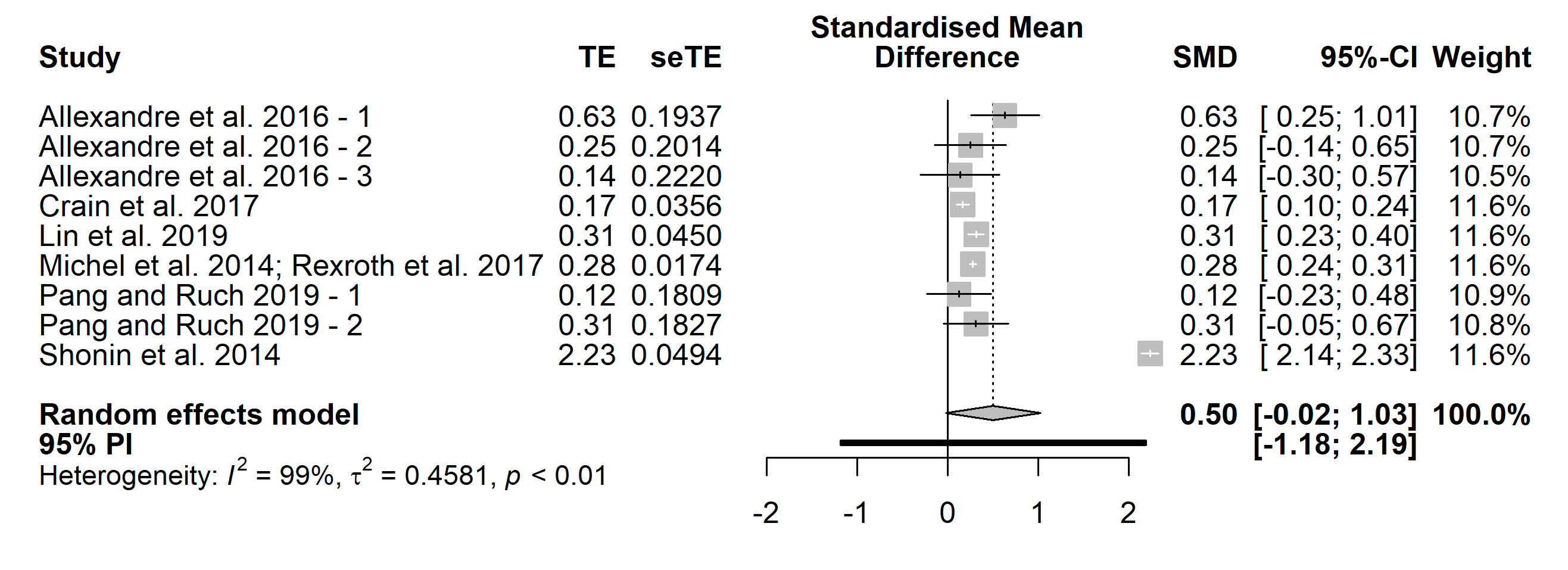


### Figure S6.2.7.2: Funnel plot – Work-related factors with all studies


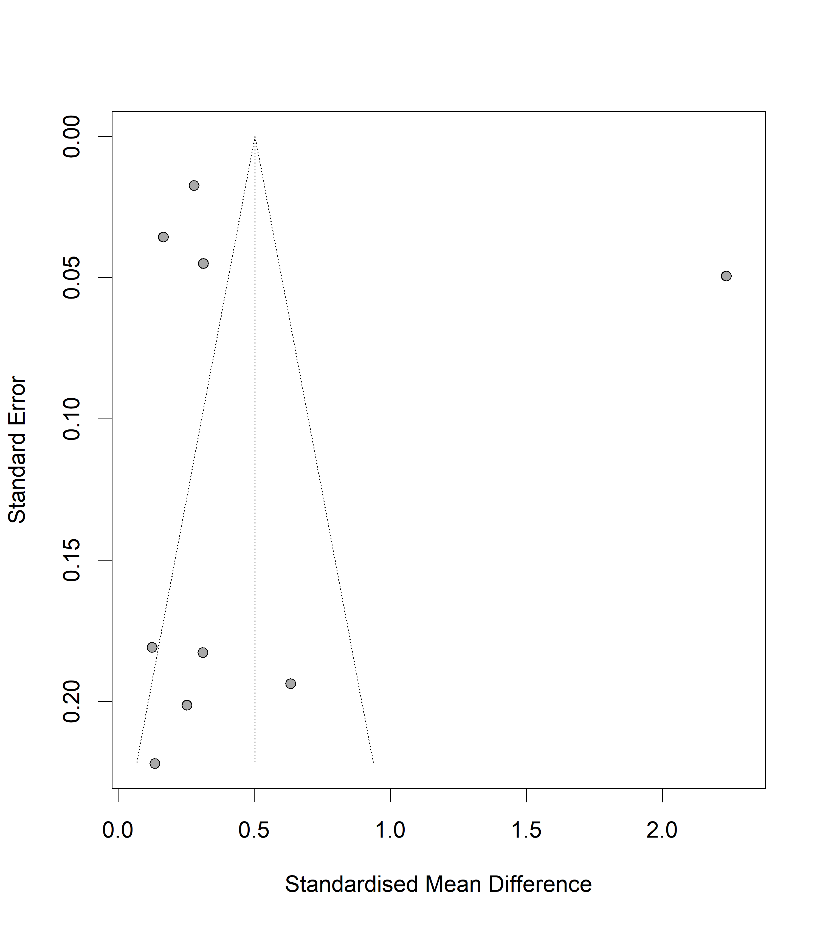


### Table S6.2.7.1: Egger’s test – Work-related factors with all studies

*Eggers' test of the intercept*

| intercept | 95% CI | t | p |
| --- | --- | --- | --- |
| 3.559 | [-9.77;16.89] | 0.523 | 0.617 |

Eggers' test does not indicate the presence of funnel plot asymmetry.

*P-curve analysis*

- Total number of provided studies: k = 9

- Total number of p<0.05 studies included into the analysis: k = 5 (55.56%)

- Total number of studies with p<0.025: k = 5 (55.56%)

### Figure S6.2.7.3: Forest plot – Work-related factors without outliers


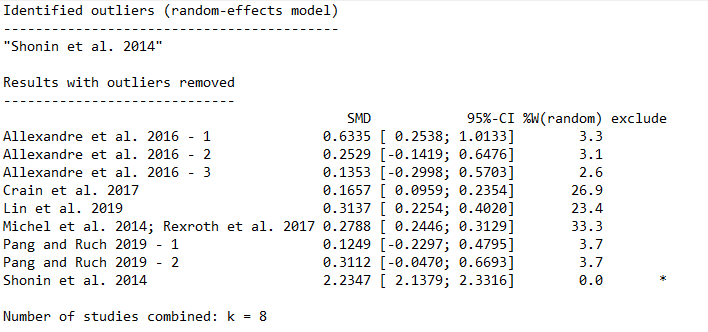


# Long-term follow-up

## 3.1 Mindfulness

/

## 3.2 Well-being

### Figure S6.3.2.1: Forest plot – Well-being with all studies


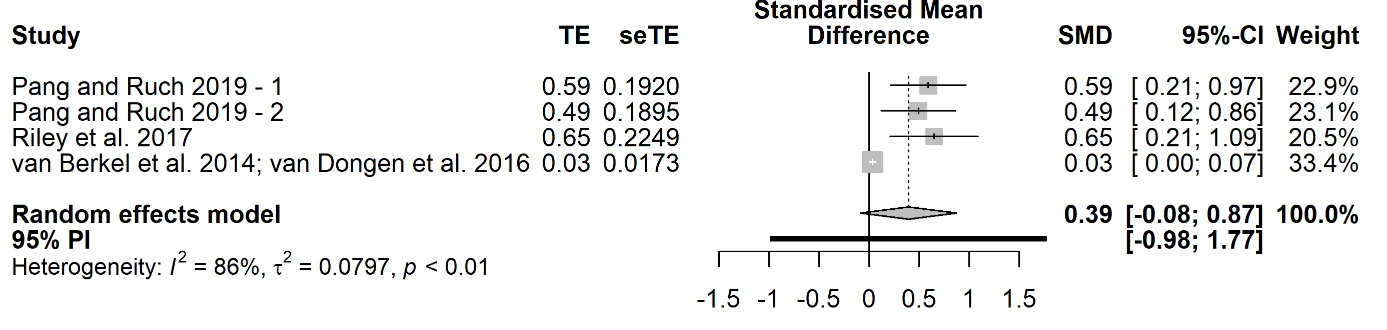


### Figure S6.3.2.2: Funnel plot – Well-being with all studies


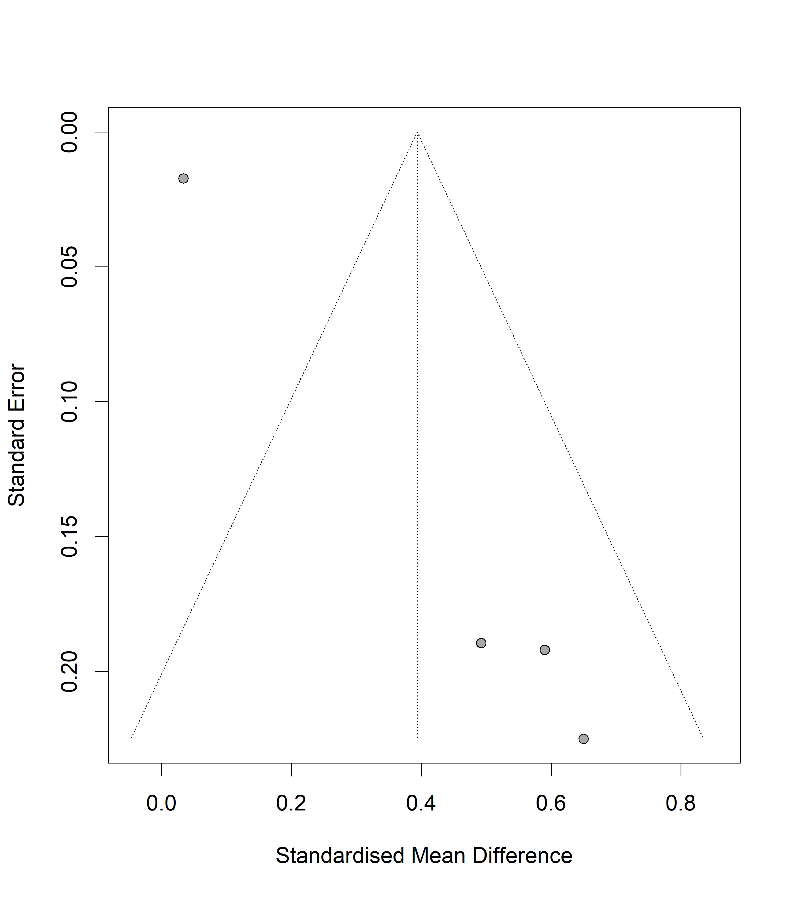


### Table S6.3.2.1: Egger’s test – Well-being with all studies

*Eggers' test of the intercept*

| intercept | 95% CI | t | p |
| --- | --- | --- | --- |
| 2.938 | [2.64; 3.24] | 19.22 | 0.003 |

Eggers' test does indicate the presence of funnel plot asymmetry.

*P-curve analysis*

- Total number of provided studies: k = 4

- Total number of p<0.05 studies included into the analysis: k = 3 (75%)

- Total number of studies with p<0.025: k = 3 (75%)

## 3.3 Physical Health

### Figure S6.3.3.1: Forest plot – Physical Health with all studies


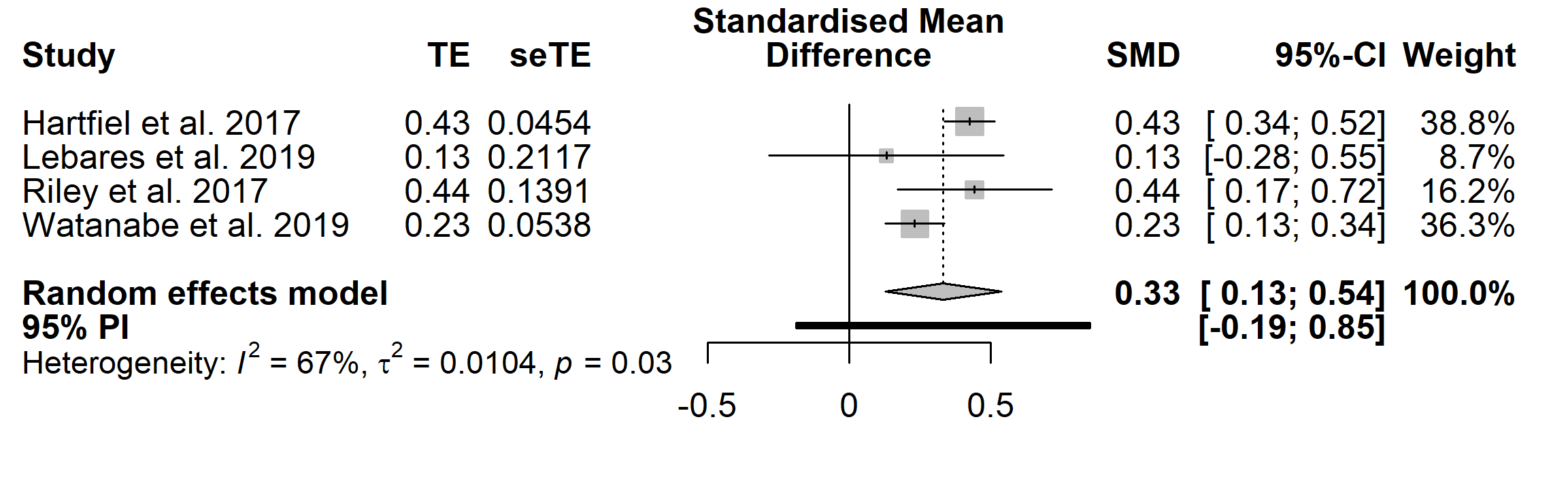


### Figure S6.3.3.2: Funnel plot – Physical Health with all studies


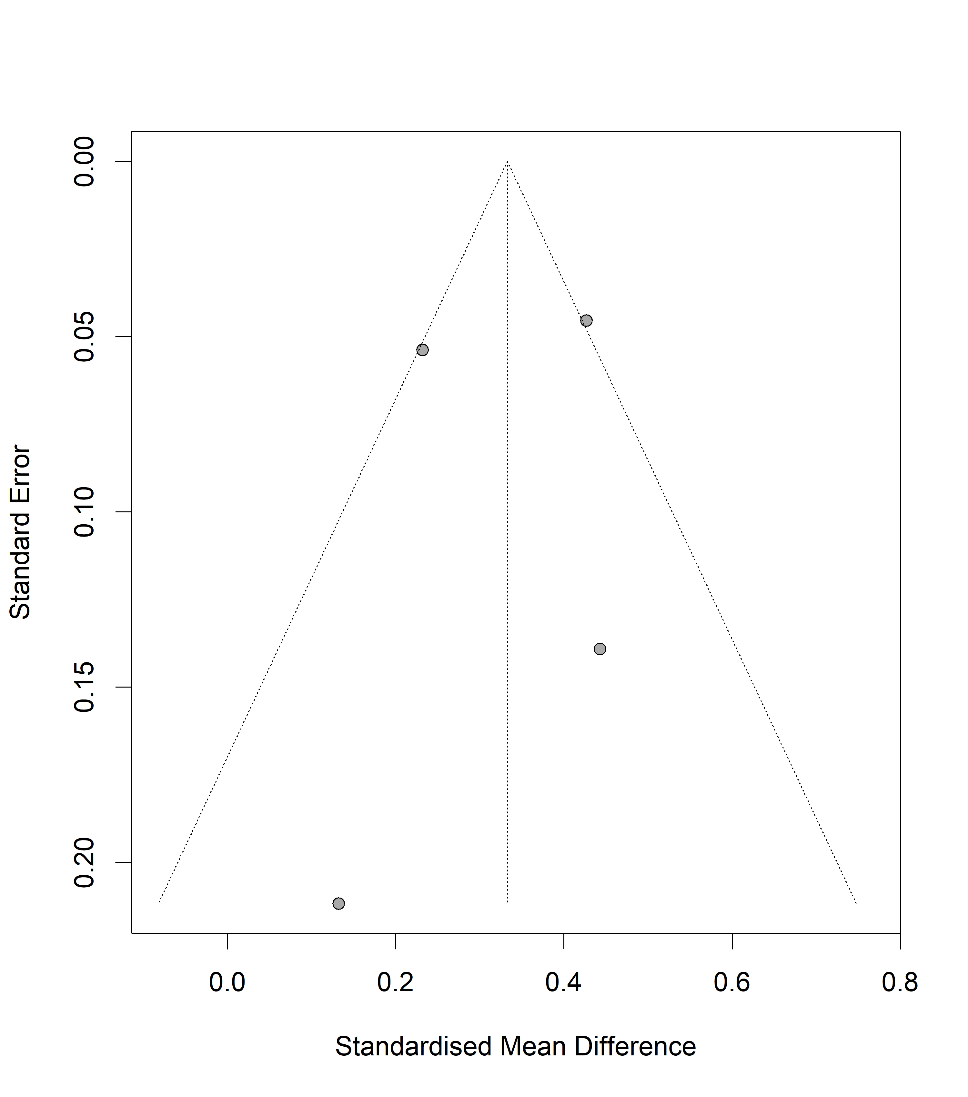


### Table S6.3.3.1: Egger’s test – Physical Health with all studies

*Eggers' test of the intercept*

| intercept | 95% CI | t | p |
| --- | --- | --- | --- |
| -0.691 | [-4.89;3.51] | -0.322 | 0.778 |

Eggers' test does not indicate the presence of funnel plot asymmetry.

*P-curve analysis*

- Total number of provided studies: k = 4

- Total number of p<0.05 studies included into the analysis: k = 3 (75%)

- Total number of studies with p<0.025: k = 3 (75%)

## 3.4 Mental Health

### Figure S6.3.4.1: Forest plot – Mental Health with all studies


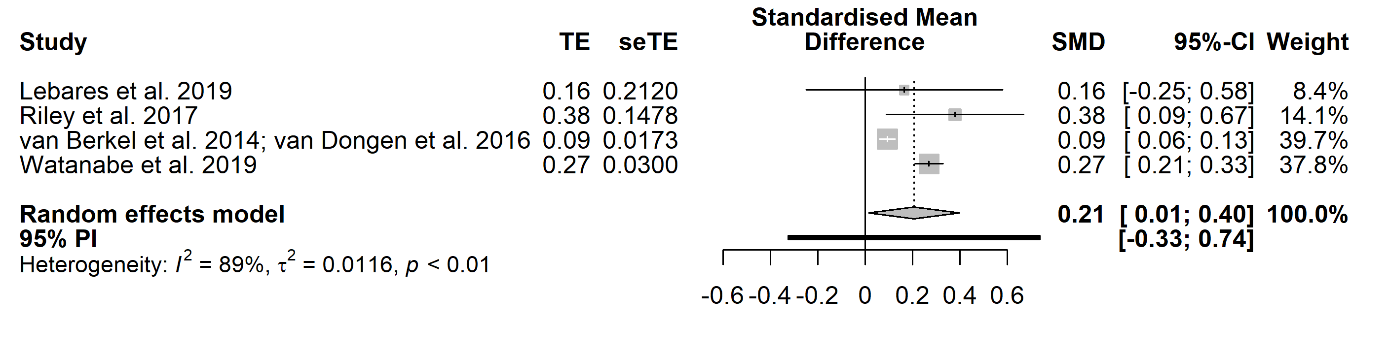


### Figure S6.3.4.2: Funnel plot – Mental Health with all studies


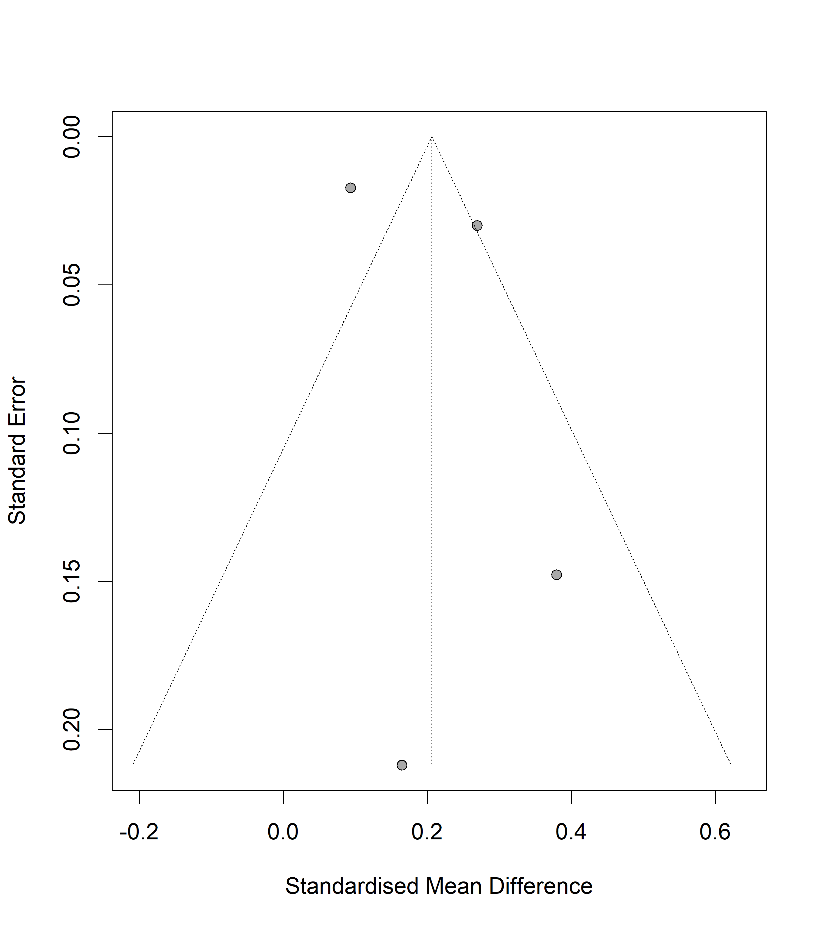


### Table S6.3.4.1: Egger’s test – Mental Health with all studies

*Eggers' test of the intercept*

| intercept | 95% CI | t | p |
| --- | --- | --- | --- |
| 2.013 | [-2.97; 7] | 0.792 | 0.511 |

Eggers' test does not indicate the presence of funnel plot asymmetry.

*P-curve analysis*

- Total number of provided studies: k = 4

- Total number of p<0.05 studies included into the analysis: k = 3 (75%)

- Total number of studies with p<0.025: k = 3 (75%)

## Stress

### Figure S6.3.5.1: Forest plot – Stress with all studies


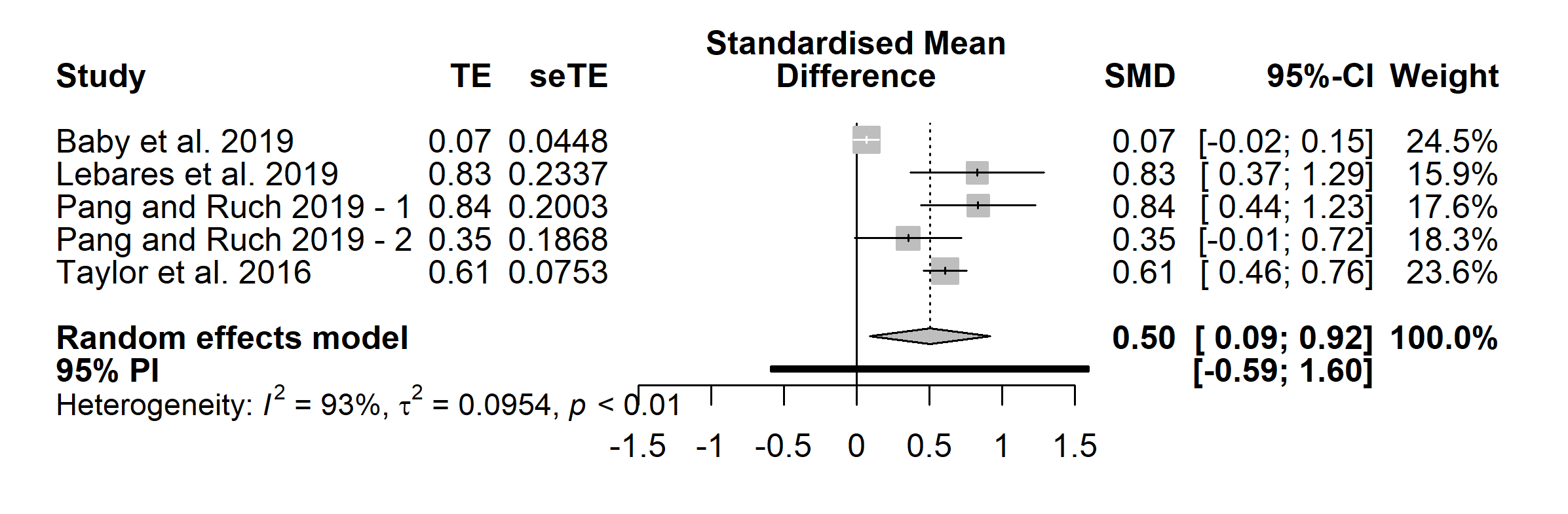


### Figure S6.3.5.2: Funnel plot – Stress with all studies


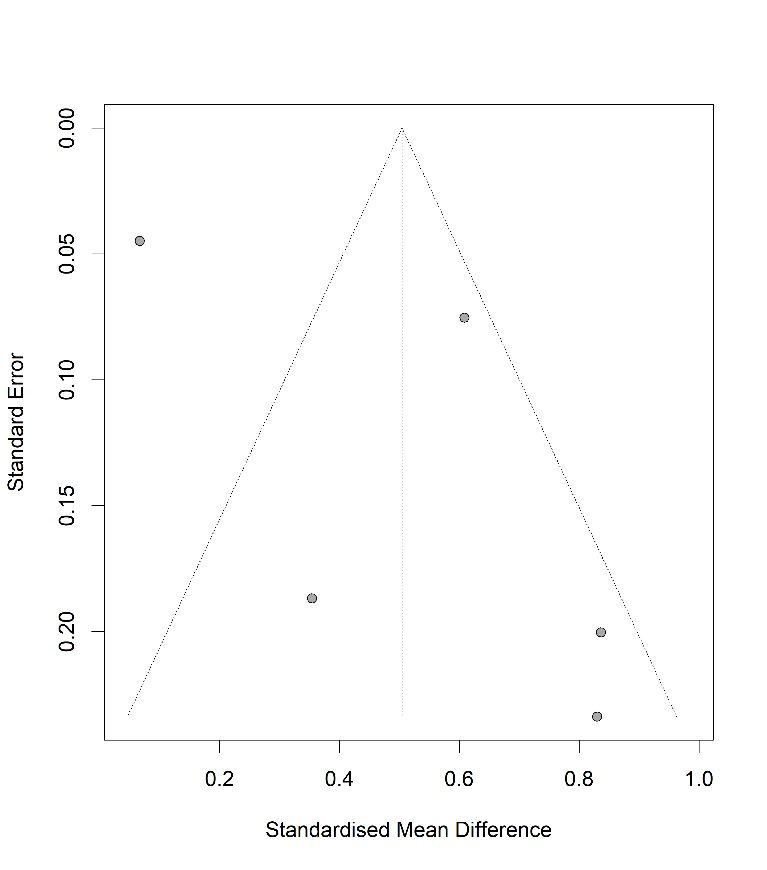


### Table S6.3.5.1: Egger’s test – Stress with all studies

*Eggers' test of the intercept*

| intercept | 95% CI | t | p |
| --- | --- | --- | --- |
| 4.089 | [-0.56; 8.73] | 1.725 | 0.183 |

Eggers' test does not indicate the presence of funnel plot asymmetry.

*P-curve analysis*

- Total number of provided studies: k = 5

- Total number of p<0.05 studies included into the analysis: k = 3 (60%)

- Total number of studies with p<0.025: k = 3 (60%)

## Resilience

/

## Work-related factors

### Figure S6.3.7.1: Forest plot – Work-related factors with all studies


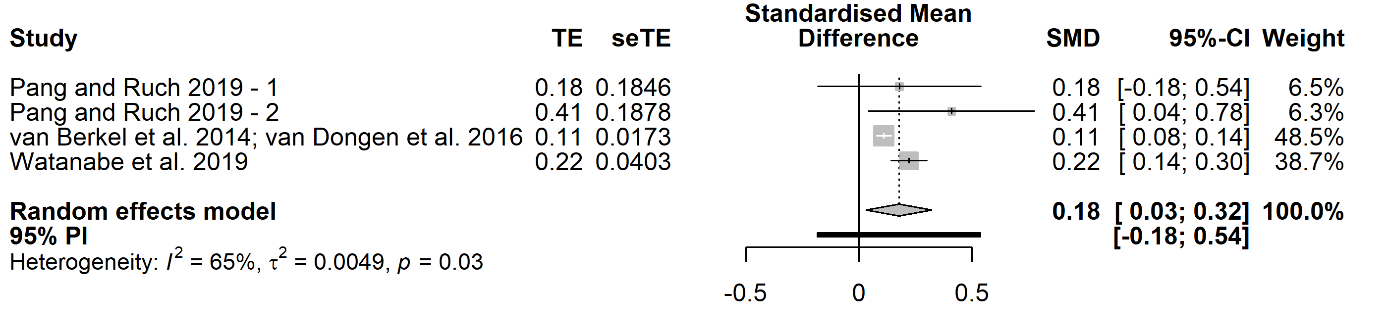


### Figure S6.3.7.2: Funnel plot – Work-related factors with all studies


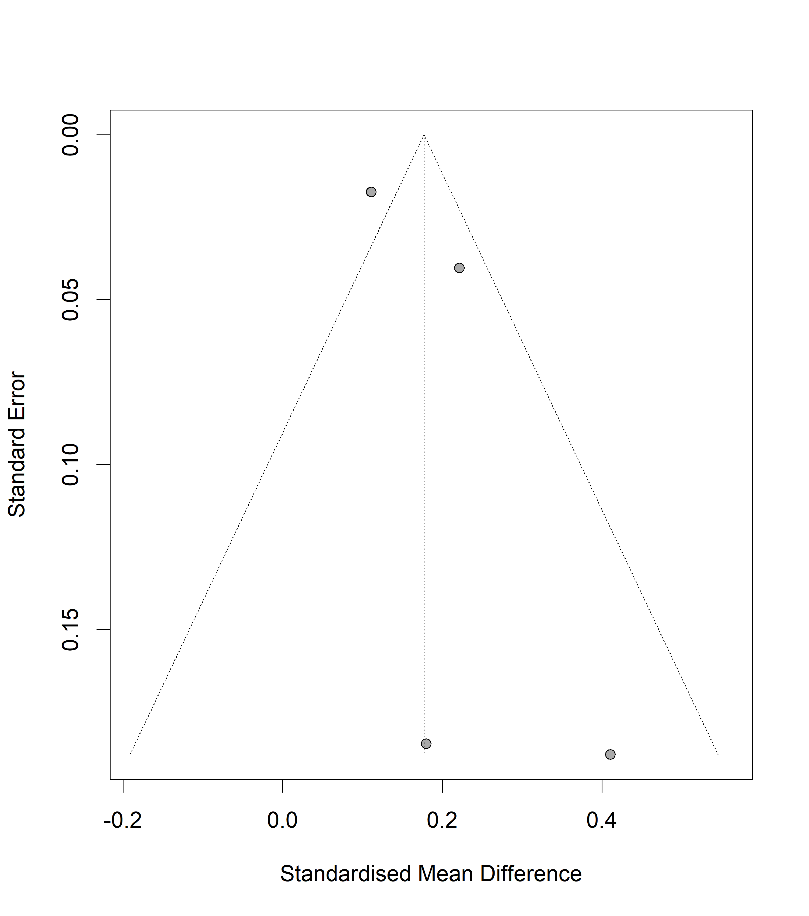


### Table S6.3.7.1: Egger’s test – Work-related factors with all studies

*Eggers' test of the intercept*

| intercept | 95% CI | t | p |
| --- | --- | --- | --- |
| 1.577 | [-0.5; 3.66] | 1.486 | 0.275 |

Eggers' test does not indicate the presence of funnel plot asymmetry.

*P-curve analysis*

- Total number of provided studies: k = 4

- Total number of p<0.05 studies included into the analysis: k = 3 (75%)

- Total number of studies with p<0.025: k = 2 (50%)
